# SeedSeg: image-based transgenic seed counting for segregation analysis of T-DNA loci

**DOI:** 10.1186/s13007-025-01406-4

**Published:** 2025-06-24

**Authors:** Santiago Hernández, Vivian Zhong, Jennifer A. N. Brophy

**Affiliations:** 1 Department of Computer Science, Stanford University, Stanford, CA 94305 USA; 2 Department of Bioengineering, Stanford University, Stanford, CA 94305 USA

**Keywords:** Seed counting, Image segmentation, Arabidopsis, Transgenic, Plant genetic engineering, Transgene segregation analysis

## Abstract

**Background:**

Transgenic plants are essential for both basic and applied plant biology. Recently, fluorescent and colorimetric markers were developed to enable nondestructive identification of transformed seeds and accelerate the generation of transgenic plant lines. Yet, transformation often results in the integration of multiple copies of transgenes in the plant genome. Multiple transgene copies can lead to transgene silencing and complicate the analysis of transgenic plants by requiring researcher to track multiple T-DNA loci in future generations. Thus, to simplify analysis of transgenic lines, plant researchers typically screen transformed plants for lines where the T-DNA inserted in a single locus — an analysis that involves laborious manual counting of fluorescent and non-fluorescent seeds for screenable markers.

**Results:**

To expedite T-DNA segregation analysis, we developed SeedSeg, an image analysis tool that uses a segmentation algorithm to count the number of transformed and wild-type seeds in an image. SeedSeg runs a chi-squared test to determine the number of T-DNA loci. Parameters can be adjusted to optimize for different brightness intensities and seed sizes.

**Conclusions:**

By automating the seed counting process, SeedSeg reduces the manual labor associated with identifying transgenic lines containing a single T-DNA locus. SeedSeg is adaptable to different seed sizes and visual transgene markers, making it a versatile tool for accelerating plant research.

**Supplementary Information:**

The online version contains supplementary material available at 10.1186/s13007-025-01406-4.

## Background

Plant transformation is essential for mechanistic studies of gene function and efforts to engineer improved crop varieties. Transgenic approaches allow researchers to delete or overexpress plant genes and analyze their impact on development, stress responses, and metabolic pathways [1–3]. They can also provide insight into gene regulation by enabling introduction of reporters that track where and when genes are expressed [4–7]. In agriculture, plant transformation plays a key role in the introduction of traits to crops that increase yield and nutritional content [8–10]. Since its development in 1983, plant transformation has become indispensable in both fundamental plant biology research and the creation of novel crop varieties [11].

Recent advances in fluorescent and colorimetric markers enable non-invasive identification of transgenic seeds, which streamlines the generation of transgenic plants [4, 12, 13]. FAST (fluorescence-accumulating seed technology) [12, 14–16] and *RUBY* markers [4, 17, 18] that color seeds with fluorescent proteins or pigments, respectively, enable identification of transgenic seeds without antibiotics or herbicides. This is advantageous because antibiotics and herbicides can adversely affect plant growth and confound phenotypic analysis of T1 plants and seeds. In contrast, screenable markers allow researchers to identify transgenic seeds without selectable agents and significantly shorten research timelines by analyzing phenotypes in the T1 generation. Seed markers have been developed for a variety of plants, including rice [14] and soybean [15] where they also enable non-destructive identification of transgenic seeds. Altogether, fluorescent and colorimetric markers have great potential for accelerating the generation of transgenic plants.

Establishing single-copy transgene lines is a critical step in developing transgenic crops and experimental models. The most common plant transformation methods often result in the integration of multiple transgene copies in the plant genome at a one or more locations [19, 20]. Multiple transgene insertions can cause transgene silencing and complicate the analysis of transgenic plants [19–23]. Thus, single-copy transgene lines– which are more likely to have stable gene expression across generations– are desirable for research and agricultural applications [24]. Single-copy and single locus transgenic lines also simplify genetic analysis because they follow a straightforward 3:1 Mendelian segregation pattern in the T2 generation. This predictability facilitates the identification of homozygous T3 lines, reducing the time and effort required for screening and ensuring consistent trait inheritance. As a result, strategies to efficiently identify single-insertion lines are essential for plant research and crop development.

Currently, *Agrobacterium spp.* serve as the main tools for introducing transgenes into plant cells. Though effective, *Agrobacterium-*mediated transformation can give rise to multiple transgene insertions, either through independent T-DNA integration events or by inserting multiple T-DNA copies in tandem at a single locus; Up to 85% of Arabidopsis transformants created via floral dip have been found to contain multiple transgene copies [25]. Multiple methods can be used to identify single-insertion lines, including whole genome sequencing, qPCR, and Southern blots. However, these methods can be costly and labor intensive. Thus, researchers often use segregation analysis to identify and eliminate multiple-loci T-DNA insertion events.

Segregation analysis tracks inheritance of a genetic trait across generations to infer transgene copy number, which requires identification and enumeration of transgenic and non-transgenic seed produced at each generation. For seed markers, this typically involves labor-intensive manual counting of marker + and marker- seeds under a stereomicroscope, followed by statistical analysis to determine inheritance patterns. This approach is tedious, prone to human error, and limits the scalability of studies involving transgenic plants. A more efficient analysis method could streamline this process and accelerate plant research.

To expedite transgene segregation analysis with fluorescent and colorimetric seed markers, we developed SeedSeg, a Python-based open-source image analysis tool that automatically counts the number of transformed and wild-type seeds in an image. After counting, SeedSeg runs a chi-squared test [26] to determine the number of transgene insertions. With SeedSeg, single-locus T-DNA lines can be rapidly identified by dumping T2 seeds into a weigh boat and taking a picture with a stereomicroscope. Seeds no longer need to be meticulously counted by hand to determine transgene segregation. SeedSeg is robust in its segmentation capabilities, able to distinguish and count seeds even when they appear to be overlapping in the image. Moreover, SeedSeg offers adjustable parameters to handle varying image conditions, including brightness, noise levels, and seed sizes, making it useful for a wide range of equipment setups, screenable markers, and plant varieties. SeedSeg can be run from both a web server and the command line. By automating seed counting and segregation analysis, SeedSeg provides a fast, accessible, and reliable tool for researchers generating transgenic plants.

## Implementation

SeedSeg is an image processing and segmentation tool that uses Python and OpenCV [27] to count the number of transgenic and non-transgenic seeds present in an image. For fluorescent transgene markers, SeedSeg requires researchers to capture both a brightfield and a fluorescent image of a field of seeds. Brightfield images are used to detect and segment all seeds within the field, while fluorescent images are used to identify seeds expressing the transgenic marker, such as FastRed [12]. Once counted, T-DNA loci number is determined by comparing the observed ratio of transgenic to non-transgenic seeds to the expected 3:1 Mendelian segregation pattern using a chi-squared statistical test. The steps used to automate seed counting and transgene segregation analysis are described below.

**Fig. 1:**
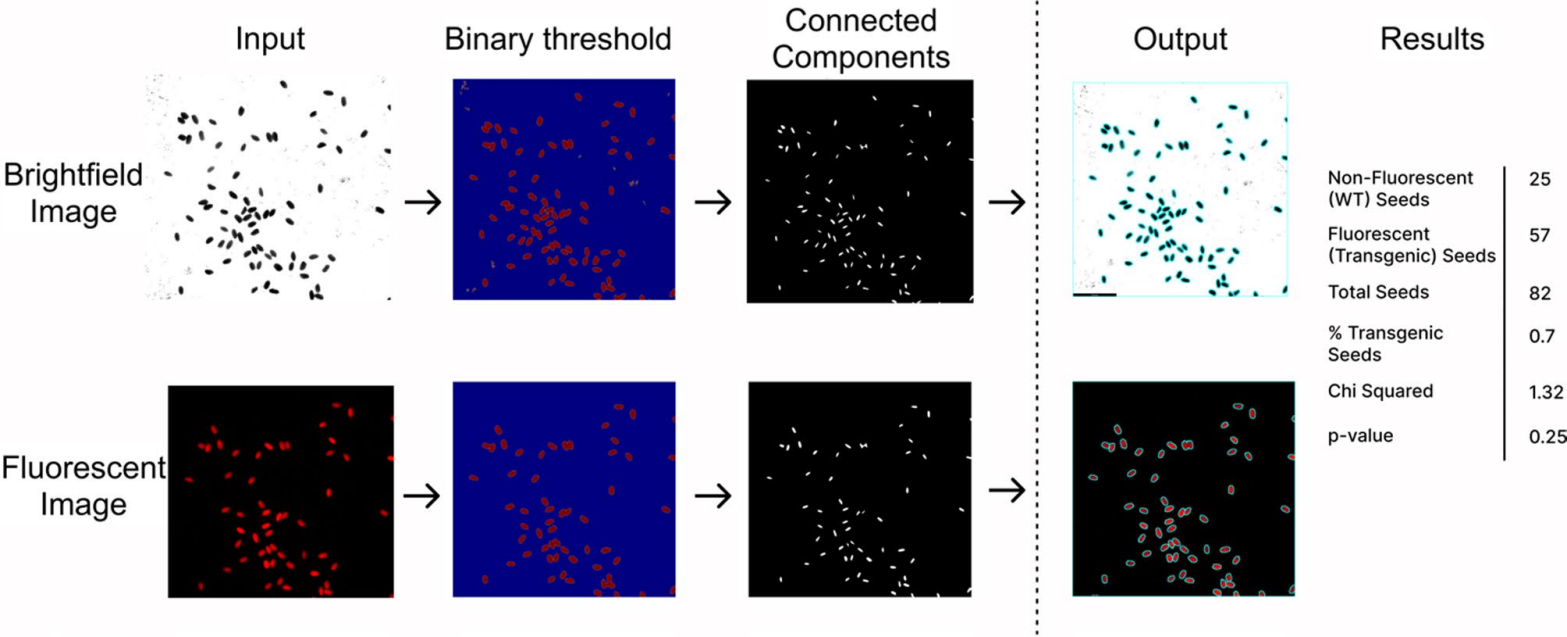
SeedSeg image processing pipeline. Brightfield (top) and fluorescent (bottom) images undergo multiple processing steps: (1) conversion to grayscale, (2) application of a binary threshold to isolate seed regions, (3) connected components analysis to identify and label individual seeds, and (4) output visualization. This example is of a T2 transgenic line expressing FastRed in a Mendelian segregation pattern, indicating single-transgene insertion

In the fluorescence mode, brightfield and fluorescent seed images undergo segmentation to count seeds and distinguish between transgenic and non-transgenic seeds. An overview of the image processing and analysis pipeline is shown in Fig. 1. First the brightfield and fluorescent images are converted to grayscale to standardize the input data. Brightfield images are inverted to enhance contrast and improve seed detection. Then, scale bars are removed to prevent interference with the seed counting process. Processed images are segmented to count seeds. SeedSeg’s segmentation process begins with an initial brightness threshold, which is an adjustable parameter that isolates bright regions corresponding to the seeds by converting the grayscale image into a binary format. In this format, pixels representing seeds are highlighted against a dark background. Following this, connected components analysis is used to label each distinct seed region and features such as the area and centroid of each seed are extracted for further analysis. SeedSeg uses the median area of detected seeds to dynamically adjust the radial threshold parameter, which helps ensure proper separation of overlapping seeds and enables adaptation of the program to plant species with different seed sizes. The user can either set the radial threshold parameter directly, or set the more intuitive radial threshold ratio parameter, from which the radial threshold is calculated. A radial threshold ratio of 0.4 means that the recognized seed areas are reduced by 40% to separate touching seeds. After seed detection, SeedSeg applies a noise filtering step to remove small regions below a set radial threshold. Filtering removes non-seed regions from the image and reduces the number of false positives created by dirt or other artifacts. These steps enable robust detection of seeds in both brightfield and fluorescent images.

To ensure accurate seed counting, SeedSeg implements additional steps to enhance seed detection. First, morphological operations (erosion, dilation, and opening) are applied to remove small artifacts and smooth seed region boundaries to ensure they are clearly defined. Second, a Chamfer Distance transform [28] is applied to calculate the shortest distance from each pixel to the background. The distance transform step helps separate seeds that are close together or slightly overlapping. Finally, to separate individual seeds that are tightly clustered or overlapping, SeedSeg employs a watershed algorithm [29]. The watershed algorithm treats the grayscale image as a topographical surface, where markers placed at seed centroids act as “watersheds.” This allows the algorithm to identify the borders between overlapping seeds. Application of the watershed algorithm improves the accuracy of seed counting and generates visual output of contours drawn around each detected seed to aid in verification of segmentation results. SeedSeg integrates these segmentation techniques to ensure reliable seed detection and counting.

The colorimetric *RUBY* reporter can be used as a seed marker for screening without fluorescence imaging equipment. SeedSeg can identify and count *RUBY*-expressing seeds. SeedSeg’s colorimetric mode requires an RGB image with a plain white background. The image is converted to the CIELAB color space, which expresses color as three values: L* for lightness, a* for the green-red axis, and b* for the blue-yellow axis. The L* value is first used to threshold for all seeds against the background. The b* value is then used to distinguish the yellow wild-type seeds which have a high b* value from the red *RUBY* seeds which have a low b* value. Radial thresholds and the watershed algorithm are then applied as described above for the fluorescence mode.

After analyzing images to count seeds, SeedSeg performs a statistical test to assess whether the transgene loci are segregating at the expected Mendelian ratio. Single-locus T-DNA events should result in a T2 generation that exhibits Mendelian segregation with a 3:1 ratio of transformed:WT seeds. To determine whether a given transgenic line contains a single T-DNA insertion locus, SeedSeg runs the scipy.stats implementation of Pearson’s chi-squared test [26]. This is a *p*-value test used to assess the null hypothesis that the observed frequencies are obtained from independent sampling of a categorical distribution with given expected frequencies [30]. Using the observed seed counts from each of the fluorescence and brightfield images, SeedSeg tests the null hypothesis that the number of fluorescent seeds in the sampled T2 transgenic line of interest is 75% of the total number of seeds. The lower the resulting *p*-value, the greater the likelihood that the ratio of transformed: WT seeds in the transgenic line diverges from 3:1. *P*-values < 0.05 are assumed to indicate multiple independently segregating T-DNA loci or transgene silencing. Double-insertion events would result in a ratio of 15:1, while transgene silencing can result in a perceived ratio of less than 3:1. As previously discussed, this segregation analysis cannot rule out multiple-copy transgenic lines where the transgenes inserted in closely linked loci of the genome, such is the case with tandem T-DNA insertions [19, 20].

## Results and discussion

**Fig. 2:**
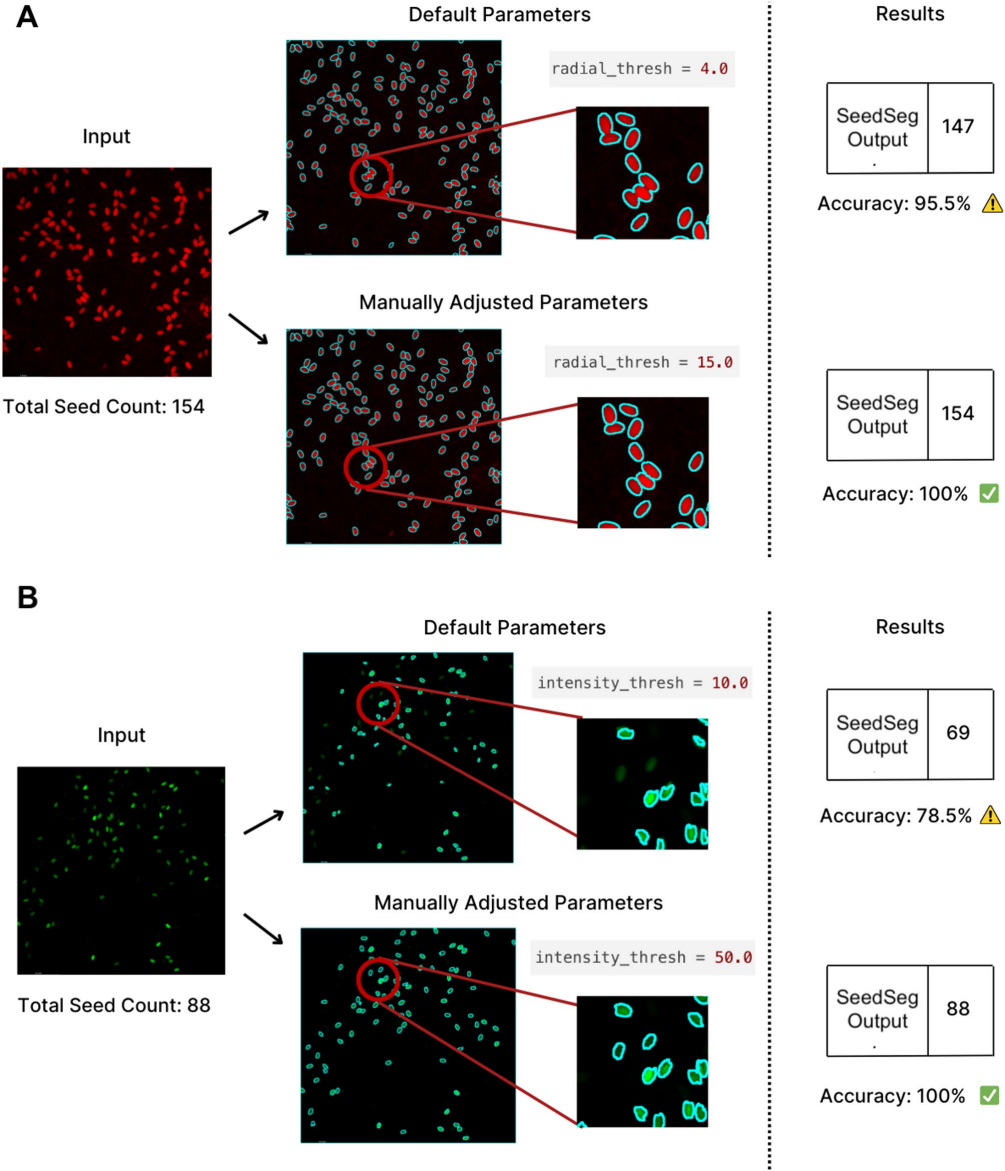
SeedSeg parameter tuning. SeedSeg image analysis parameters can be tuned to improve seed detection. (**A**) Results with default parameters (top) and adjusted parameters (bottom) demonstrate how parameter tuning can reduce errors that result from overlapping seeds. In this example, radial threshold was increased from 4.0 to 15.0. (**B**) Adjusting parameters to compensate for low seed brightness. Increasing the intensity threshold results in successful segmentation of all seeds in the image

To enable adoption of SeedSeg, we developed a user-friendly web interface (https:://seedseg-brophy.streamlit.app/). This interface was developed with Streamlit [31]. It allows researchers to upload and process images in real-time with interactive results visualization. The online platform simplifies seed analysis by eliminating the need to download and install software. Users upload seed images and SeedSeg automatically generates a table displaying seed counts and chi-squared test results. The web interface supports manual review and adjustment of image intensity and radius thresholds to optimize seed detection and provide a simple, user-friendly options for optimization (Fig. 2). SeedSeg parameters can be adjusted to capture faint signals from dim seeds, to prevent oversaturation of signal from bright seeds, and to fine-tune sensitivity to seed size for robust separation of overlapping seeds during segmentation. Users can export their results in CSV format for analysis offline and integration with other data tools. SeedSeg’s web interface makes seed analysis easy for researchers interested in automating segregation analysis.

We benchmarked SeedSeg performance on images of Arabidopsis seeds expressing the FastRed marker. A single layer of ~ 50–200 *Arabidopsis* seeds were poured onto a white paper weight boat for imaging. Seed images were collected using a Leica THUNDER widefield fluorescence microscope (Supplementary Data File). Fluorescent images were captured using the ET mCherry filter (Leica 10450195, Ex560/40, Em630/75) with 100 ms exposure. Brightfield images were collected using transmitted light with 100 ms exposure, 60% intensity, and 50% aperture. To evaluate SeedSeg’s accuracy and sensitivity to each parameter, we varied the microscope settings and compared true seed counts (obtained manually) to the number of seeds identified by SeedSeg (Suppl. Figure 1). Mean percent error (abs(observed_seed_count– actual_seed_count)/actual_seed_count) across fluorescent images was lowest with a radial threshold of 9.0 (or radial threshold ratio of 0.45) and an intensity threshold of 13.0. For brightfield images, percent error was lowest with a radial threshold of 10.0 (or radial threshold ratio of 0.50) and an intensity threshold of 29.0. The parameter space that yields accurate results is broader for brightfield images than fluorescence images. For 27/28 of tested images, there existed a parameter setting which could reduce the error to zero (Suppl. Table 1). These results demonstrate that SeedSeg can be used to achieve highly accurate seed segmentation and counting on Arabidopsis transgenic seeds.

**Fig. 3:**
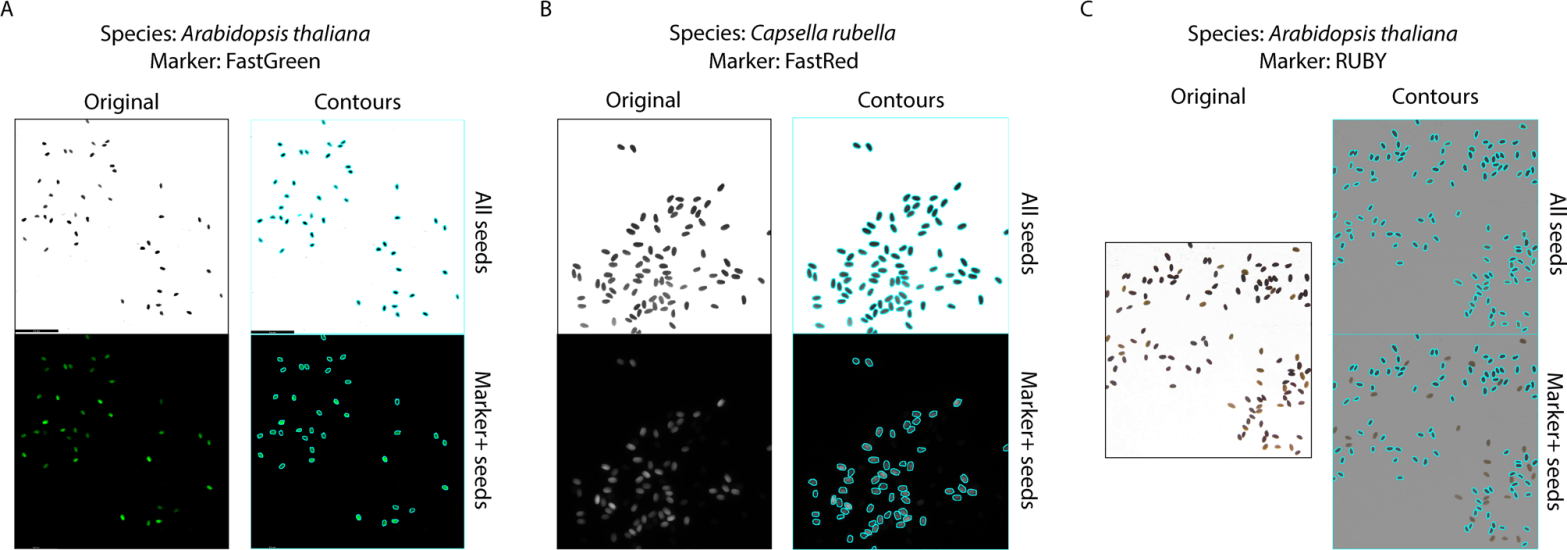
SeedSeg analysis of seeds expressing different transgene markers and from different species. (A) FastGreen-marker and wild-type Arabidopsis Col-0 seeds. Thresholds were set to brightfield intensity of 2.0, fluorescence intensity of 11.0, radial ration of 0.4. (B) FastRed-marker and wild-type Capsella rubella seeds. Thresholds were set to brightfield intensity of 2.0, fluorescence intensity of 30.0, radial ratio of 0.6. (C) RUBY-marker and wild-type Arabidopsis Col-0 seeds. Radial ratio threshold was set to 0.4 while brightfield intensity threshold was automatically set at 101.0. Raw sample images available in Supplementary Data File 2.

SeedSeg can be used perform segregation analysis on seeds expressing FastRed, FastGreen, or RUBY markers (Fig. 3). To demonstrate the versatility of SeedSeg, we analysed Arabidopsis seeds expressing FastGreen– a seed-specific green fluorescent protein marker– in fluorescence mode. The brightfield image was captured with transmitted light at 100% intensity, 100% aperture, and 50ms exposure; the fluorescence image was captured with the ET GFP filter (Leica 10447408, Ex470/40, Em525/50) at 500ms exposure. SeedSeg was able to accurately identify and count the transgenic seeds in the image. Similarly, we used the colorimetric mode of SeedSeg to analyze seeds containing pigment-based transgene marker *RUBY*. *RUBY*-expressing *Arabidopsis* seeds were viewed using the Leica THUNDER microscope with transmitted light at 100% intensity and 25% aperture, and an RGB image was captured with an iPhone through the eyepiece. Exposure was digitally maximized to remove spots resulting from imperfections in the paper weigh boat. SeedSeg’s colorimetric mode successfully differentiated between the lighter, yellow wild-type seeds and the darker, red *RUBY* seeds.

Additionally, SeedSeg is compatible with multiple plant species. We analyzed a sample of *Capsella rubella* seeds expressing FastRed (Fig. 3). Capsella rubella seed area is about four times larger than *Arabidopsis*, yet the software can still accurately count these seeds. The brightfield image was captured with transmitted light at 100% intensity, 25% aperture, and 500ms exposure; the fluorescence image was captured with the ET mCherry filter at 200 ms exposure.

SeedSeg offers several advantages over other image analysis softwares. PlantCV– a well-documented and comprehensive Python package that can be used for seed counting [32]– underperforms relative to SeedSeg on seed segmentation. PlantCV uses a binary threshold to identify seeds, which can effectively identify individual seeds, but can fail to distinguish adjacent seeds as distinct entities. SeedSeg’s watershed threshold facilitates independent identification of adjacent seeds. PlantCV had a mean percent error of 1.31% for brightfield images and 4.53% for fluorescence images, compared to 0.00% and 0.22% respectively using SeedSeg (Suppl. Table 1). ImageJ/Fiji can also be used to automate seed counting without user coding experience [33]. ImageJ can implement a watershed threshold; however, unlike SeedSeg, ImageJ does not by default perform batch processing. Other dedicated image-based seed counting tools were developed for measuring yield and fitness; these have not been demonstrated to be compatible with grayscale images, as is necessary for fluorescent seeds [34, 35]. SeedSeg’s web interface makes it easy for users to analyze their seeds by: (1) automatically setting the size threshold using the median seed size in the image, instead of requiring user trial and error; (2) automatically outlining segmented seeds so that users can easily verify segmentation results; (3) enabling easy manual adjustment of seed counts; (4) automatically performing chi-square tests to identify transgenic lines with single-locus T-DNA insertions. Thus, SeedSeg provides an accessible, accurate, and efficient tool for researchers working with screenable markers in seeds.

## Conclusions

By automating the seed counting process, SeedSeg reduces manual labor required to identify transgenic plant lines with single-locus T-DNA insertions. SeedSeg’s robust segregation analysis can be used to accurately count both transgenic and non-transgenic seeds in a large field of view, thereby automating a necessary step in the generation of homozygous transgenic plant lines. SeedSeg is adaptable to different seed sizes and visual transgene markers. Altogether, SeedSeg is a versatile tool for accelerating plant research.

## Electronic supplementary material

Below is the link to the electronic supplementary material.

Supplementary Material 1

Supplementary Material 2

Supplementary Material 3

## Data Availability

The code for this project is open-source and is accessible for download from https://github.com/santiaghini/seedseg/tree/main. The project includes instructions for setup, as well as examples on how to tune parameters. The SeedSeg web-interface can be accessed at: https://seedseg-brophy.streamlit.app/
