# Supplementary figures and images for: SeedSeg: image-based transgenic seed counting for segregation analysis of T-DNA loci
Source: Plant Methods. 2025 Jun 24;21:87. doi: 10.1186/s13007-025-01406-4 (PMC12186423; doi:10.1186/s13007-025-01406-4)

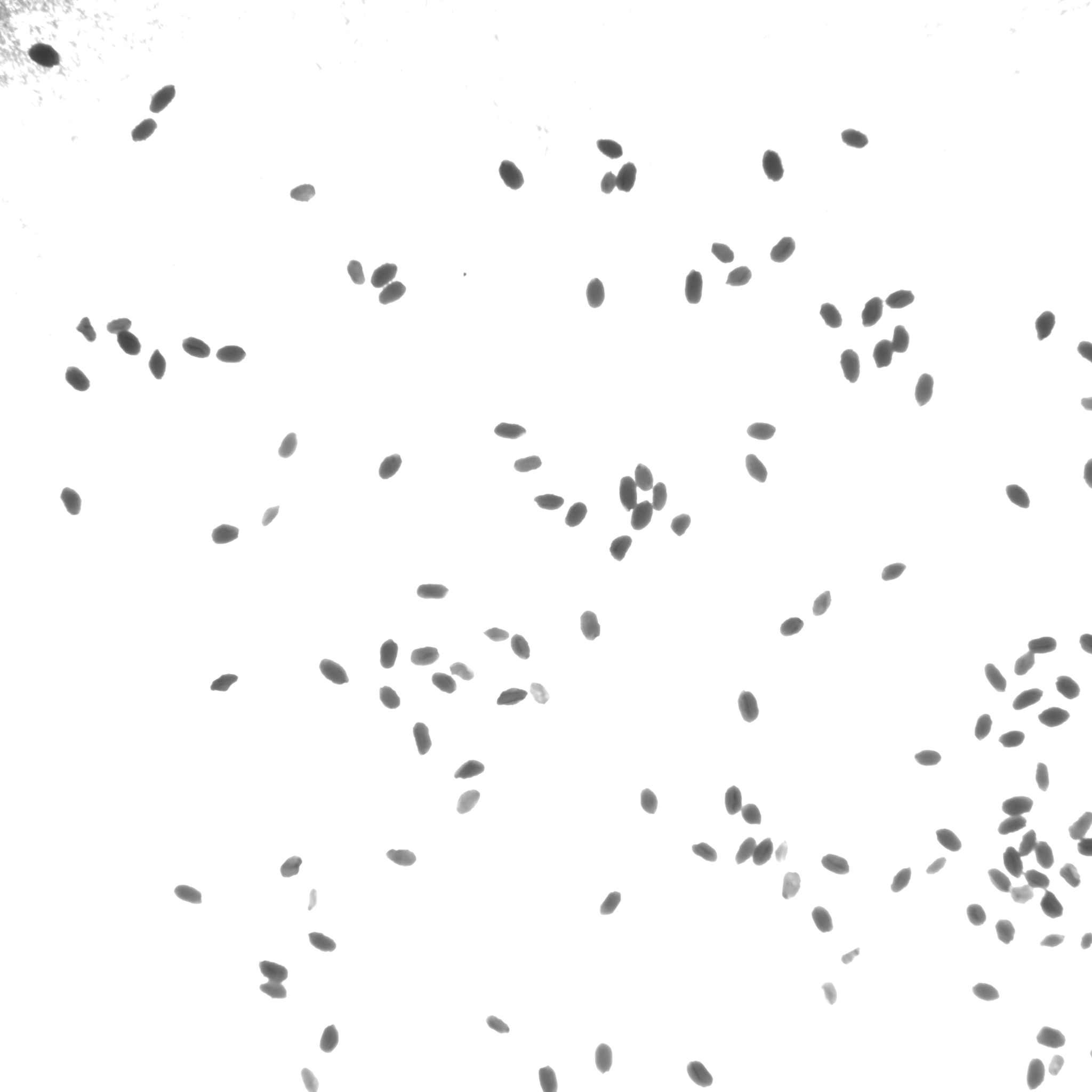

Supplement: Supplementary file 1 — Supplementary Material 1 [file 13007_2025_1406_MOESM1_ESM.zip › performance_comparison_images/VZ314-1_BF.tif]

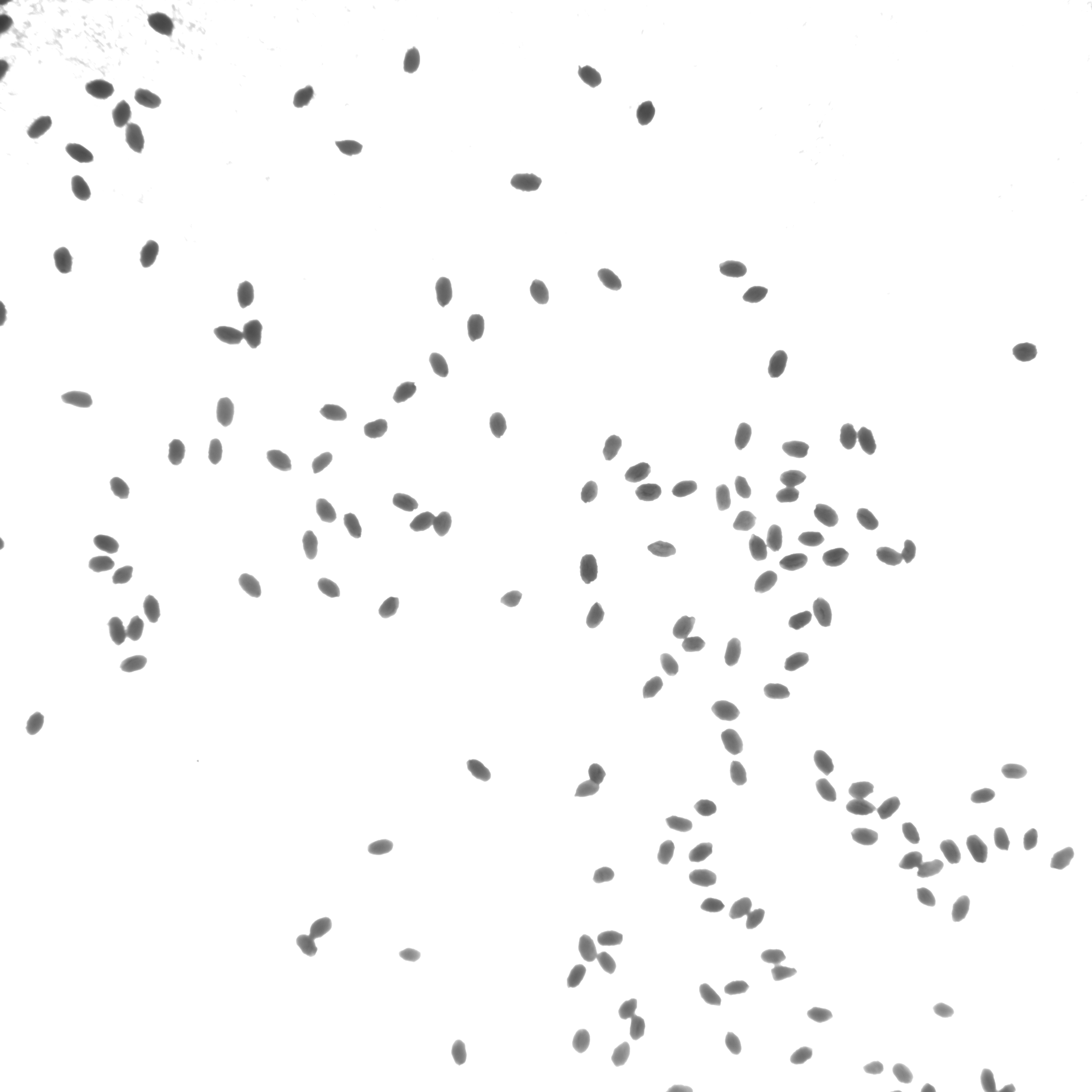

Supplement: Supplementary file 1 — Supplementary Material 1 [file 13007_2025_1406_MOESM1_ESM.zip › performance_comparison_images/VZ314-14_BF.tif]

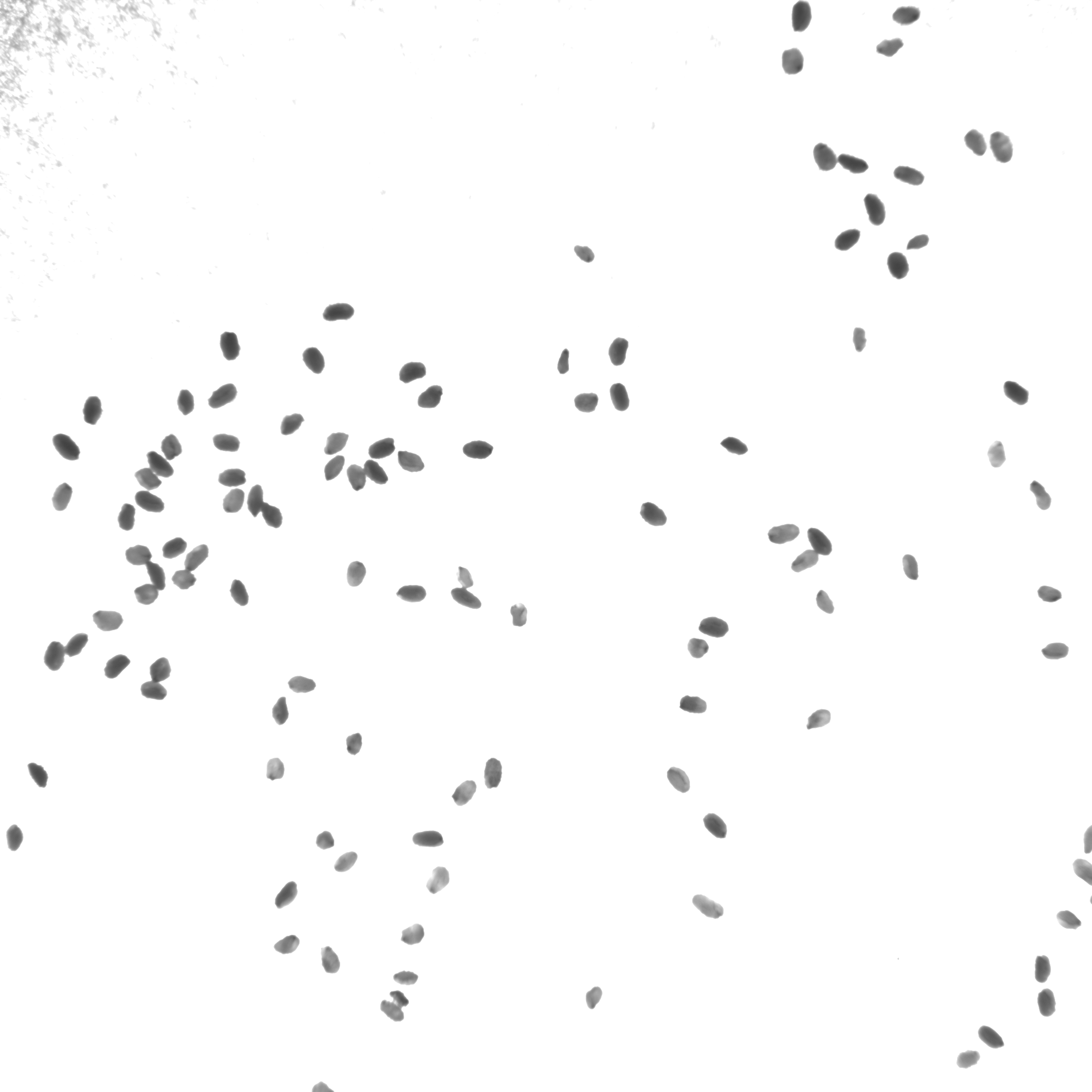

Supplement: Supplementary file 1 — Supplementary Material 1 [file 13007_2025_1406_MOESM1_ESM.zip › performance_comparison_images/VZ312-9_BF.tif]

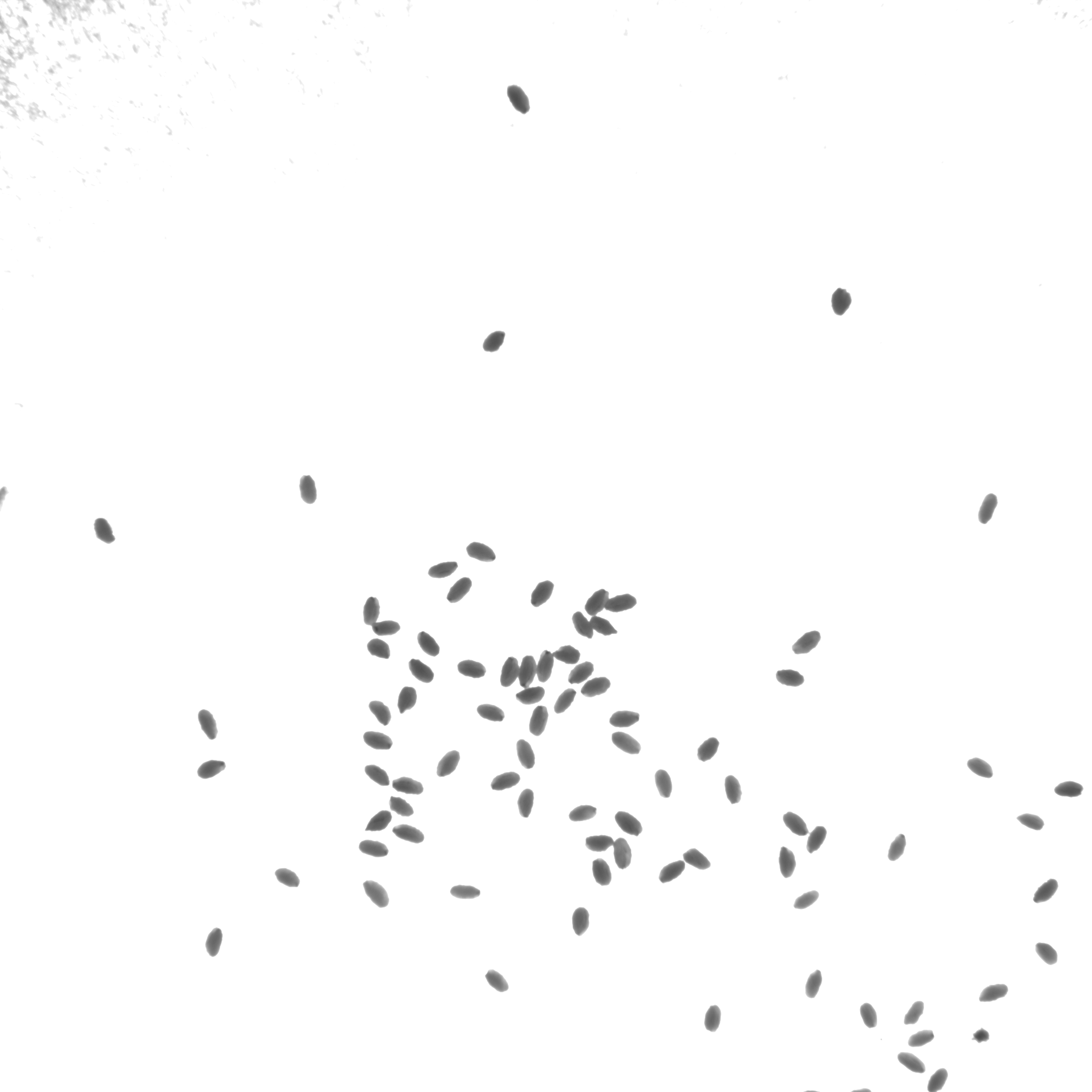

Supplement: Supplementary file 1 — Supplementary Material 1 [file 13007_2025_1406_MOESM1_ESM.zip › performance_comparison_images/VZ313-9_BF.tif]

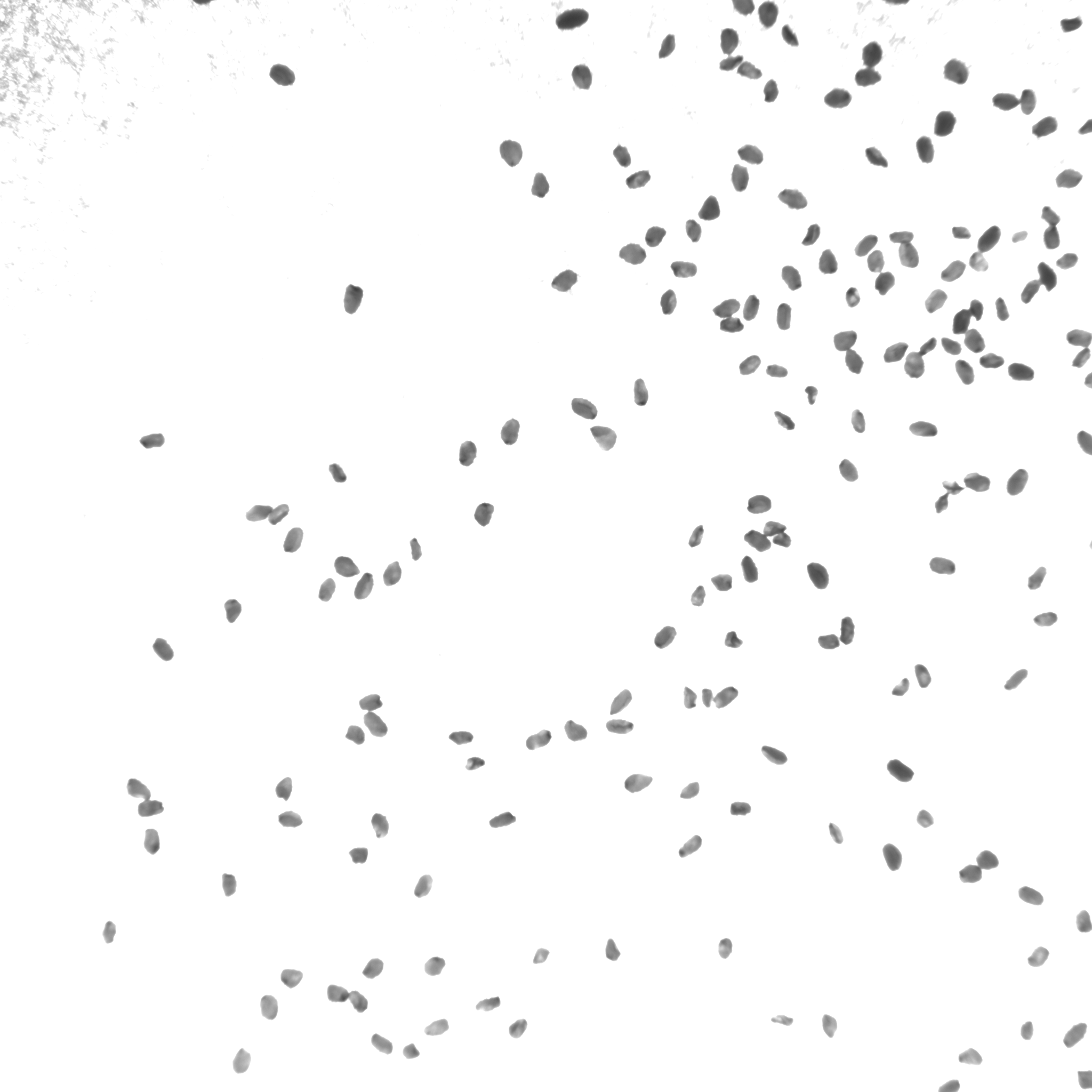

Supplement: Supplementary file 1 — Supplementary Material 1 [file 13007_2025_1406_MOESM1_ESM.zip › performance_comparison_images/VZ312-5_BF.tif]

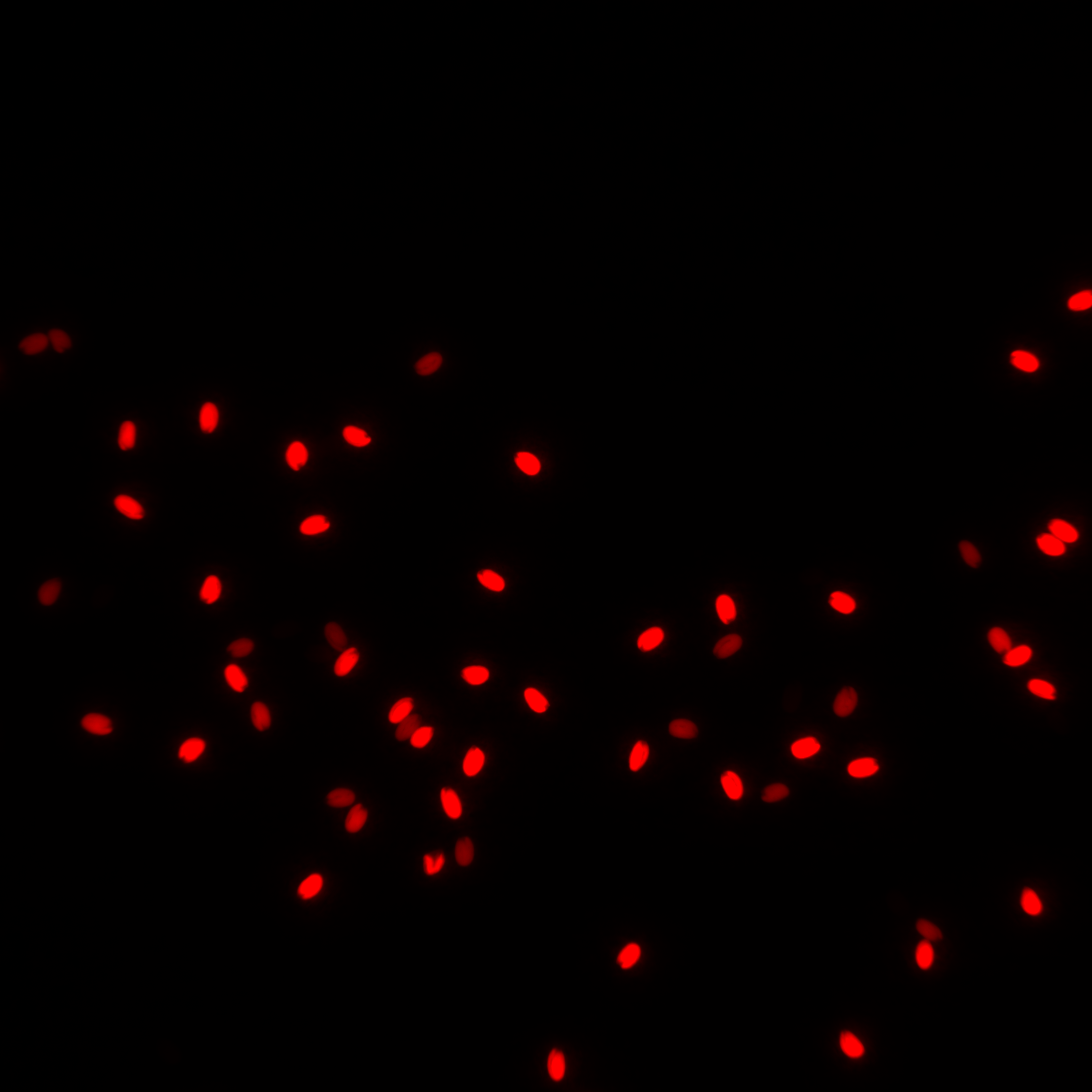

Supplement: Supplementary file 1 — Supplementary Material 1 [file 13007_2025_1406_MOESM1_ESM.zip › performance_comparison_images/VZ314-12_FL.tif]

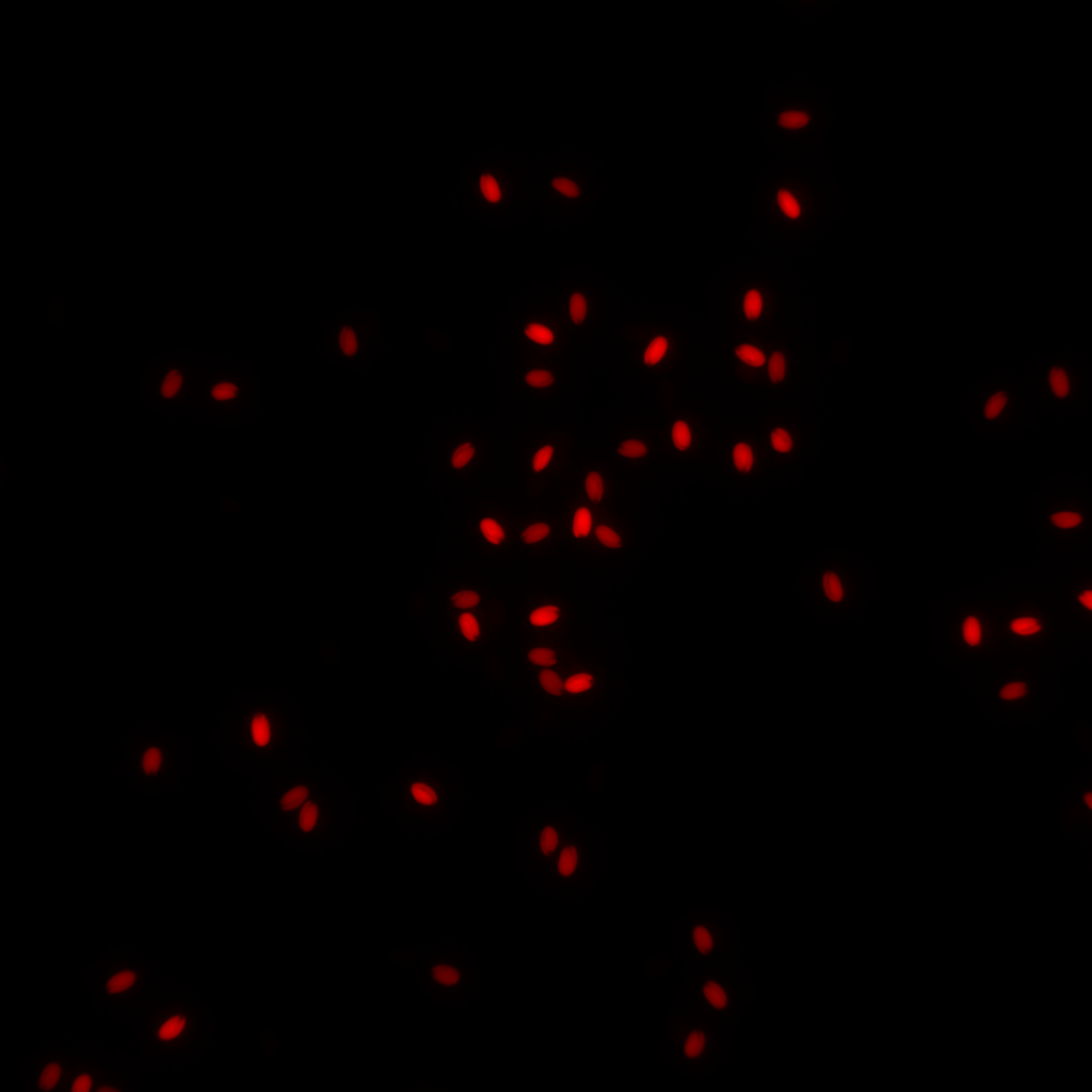

Supplement: Supplementary file 1 — Supplementary Material 1 [file 13007_2025_1406_MOESM1_ESM.zip › performance_comparison_images/VZ313-3_FL.tif]

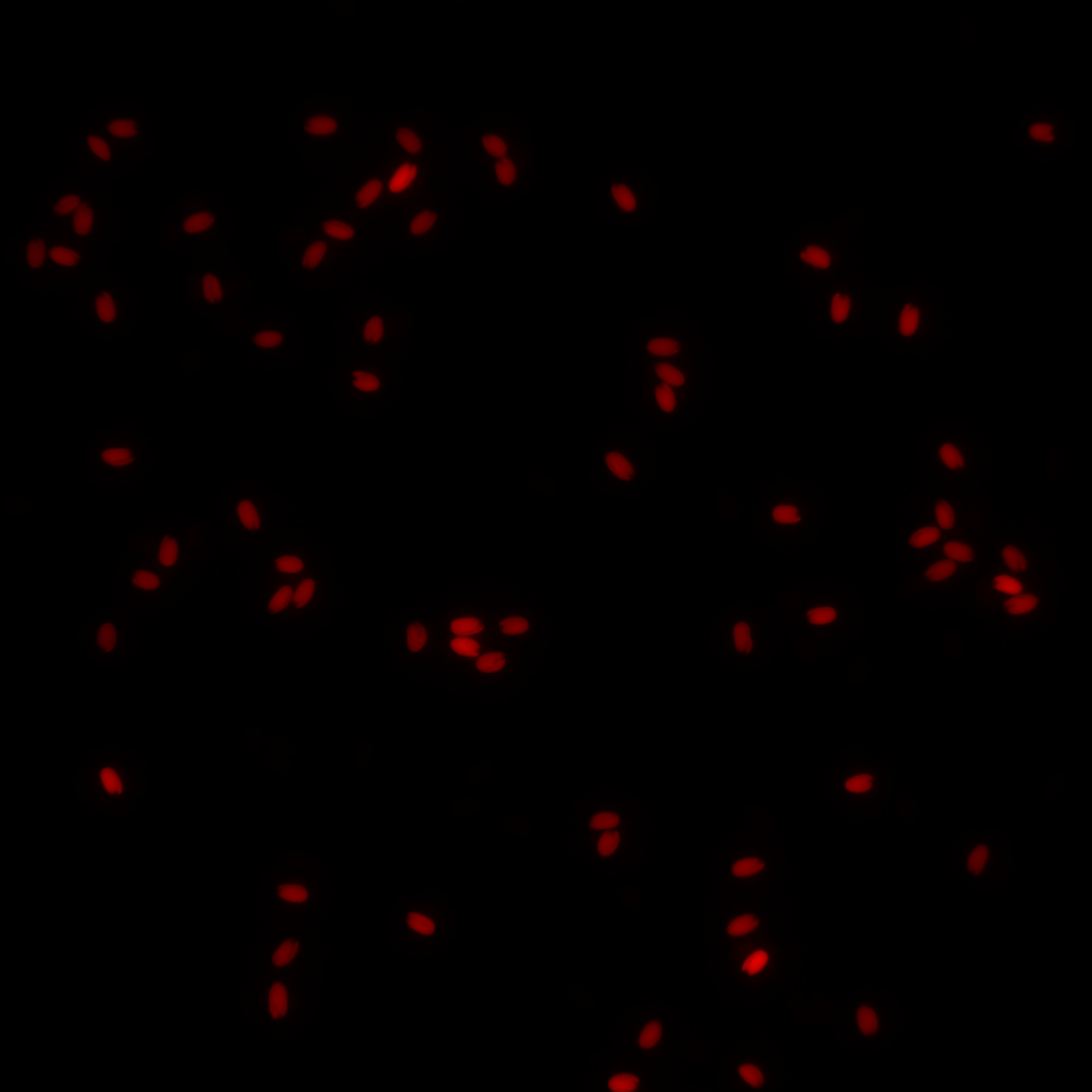

Supplement: Supplementary file 1 — Supplementary Material 1 [file 13007_2025_1406_MOESM1_ESM.zip › performance_comparison_images/VZ314-10_FL.tif]

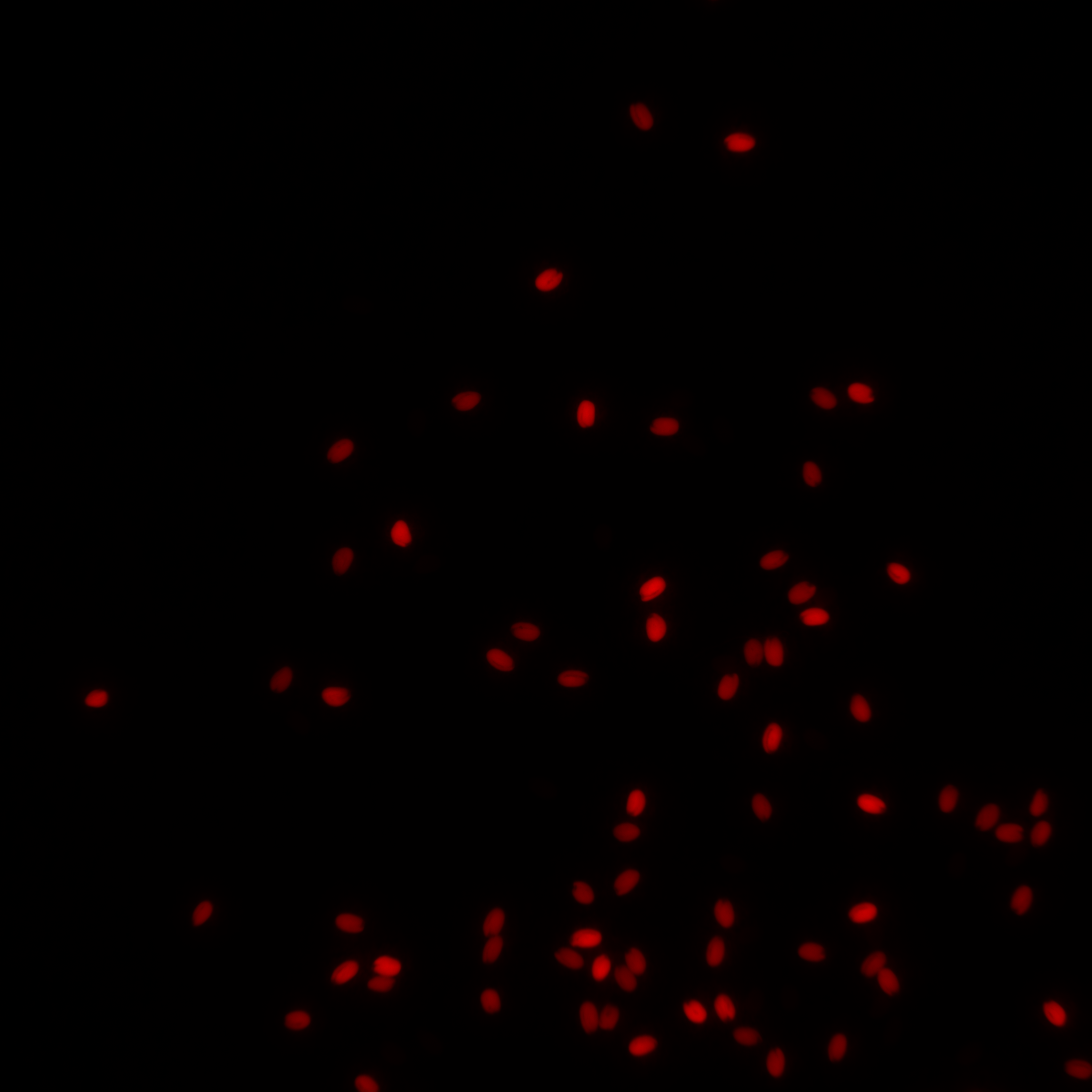

Supplement: Supplementary file 1 — Supplementary Material 1 [file 13007_2025_1406_MOESM1_ESM.zip › performance_comparison_images/VZ314-5_FL.tif]

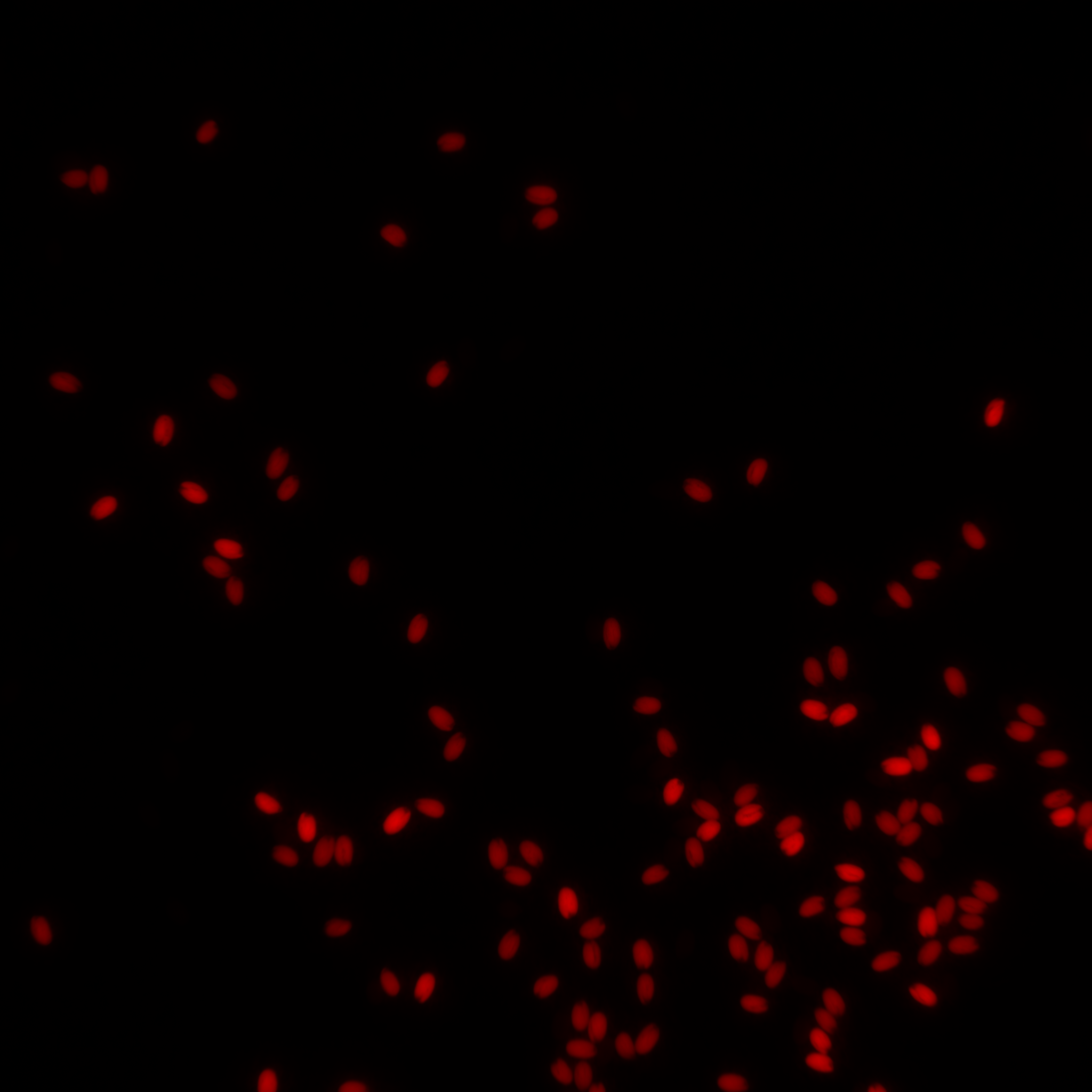

Supplement: Supplementary file 1 — Supplementary Material 1 [file 13007_2025_1406_MOESM1_ESM.zip › performance_comparison_images/VZ314-9_FL.tif]

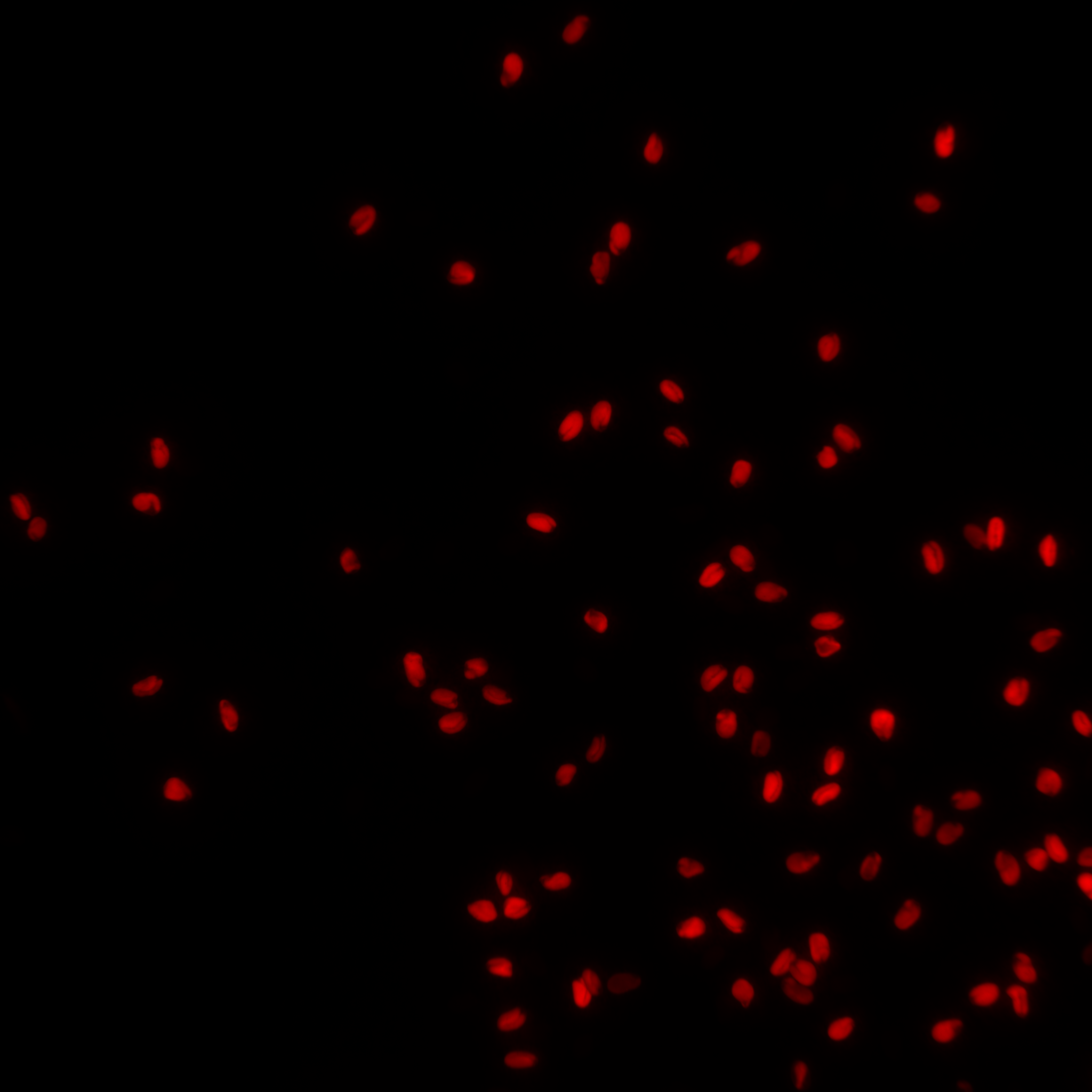

Supplement: Supplementary file 1 — Supplementary Material 1 [file 13007_2025_1406_MOESM1_ESM.zip › performance_comparison_images/VZ312-1_FL.tif]

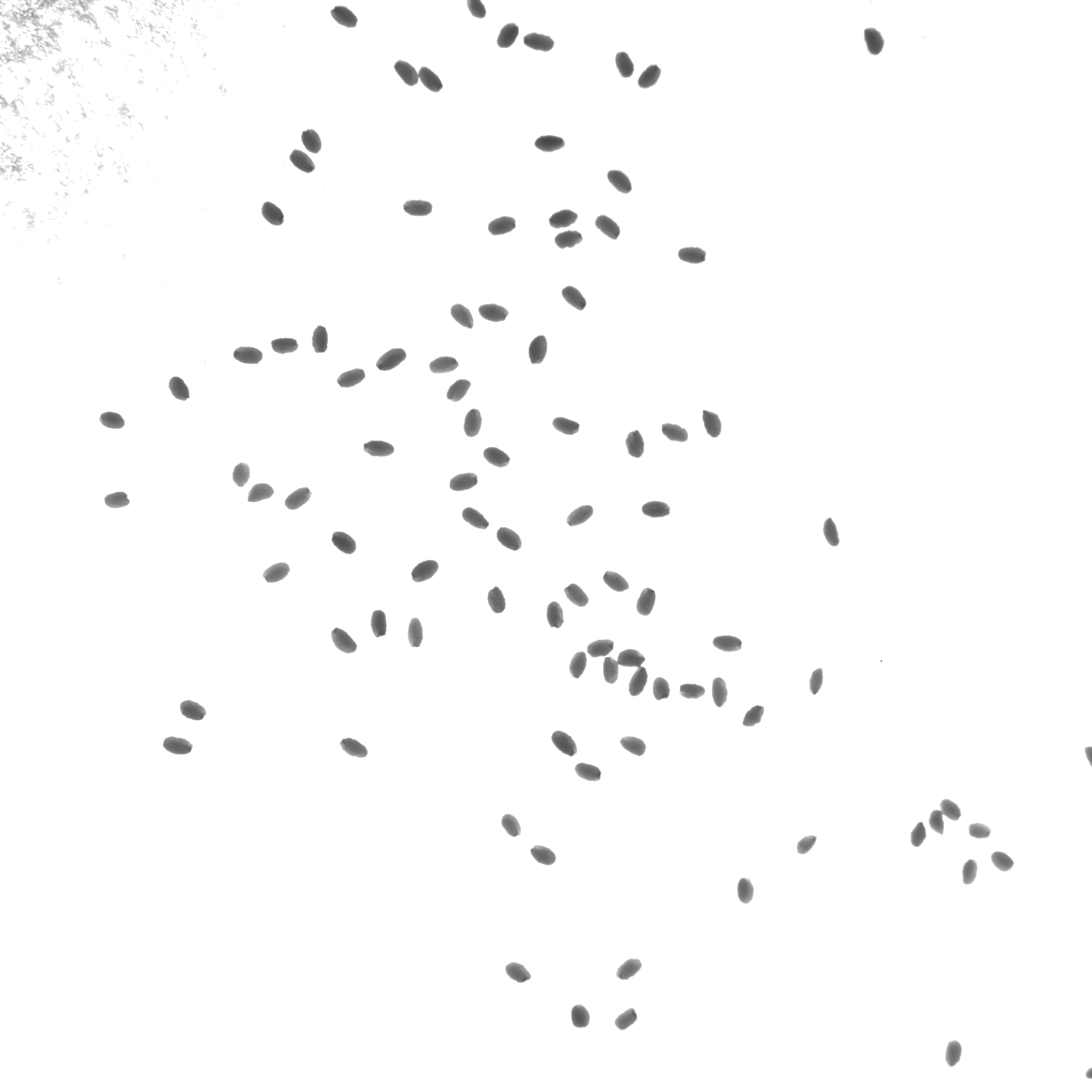

Supplement: Supplementary file 1 — Supplementary Material 1 [file 13007_2025_1406_MOESM1_ESM.zip › performance_comparison_images/VZ314-16_BF.tif]

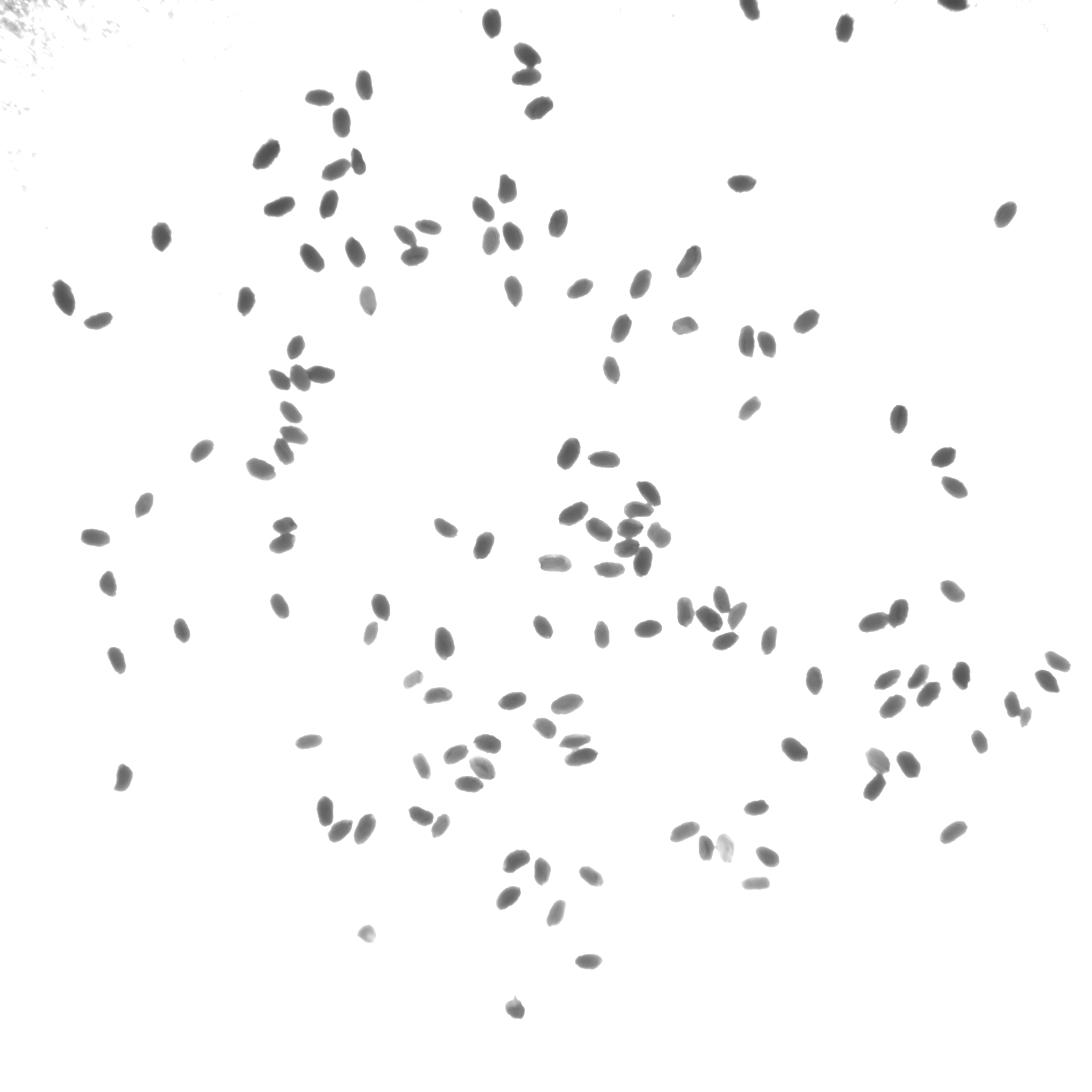

Supplement: Supplementary file 1 — Supplementary Material 1 [file 13007_2025_1406_MOESM1_ESM.zip › performance_comparison_images/VZ314-3_BF.tif]

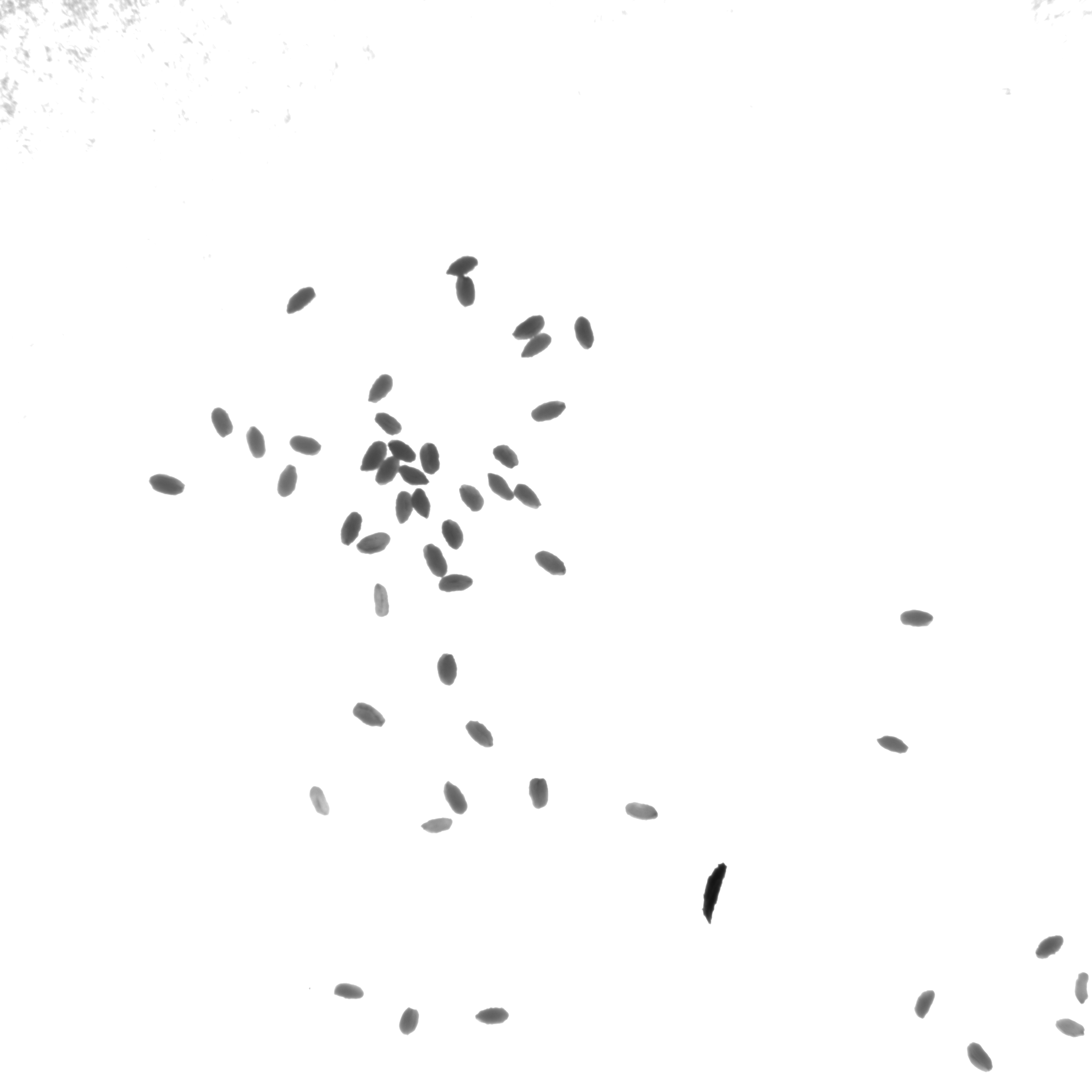

Supplement: Supplementary file 1 — Supplementary Material 1 [file 13007_2025_1406_MOESM1_ESM.zip › performance_comparison_images/VZ313-7_BF.tif]

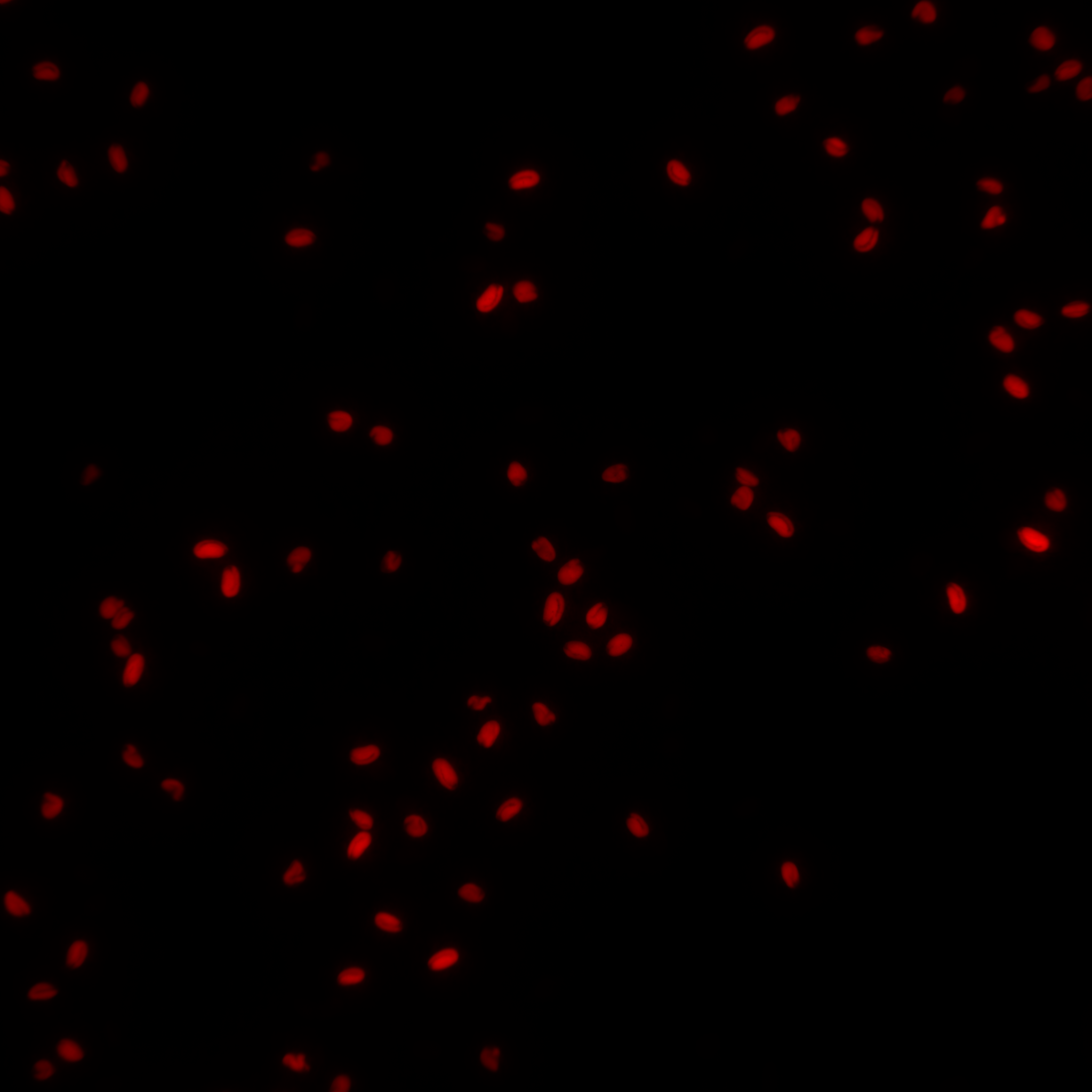

Supplement: Supplementary file 1 — Supplementary Material 1 [file 13007_2025_1406_MOESM1_ESM.zip › performance_comparison_images/VZ312-12_FL.tif]

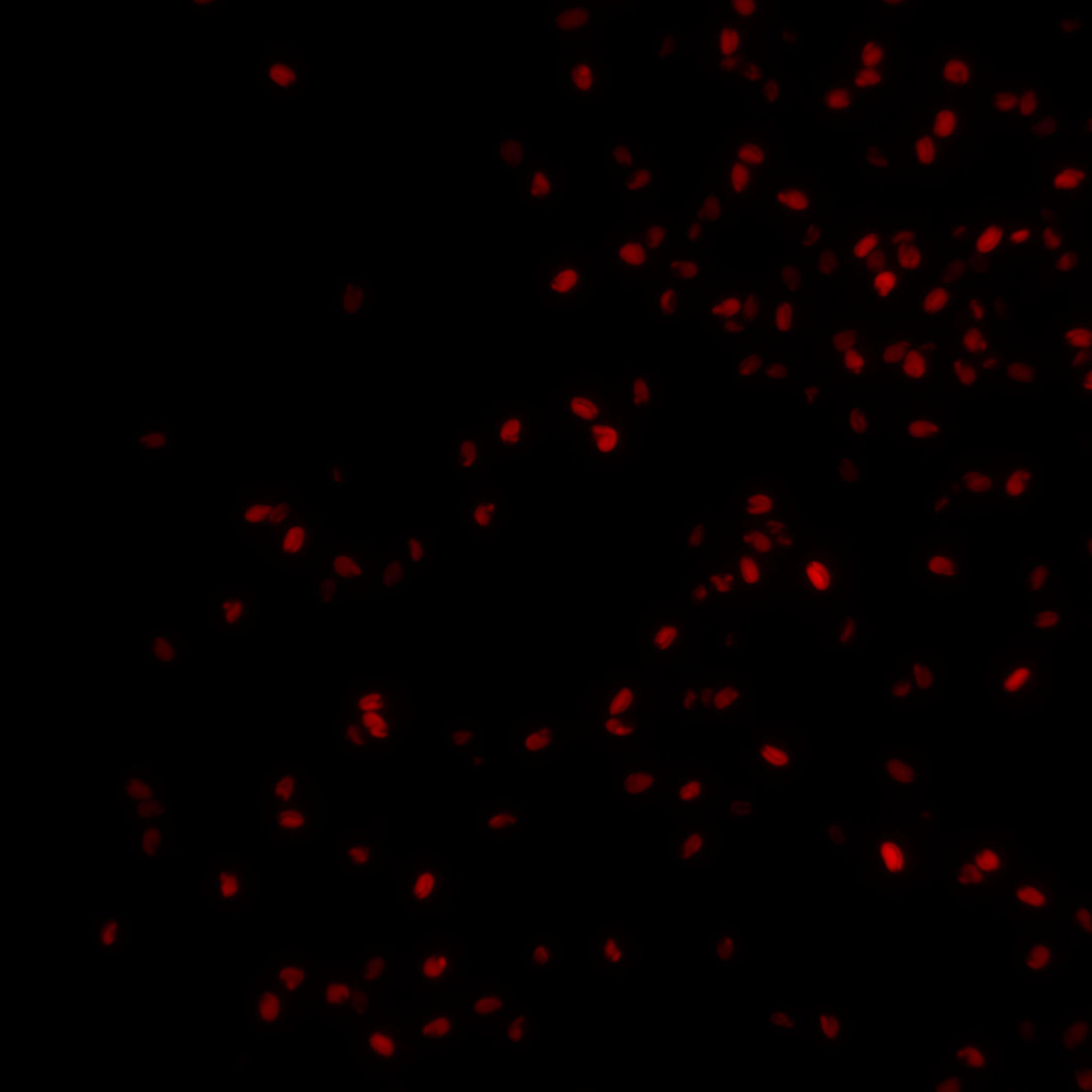

Supplement: Supplementary file 1 — Supplementary Material 1 [file 13007_2025_1406_MOESM1_ESM.zip › performance_comparison_images/VZ312-5_FL.tif]

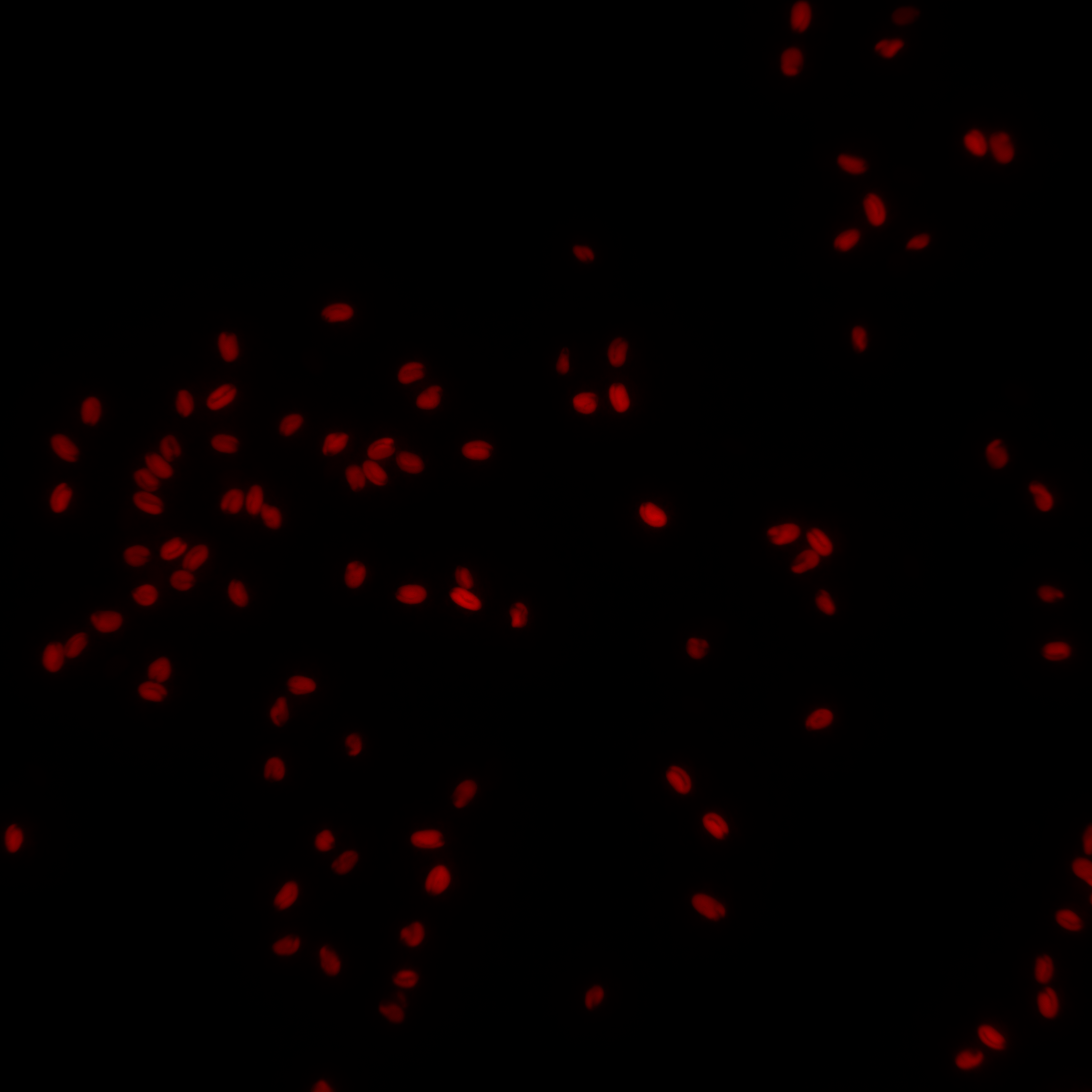

Supplement: Supplementary file 1 — Supplementary Material 1 [file 13007_2025_1406_MOESM1_ESM.zip › performance_comparison_images/VZ312-9_FL.tif]

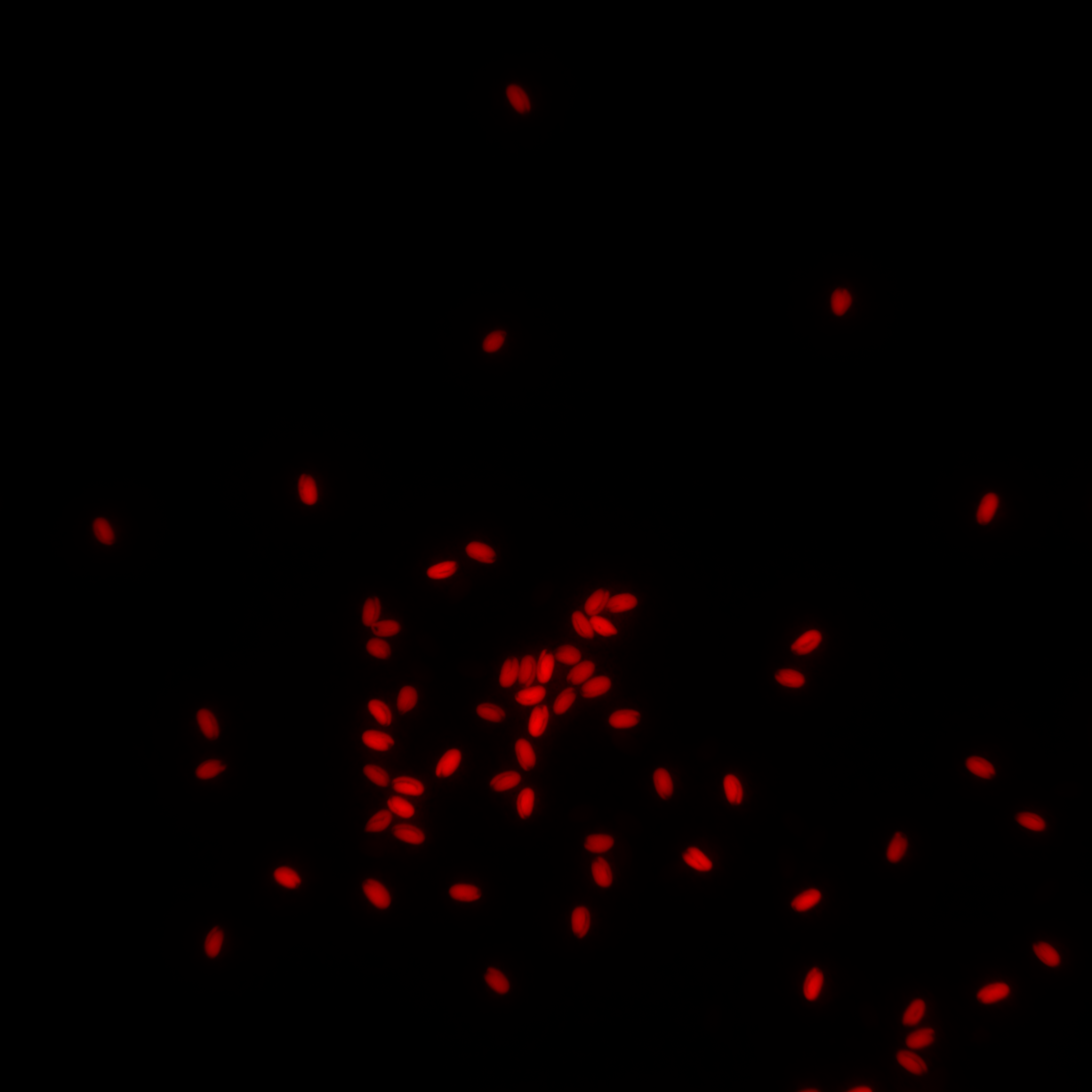

Supplement: Supplementary file 1 — Supplementary Material 1 [file 13007_2025_1406_MOESM1_ESM.zip › performance_comparison_images/VZ313-9_FL.tif]

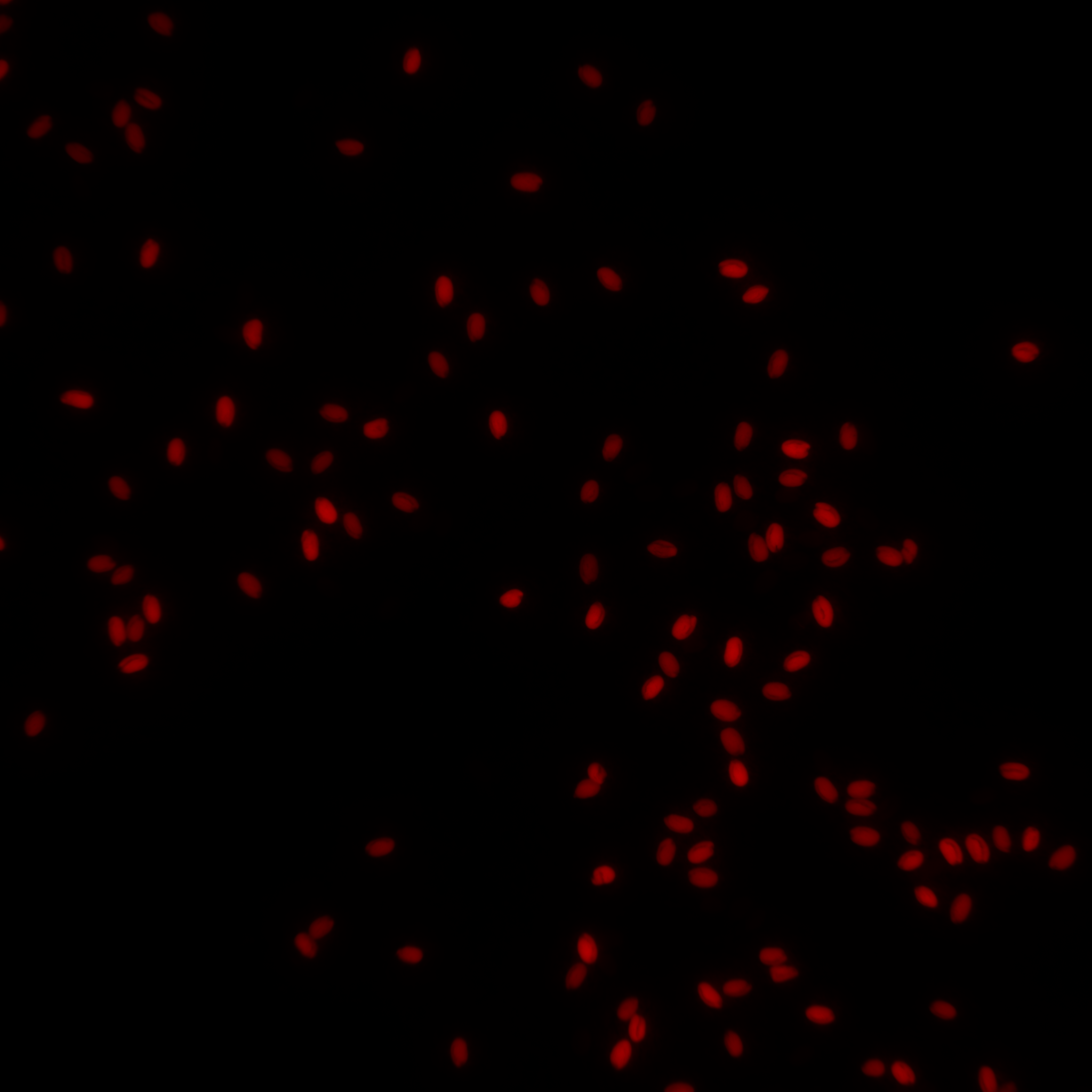

Supplement: Supplementary file 1 — Supplementary Material 1 [file 13007_2025_1406_MOESM1_ESM.zip › performance_comparison_images/VZ314-14_FL.tif]

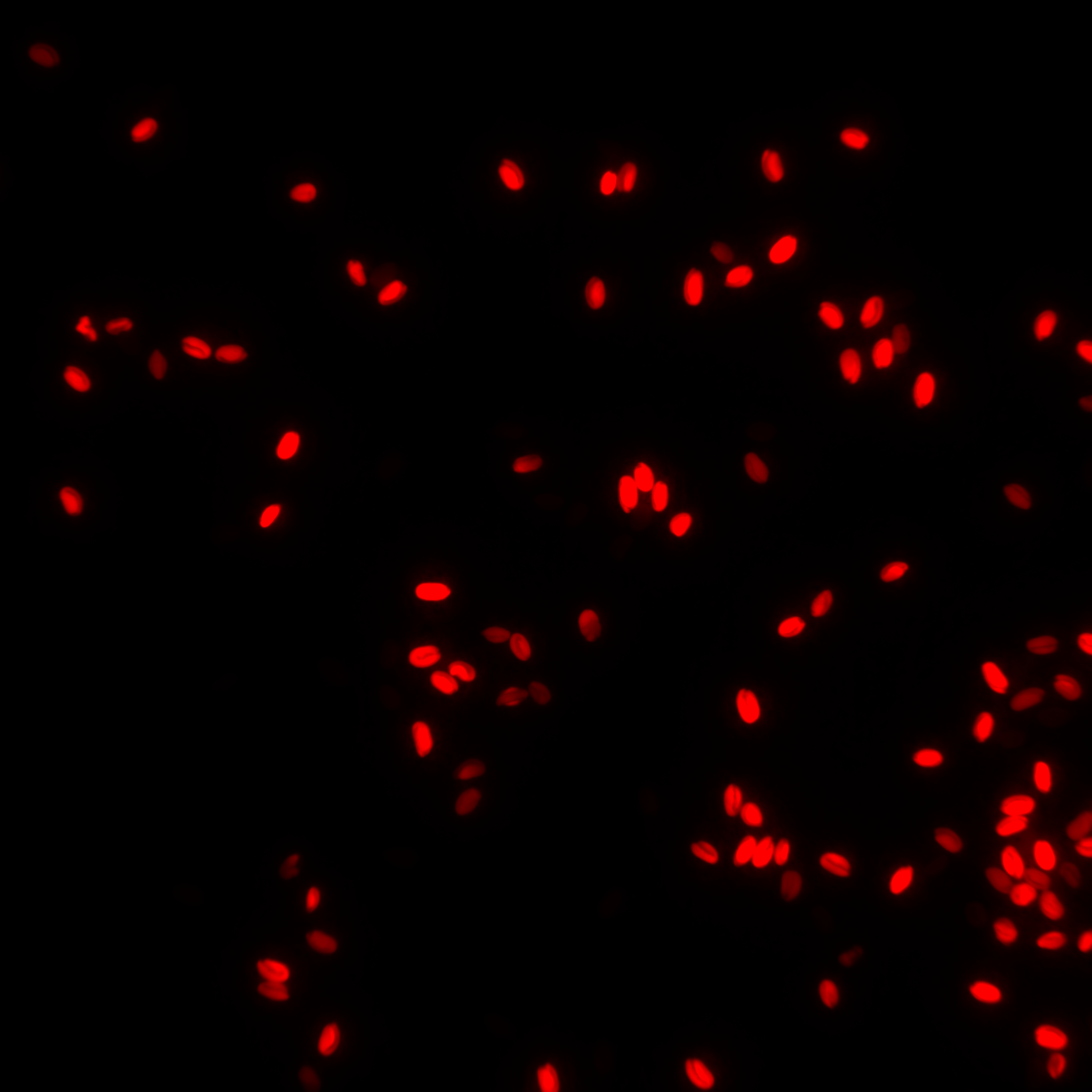

Supplement: Supplementary file 1 — Supplementary Material 1 [file 13007_2025_1406_MOESM1_ESM.zip › performance_comparison_images/VZ314-1_FL.tif]

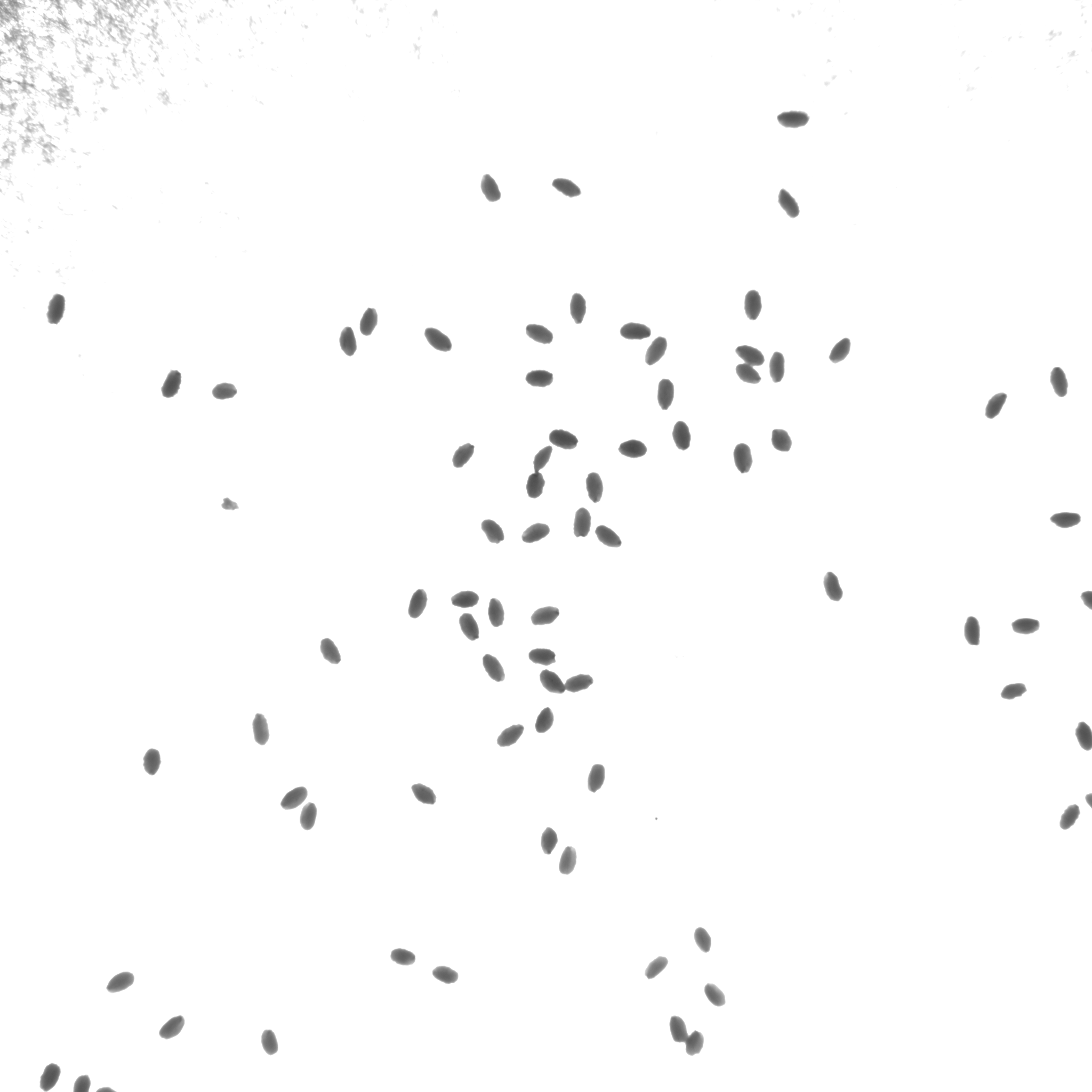

Supplement: Supplementary file 1 — Supplementary Material 1 [file 13007_2025_1406_MOESM1_ESM.zip › performance_comparison_images/VZ313-3_BF.tif]

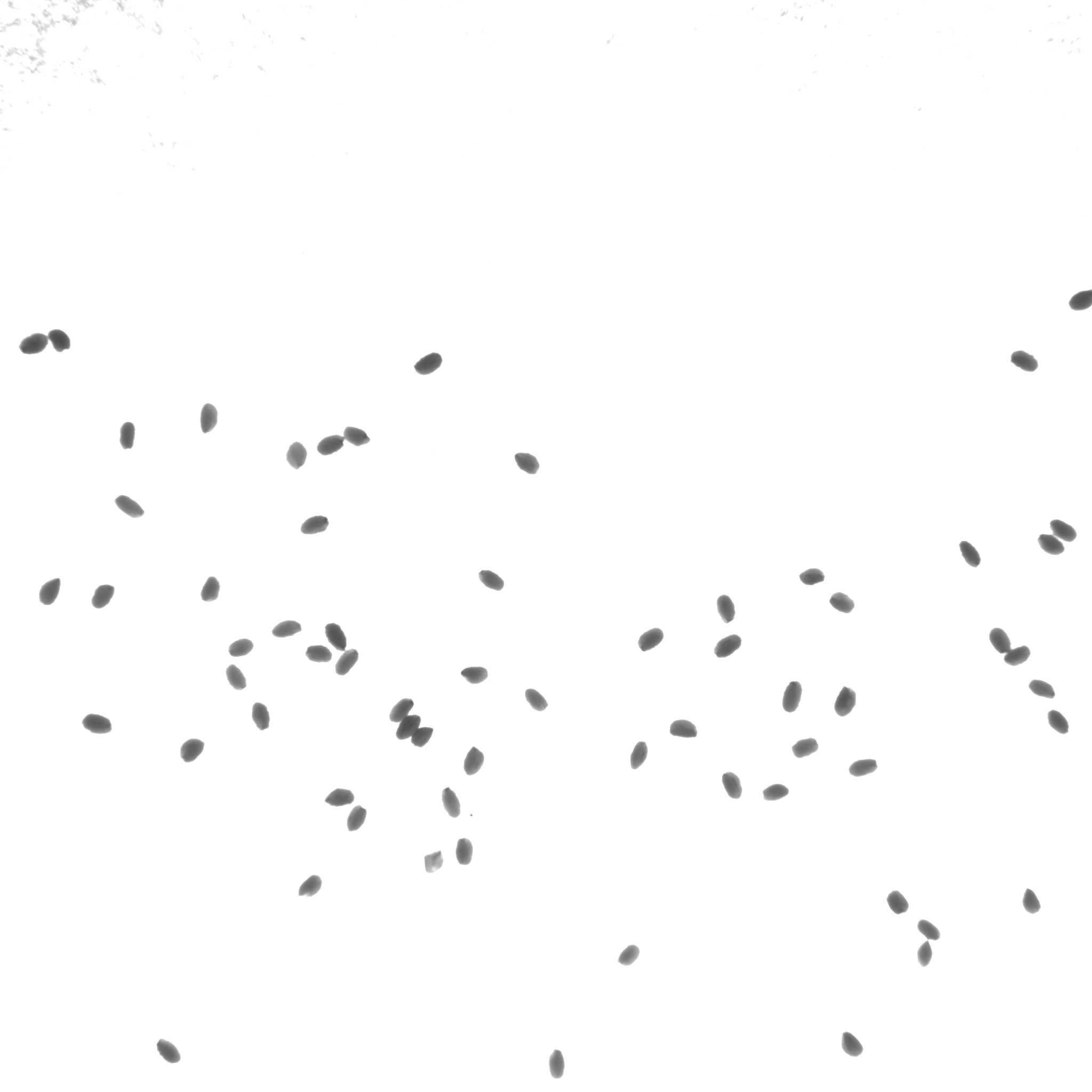

Supplement: Supplementary file 1 — Supplementary Material 1 [file 13007_2025_1406_MOESM1_ESM.zip › performance_comparison_images/VZ314-12_BF.tif]

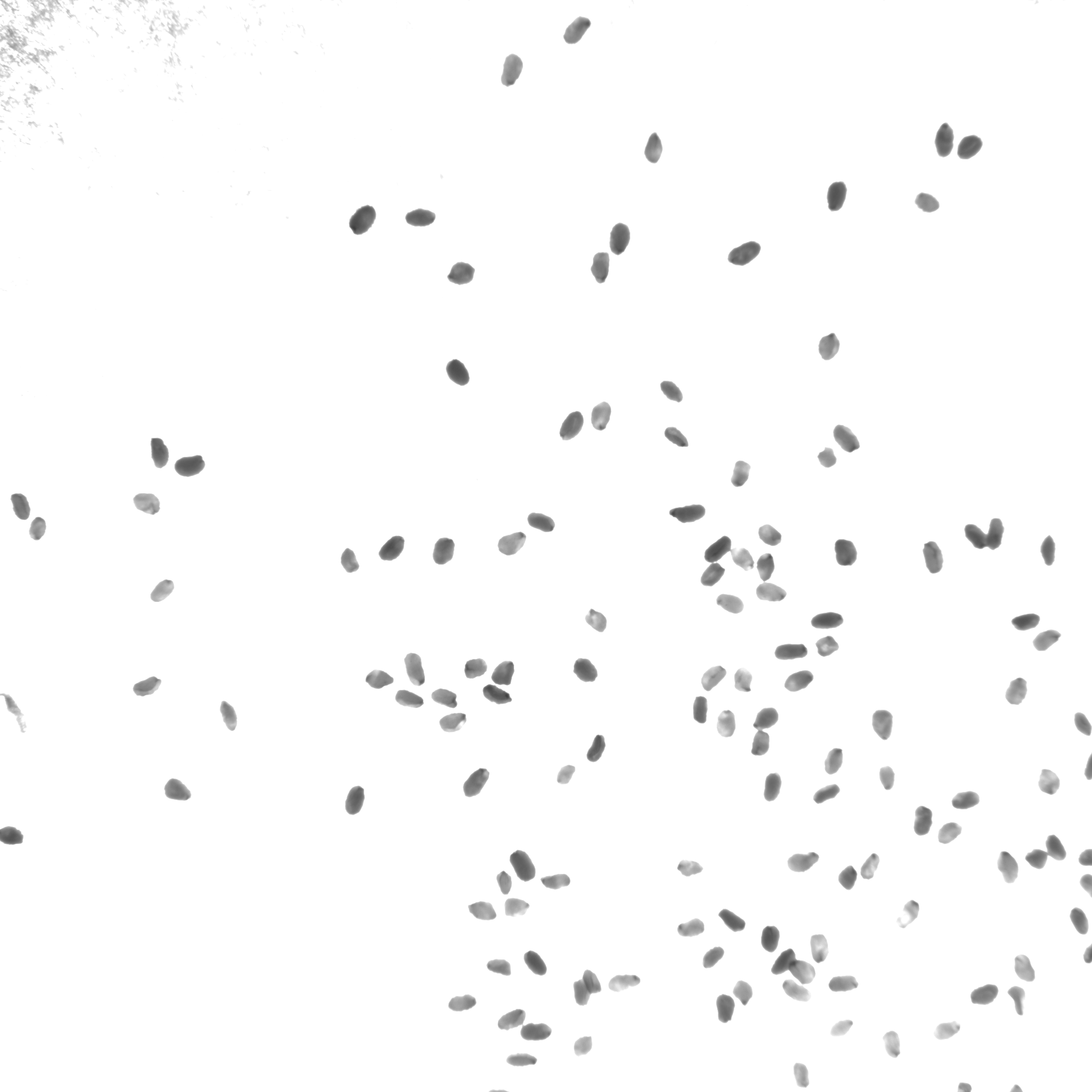

Supplement: Supplementary file 1 — Supplementary Material 1 [file 13007_2025_1406_MOESM1_ESM.zip › performance_comparison_images/VZ312-1_BF.tif]

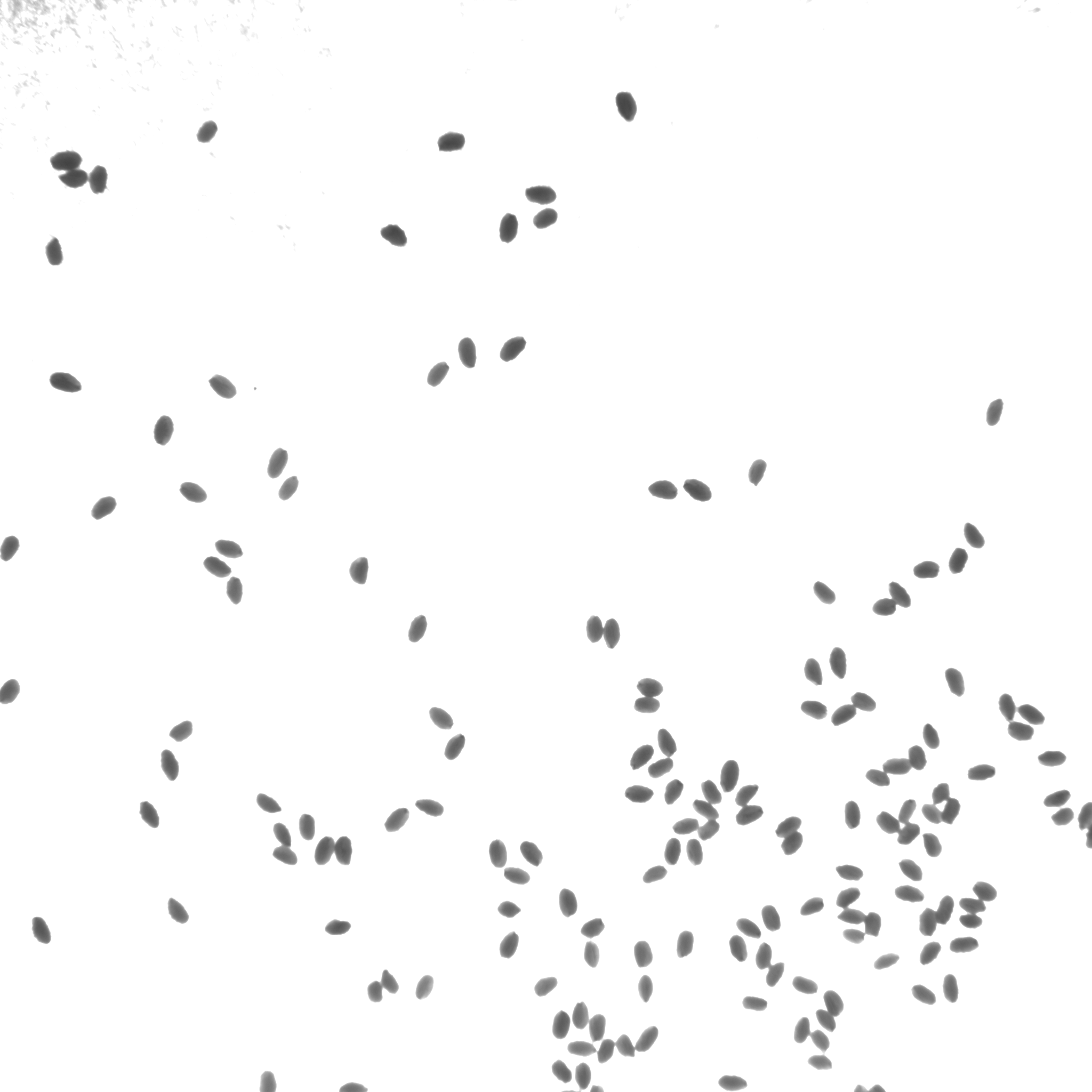

Supplement: Supplementary file 1 — Supplementary Material 1 [file 13007_2025_1406_MOESM1_ESM.zip › performance_comparison_images/VZ314-9_BF.tif]

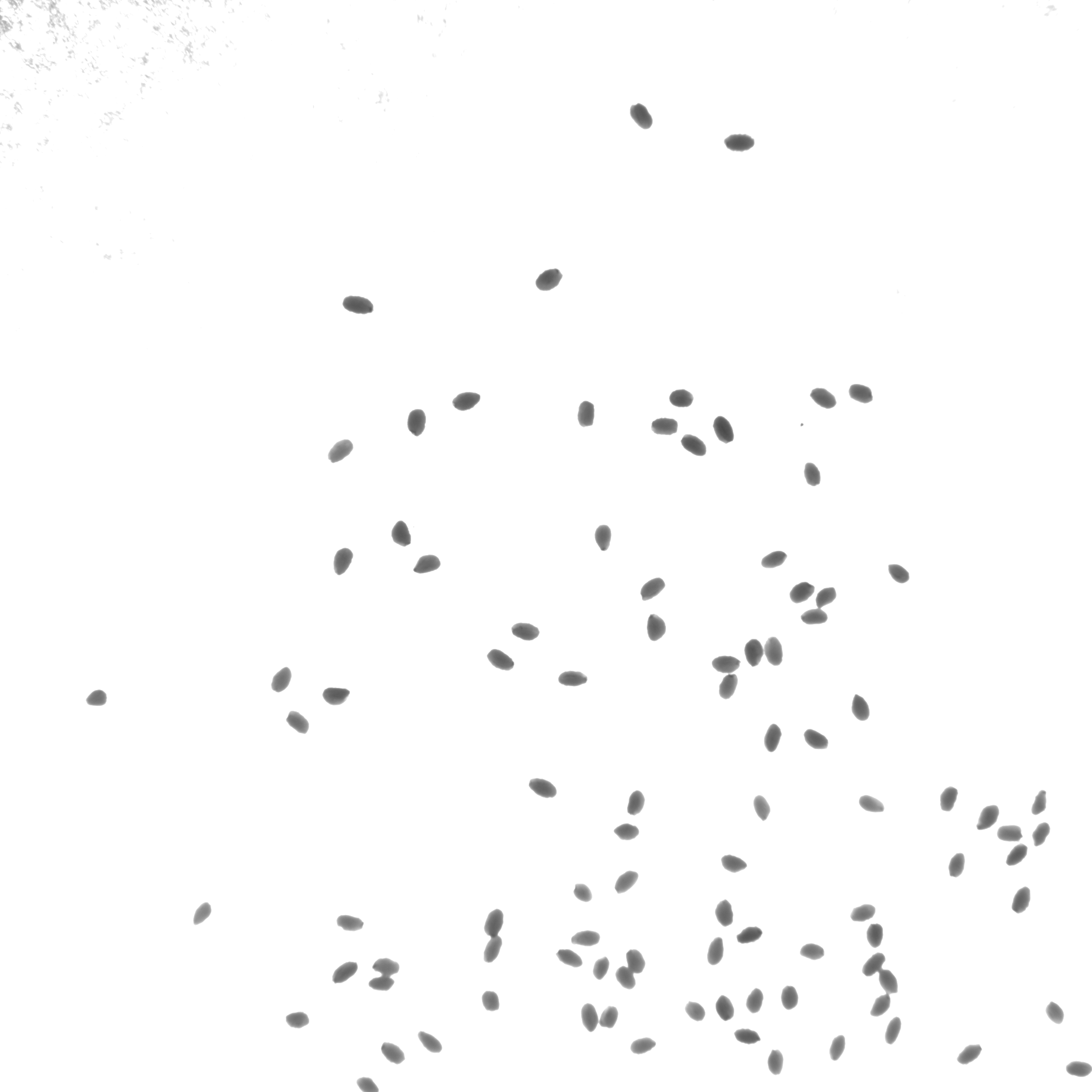

Supplement: Supplementary file 1 — Supplementary Material 1 [file 13007_2025_1406_MOESM1_ESM.zip › performance_comparison_images/VZ314-5_BF.tif]

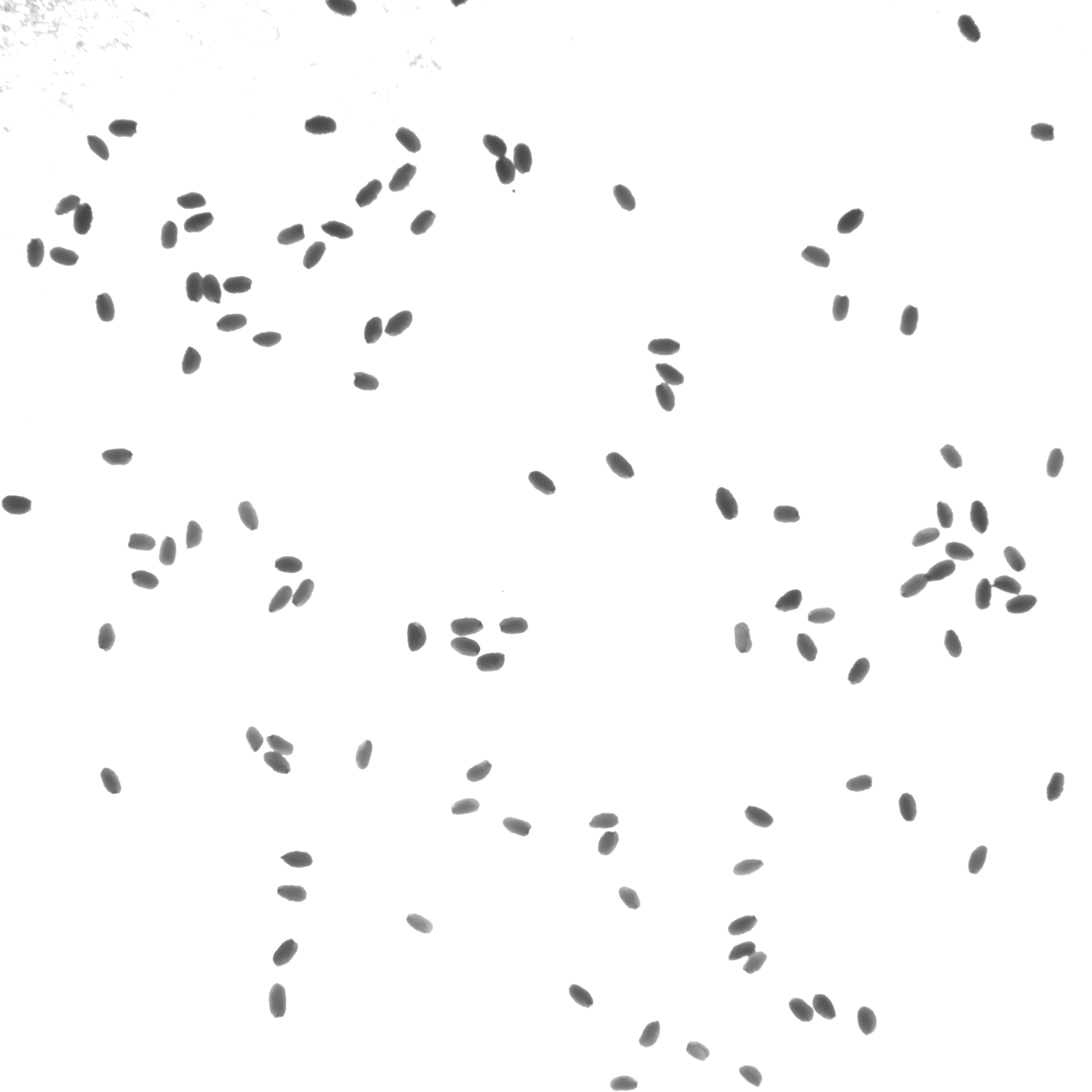

Supplement: Supplementary file 1 — Supplementary Material 1 [file 13007_2025_1406_MOESM1_ESM.zip › performance_comparison_images/VZ314-10_BF.tif]

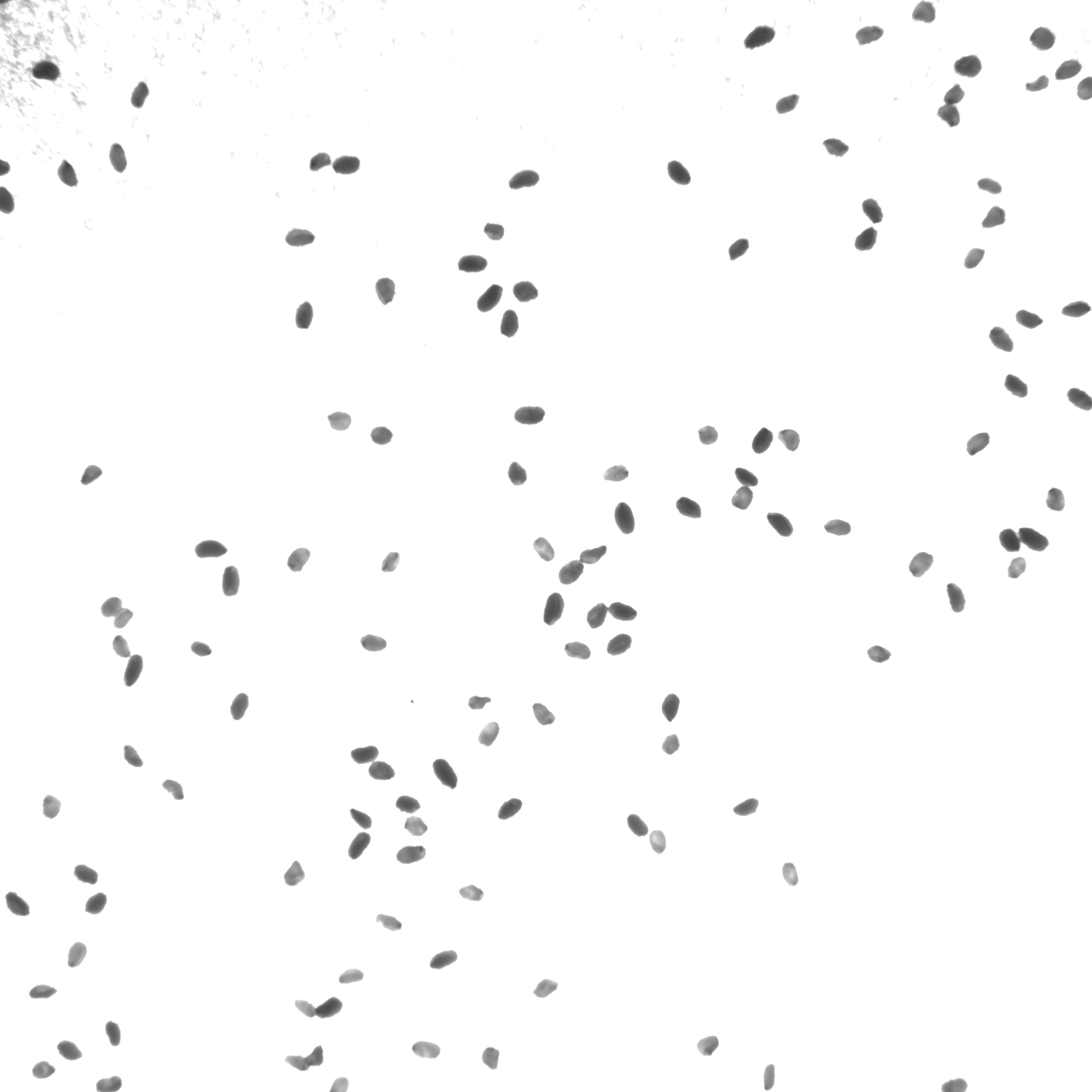

Supplement: Supplementary file 1 — Supplementary Material 1 [file 13007_2025_1406_MOESM1_ESM.zip › performance_comparison_images/VZ312-12_BF.tif]

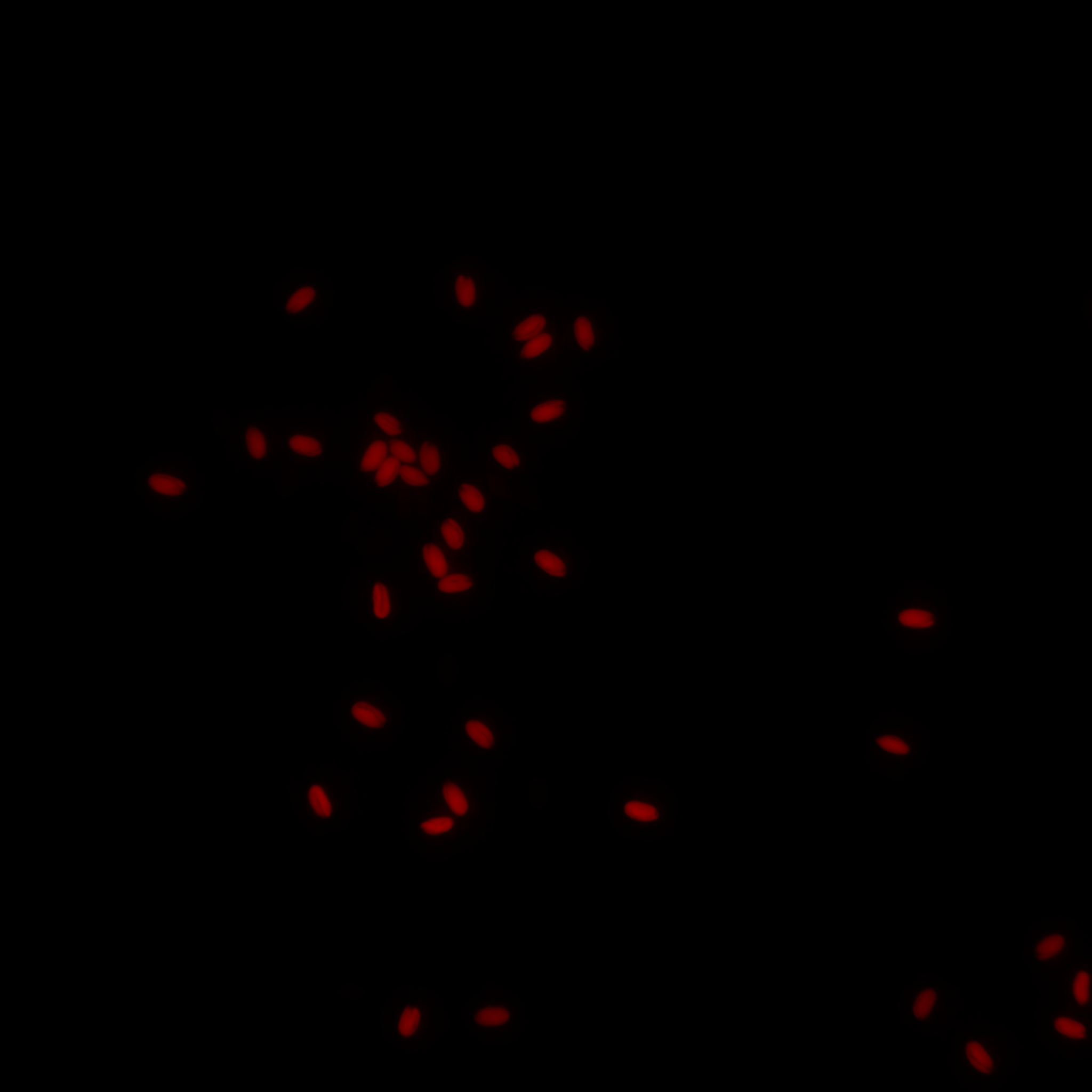

Supplement: Supplementary file 1 — Supplementary Material 1 [file 13007_2025_1406_MOESM1_ESM.zip › performance_comparison_images/VZ313-7_FL.tif]

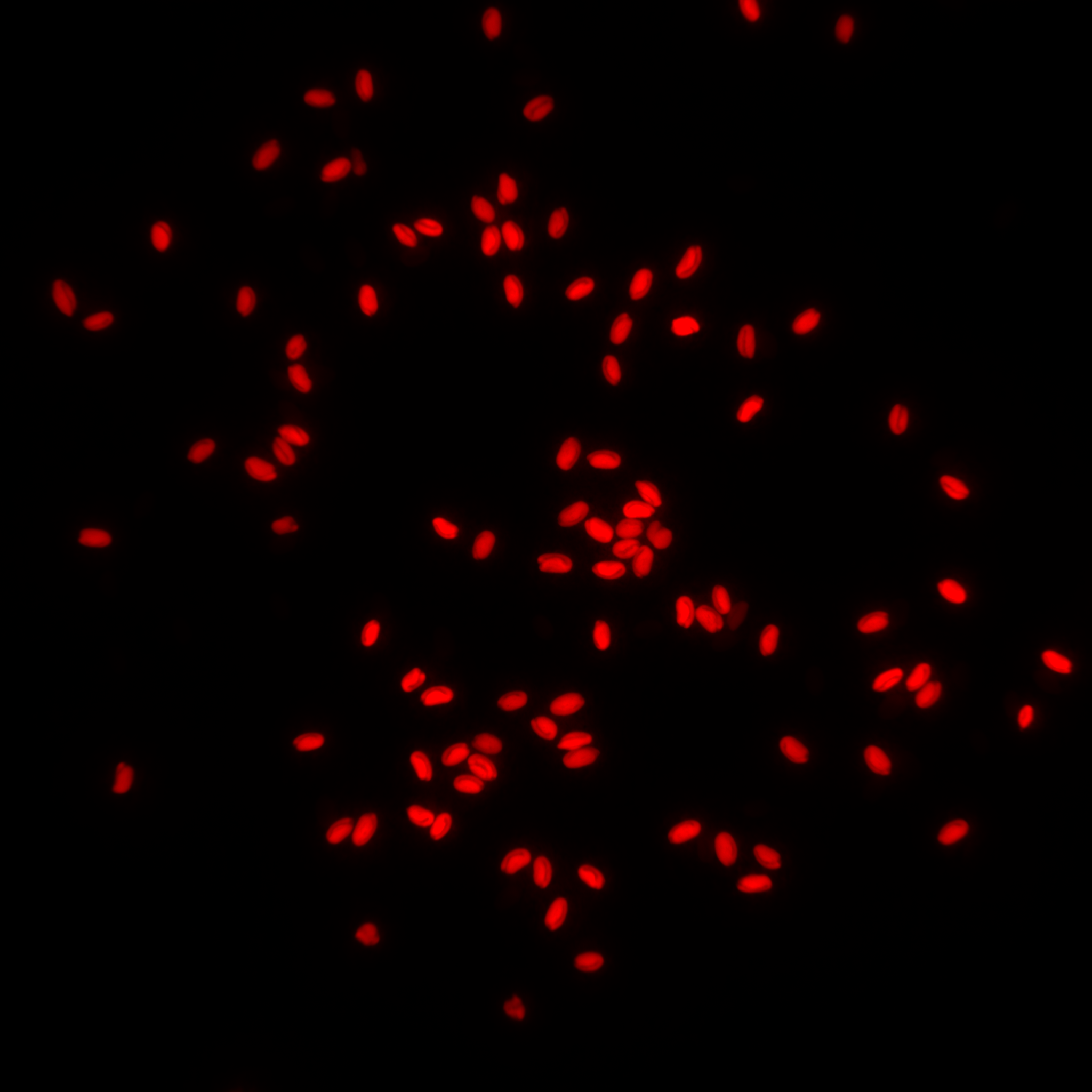

Supplement: Supplementary file 1 — Supplementary Material 1 [file 13007_2025_1406_MOESM1_ESM.zip › performance_comparison_images/VZ314-3_FL.tif]

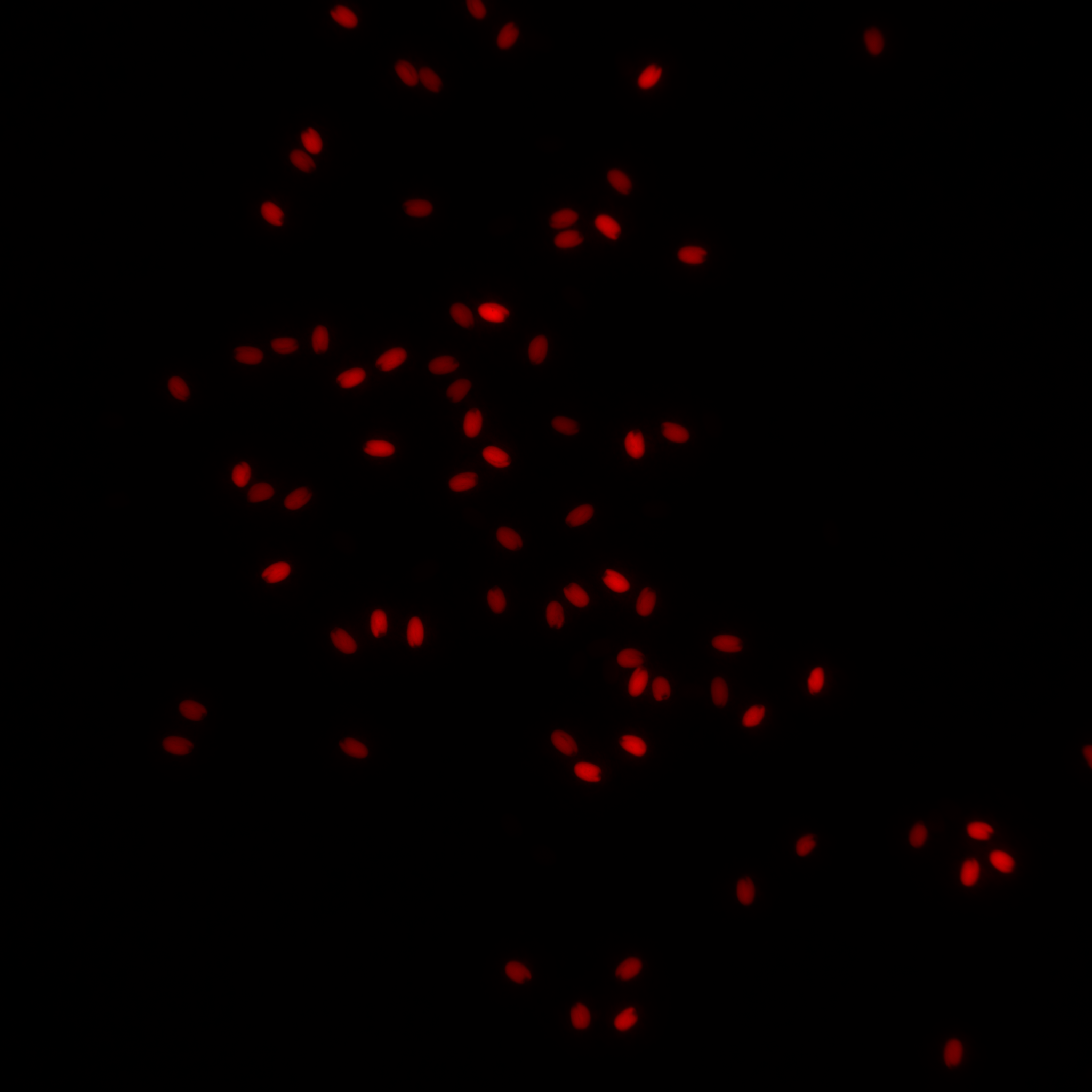

Supplement: Supplementary file 1 — Supplementary Material 1 [file 13007_2025_1406_MOESM1_ESM.zip › performance_comparison_images/VZ314-16_FL.tif]

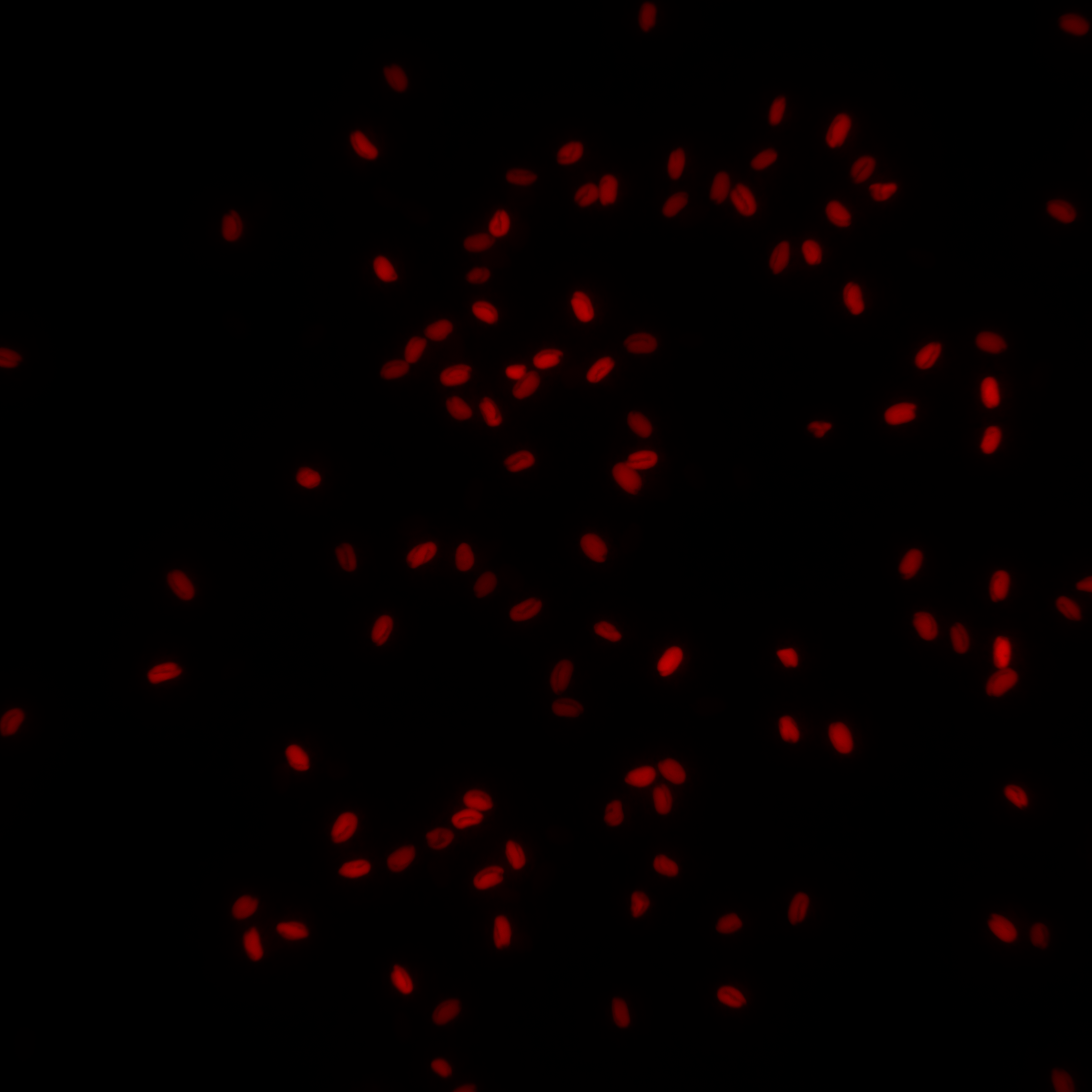

Supplement: Supplementary file 1 — Supplementary Material 1 [file 13007_2025_1406_MOESM1_ESM.zip › performance_comparison_images/VZ312-2_FL.tif]

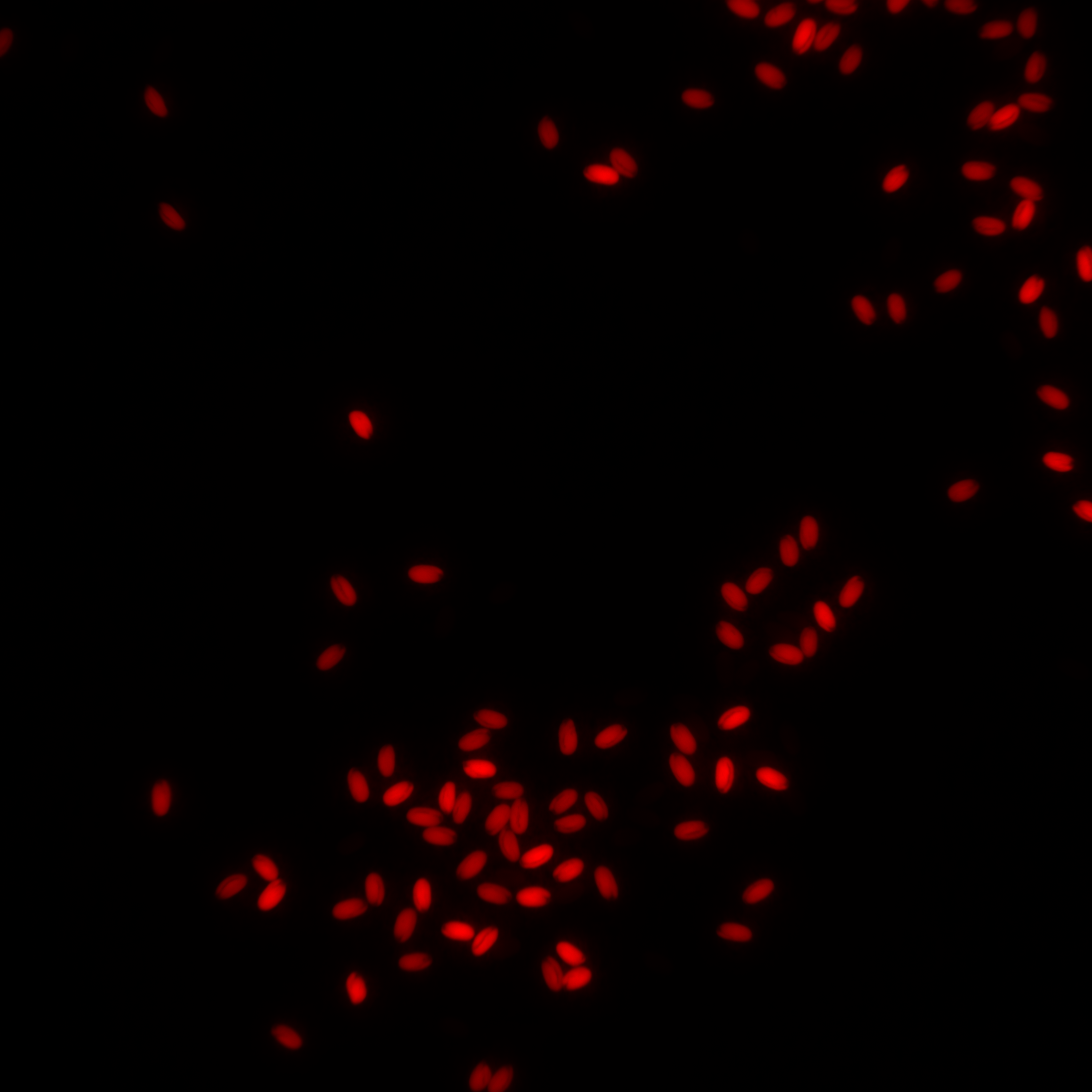

Supplement: Supplementary file 1 — Supplementary Material 1 [file 13007_2025_1406_MOESM1_ESM.zip › performance_comparison_images/VZ313-2_FL.tif]

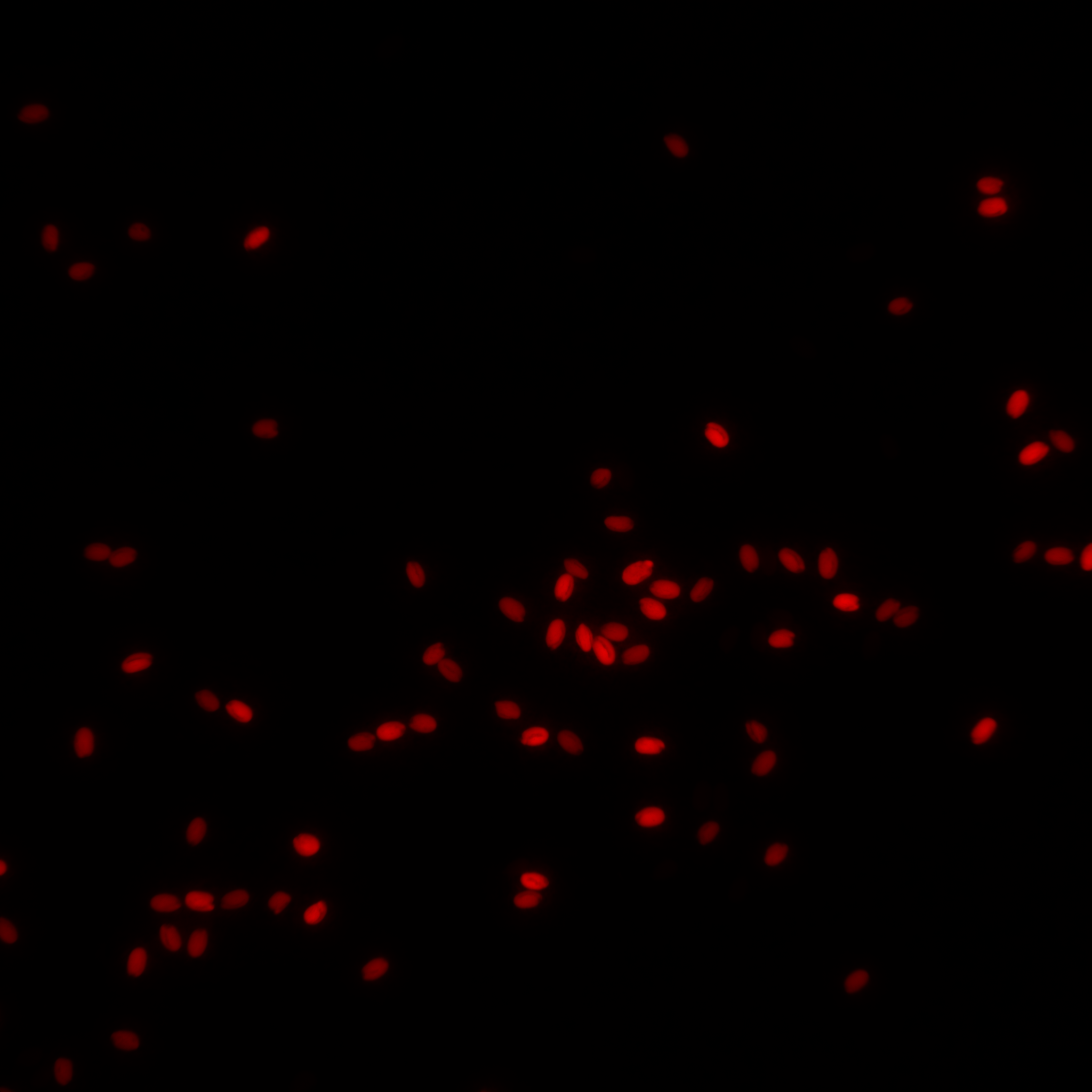

Supplement: Supplementary file 1 — Supplementary Material 1 [file 13007_2025_1406_MOESM1_ESM.zip › performance_comparison_images/VZ314-13_FL.tif]

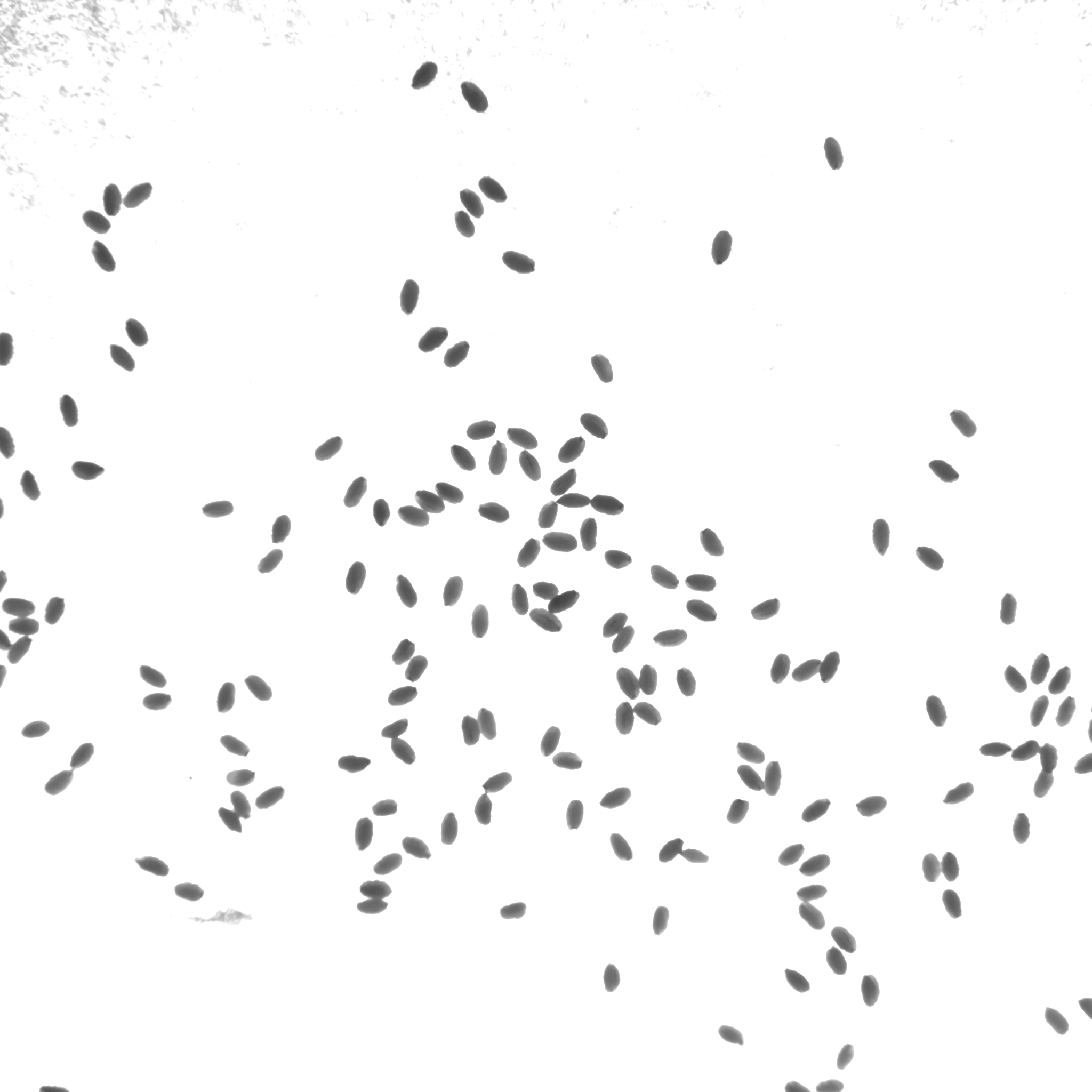

Supplement: Supplementary file 1 — Supplementary Material 1 [file 13007_2025_1406_MOESM1_ESM.zip › performance_comparison_images/VZ313-8_BF.tif]

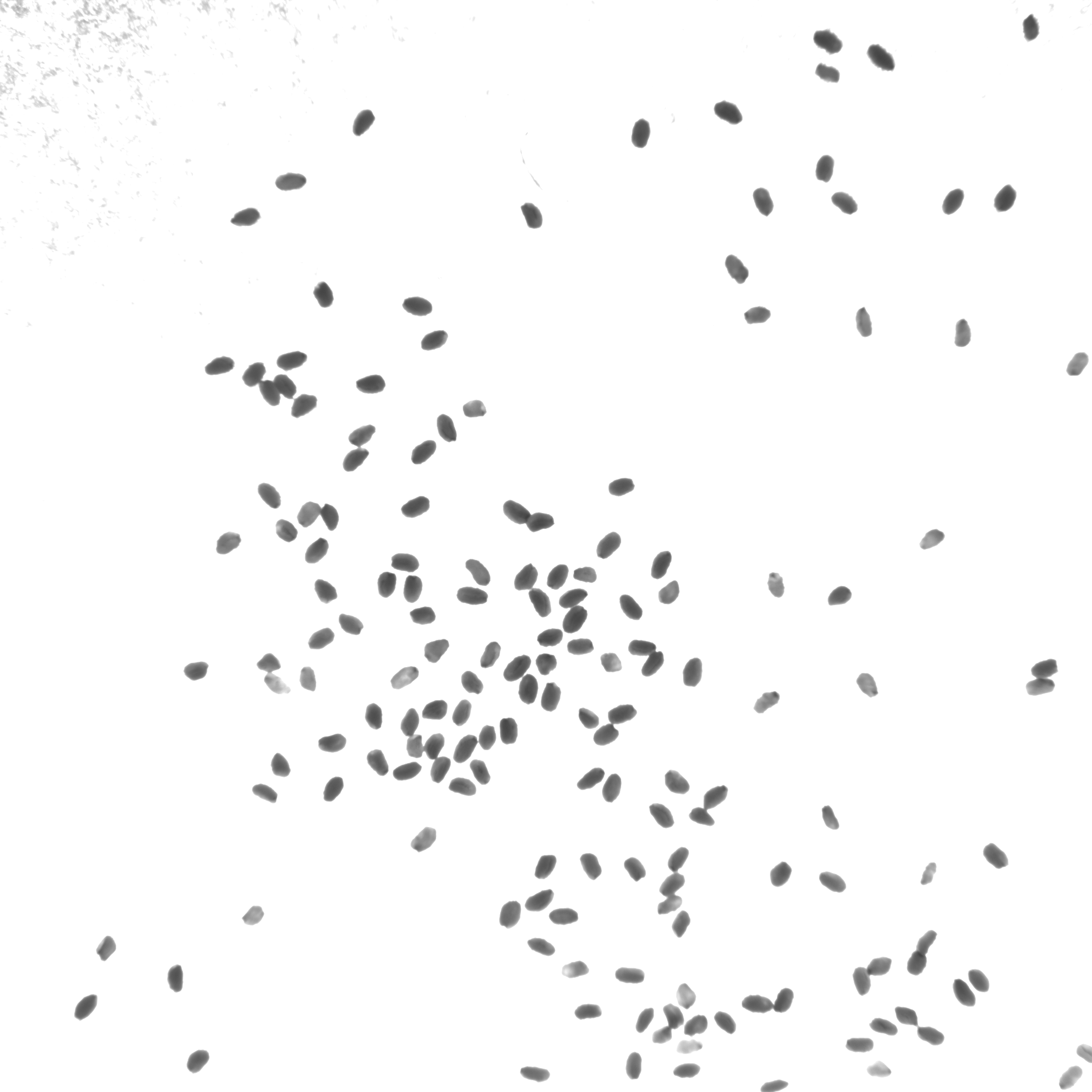

Supplement: Supplementary file 1 — Supplementary Material 1 [file 13007_2025_1406_MOESM1_ESM.zip › performance_comparison_images/VZ312-8_BF.tif]

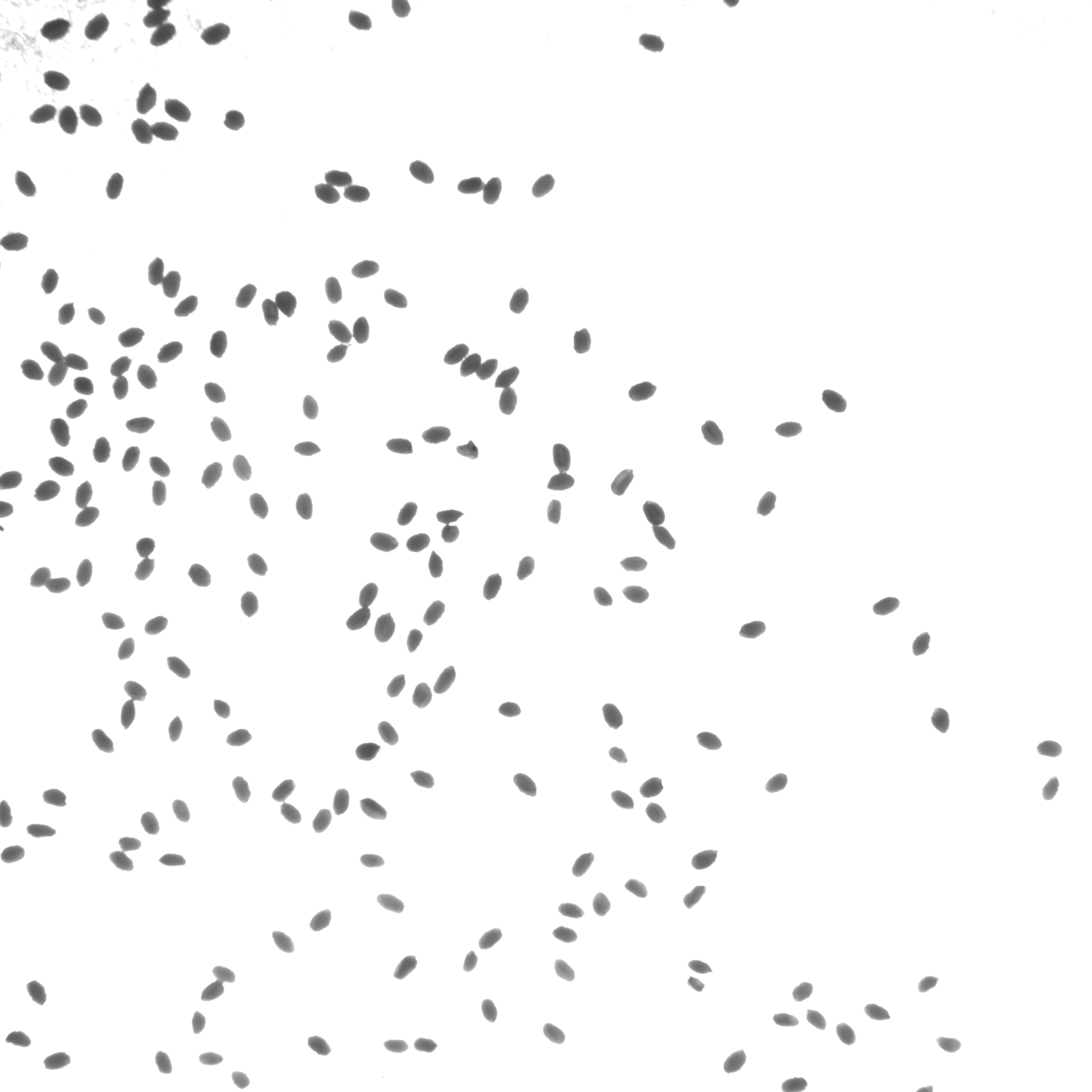

Supplement: Supplementary file 1 — Supplementary Material 1 [file 13007_2025_1406_MOESM1_ESM.zip › performance_comparison_images/VZ312-4_BF.tif]

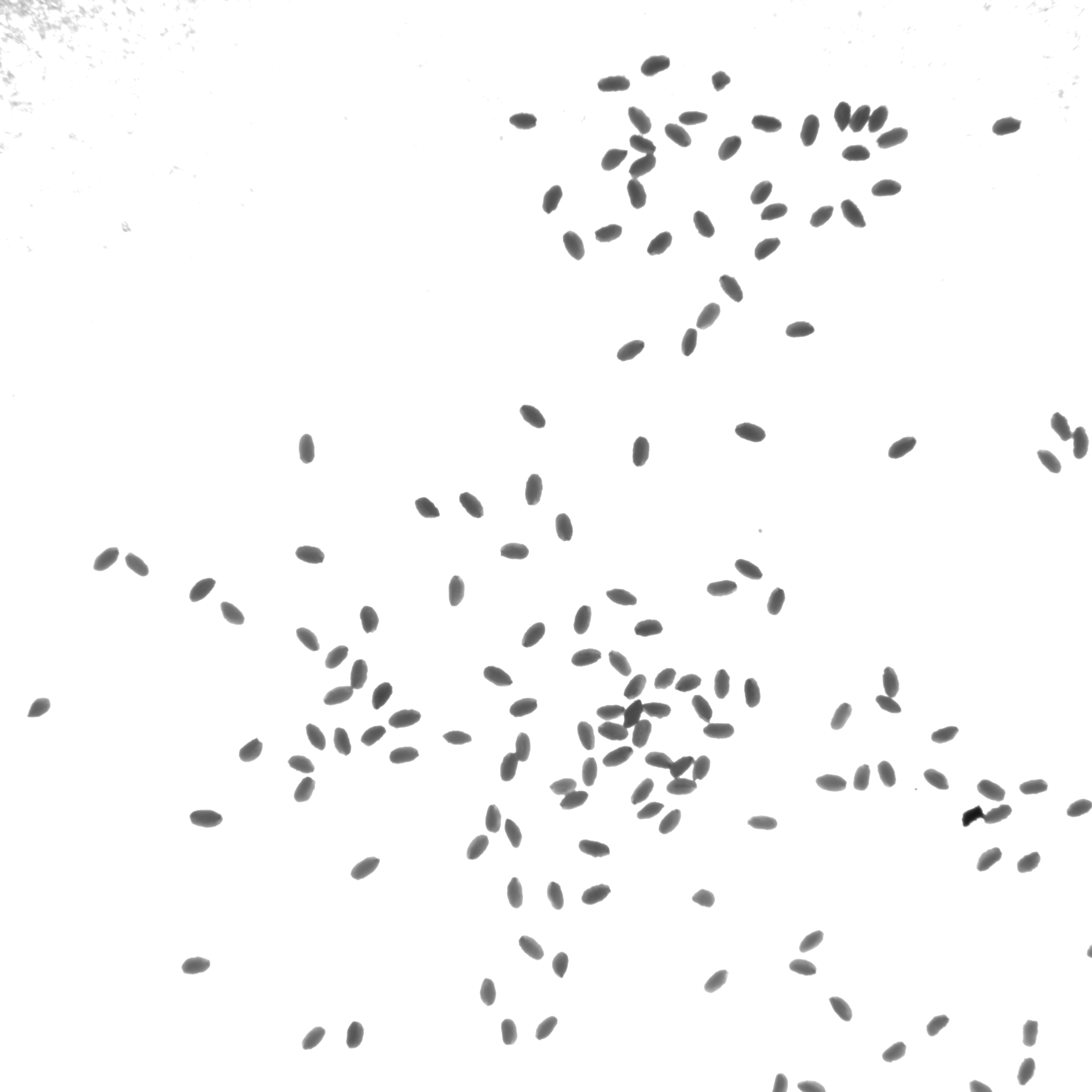

Supplement: Supplementary file 1 — Supplementary Material 1 [file 13007_2025_1406_MOESM1_ESM.zip › performance_comparison_images/VZ313-4_BF.tif]

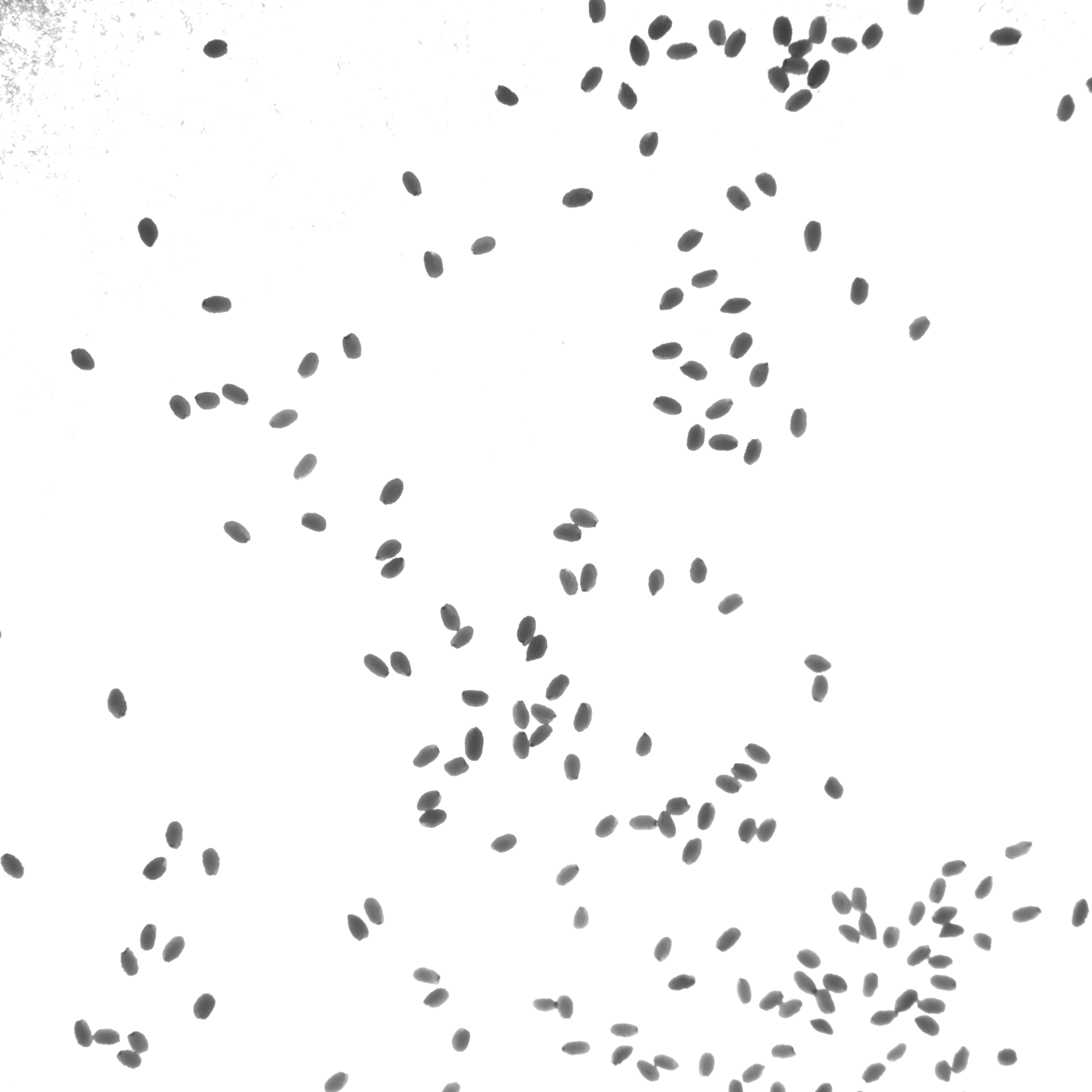

Supplement: Supplementary file 1 — Supplementary Material 1 [file 13007_2025_1406_MOESM1_ESM.zip › performance_comparison_images/VZ314-15_BF.tif]

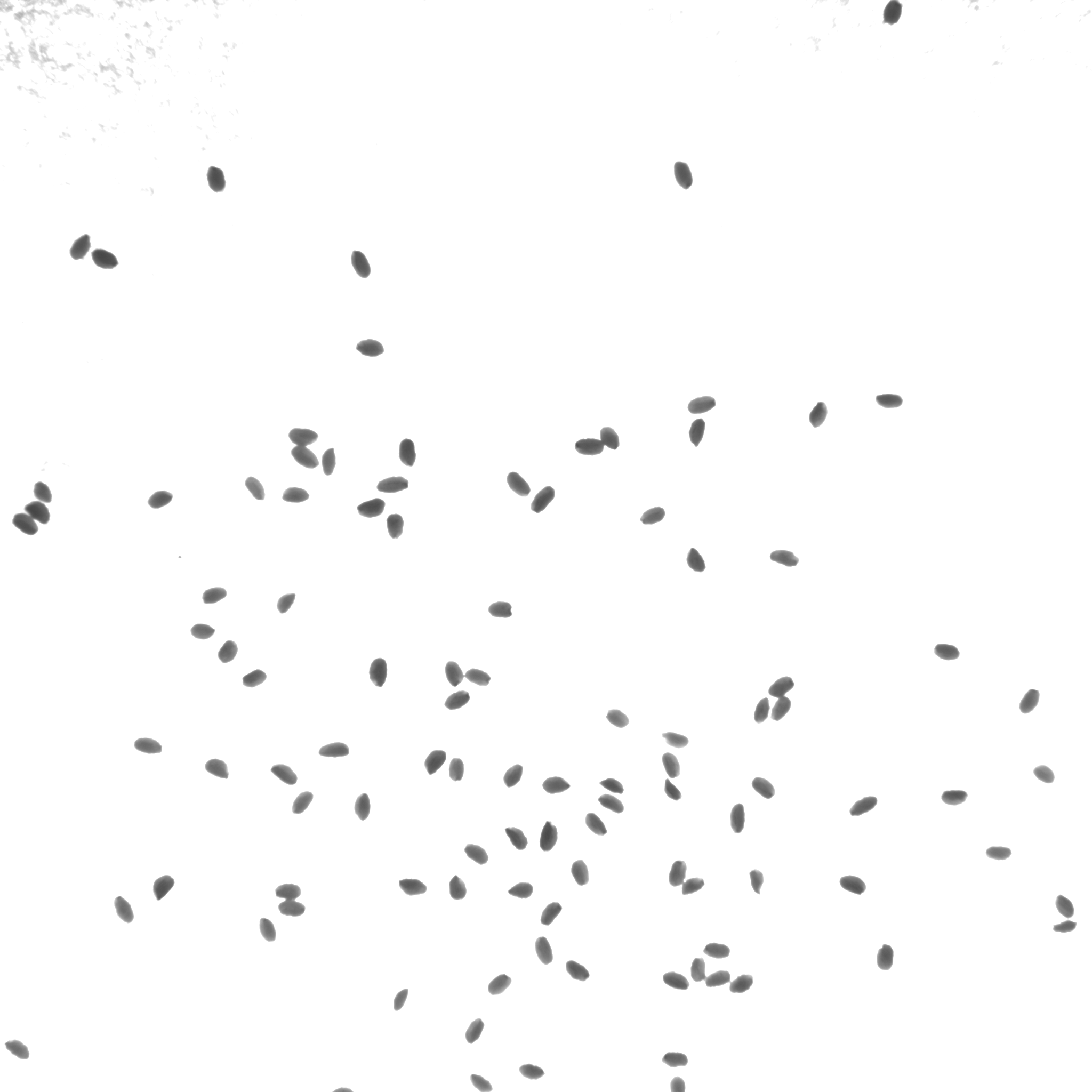

Supplement: Supplementary file 1 — Supplementary Material 1 [file 13007_2025_1406_MOESM1_ESM.zip › performance_comparison_images/VZ314-19_BF.tif]

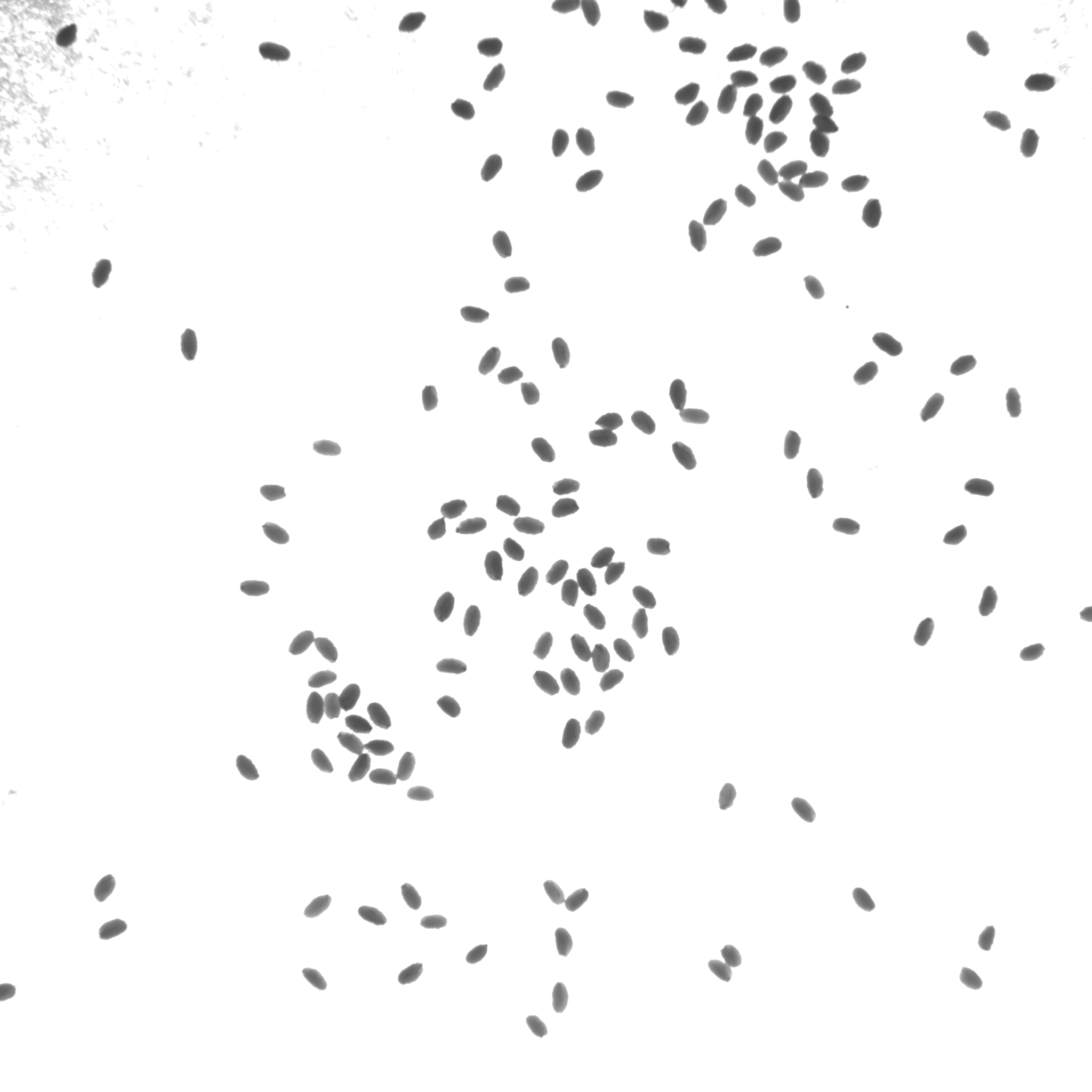

Supplement: Supplementary file 1 — Supplementary Material 1 [file 13007_2025_1406_MOESM1_ESM.zip › performance_comparison_images/VZ313-6_BF.tif]

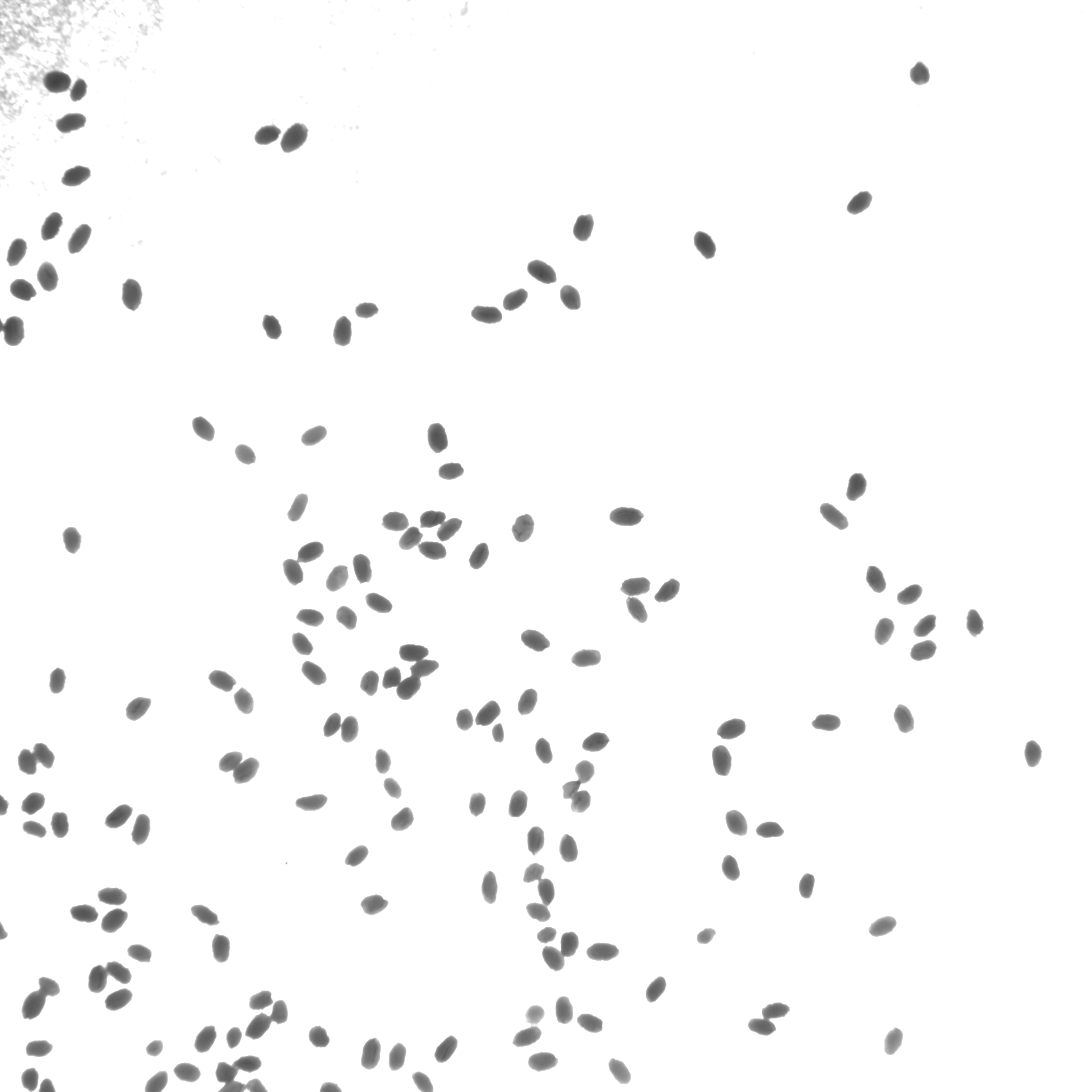

Supplement: Supplementary file 1 — Supplementary Material 1 [file 13007_2025_1406_MOESM1_ESM.zip › performance_comparison_images/VZ312-15_BF.tif]

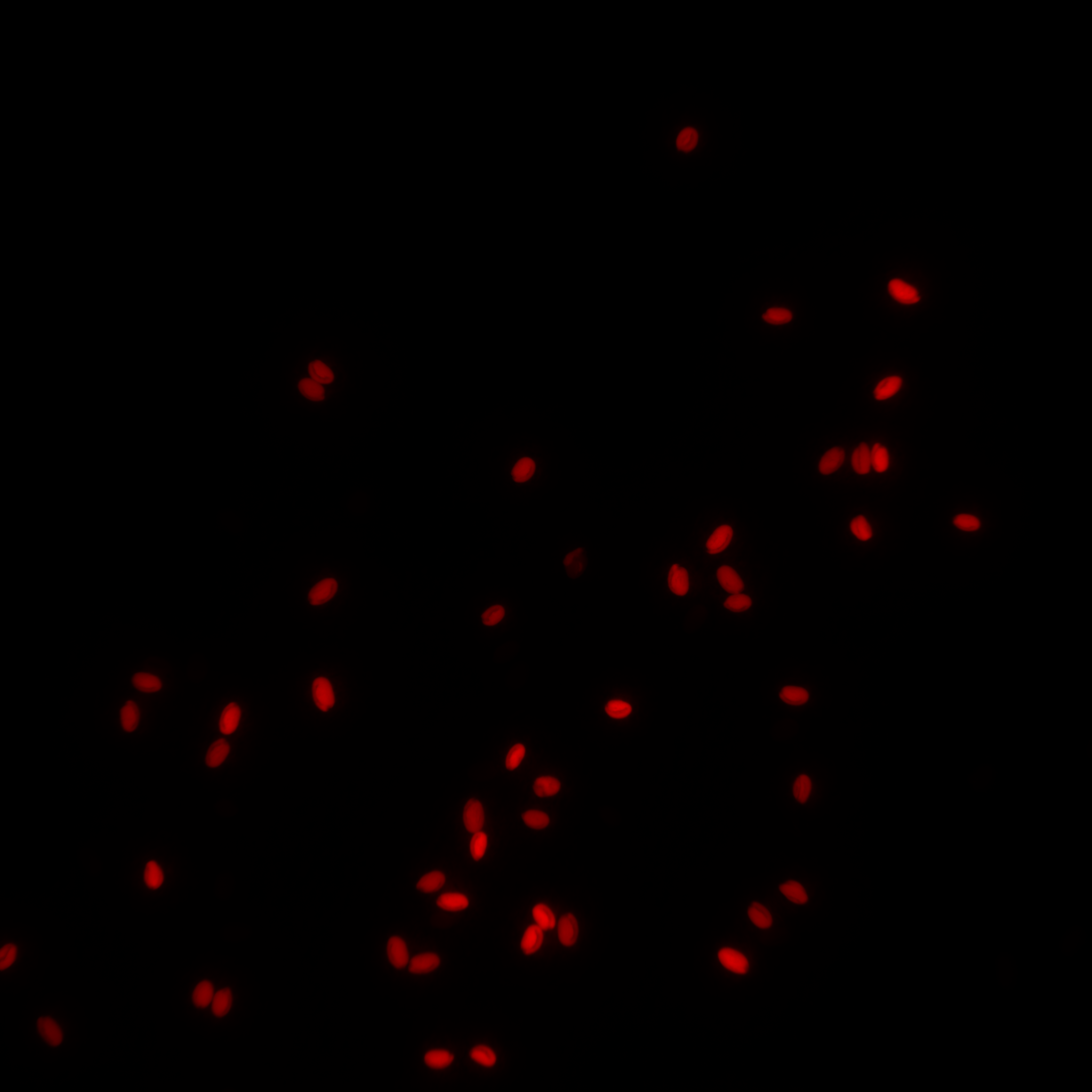

Supplement: Supplementary file 1 — Supplementary Material 1 [file 13007_2025_1406_MOESM1_ESM.zip › performance_comparison_images/VZ314-4_FL.tif]

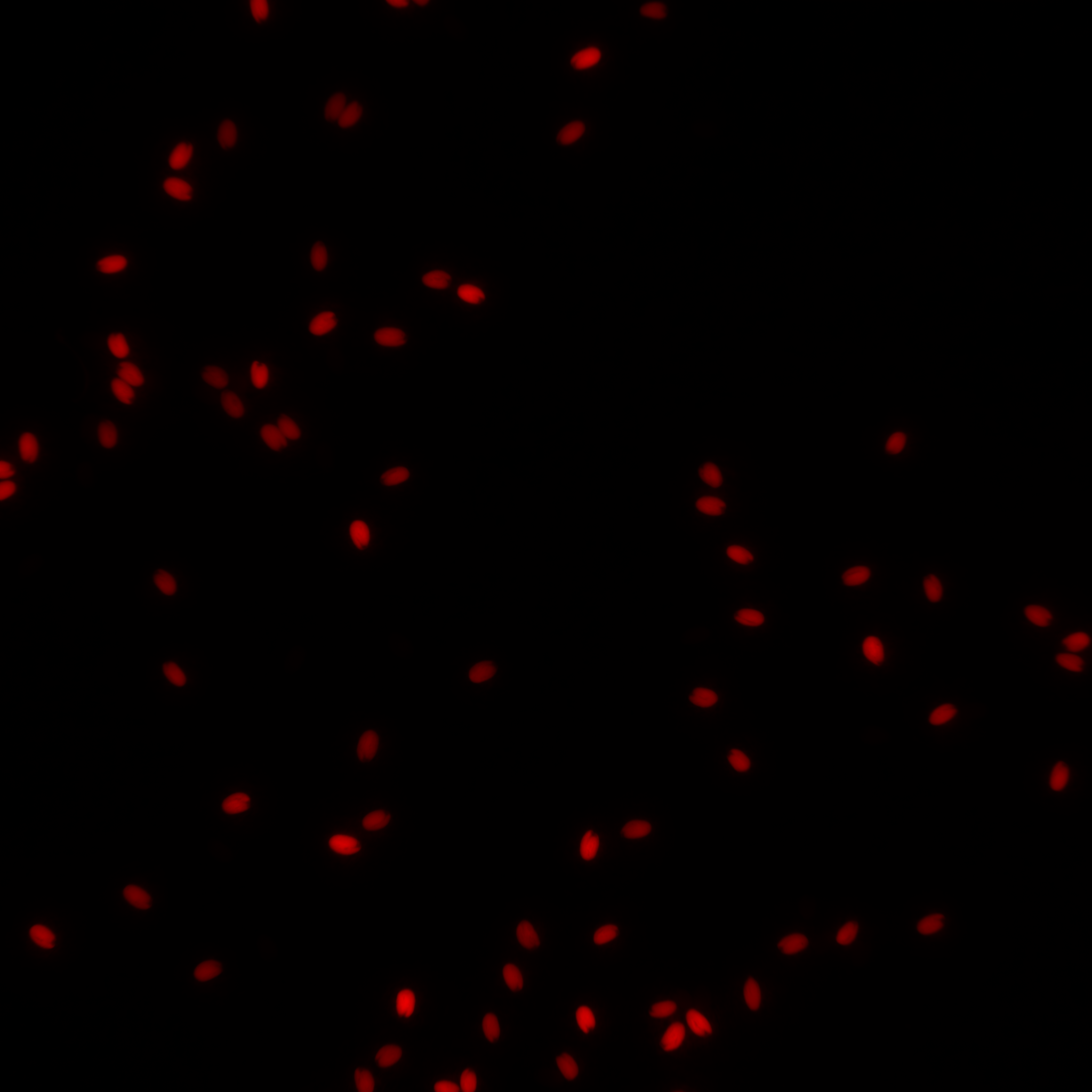

Supplement: Supplementary file 1 — Supplementary Material 1 [file 13007_2025_1406_MOESM1_ESM.zip › performance_comparison_images/VZ314-11_FL.tif]

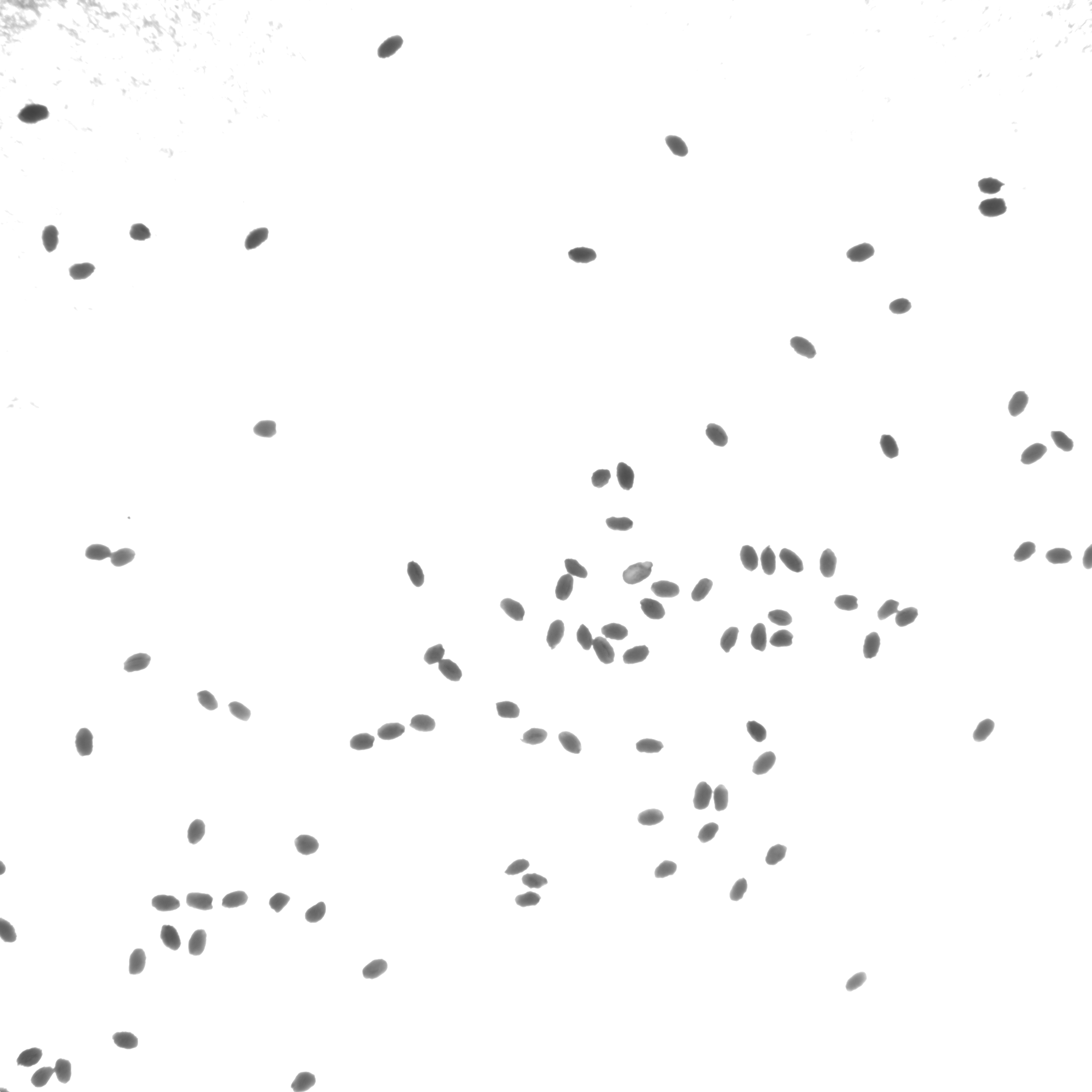

Supplement: Supplementary file 1 — Supplementary Material 1 [file 13007_2025_1406_MOESM1_ESM.zip › performance_comparison_images/VZ314-13_BF.tif]

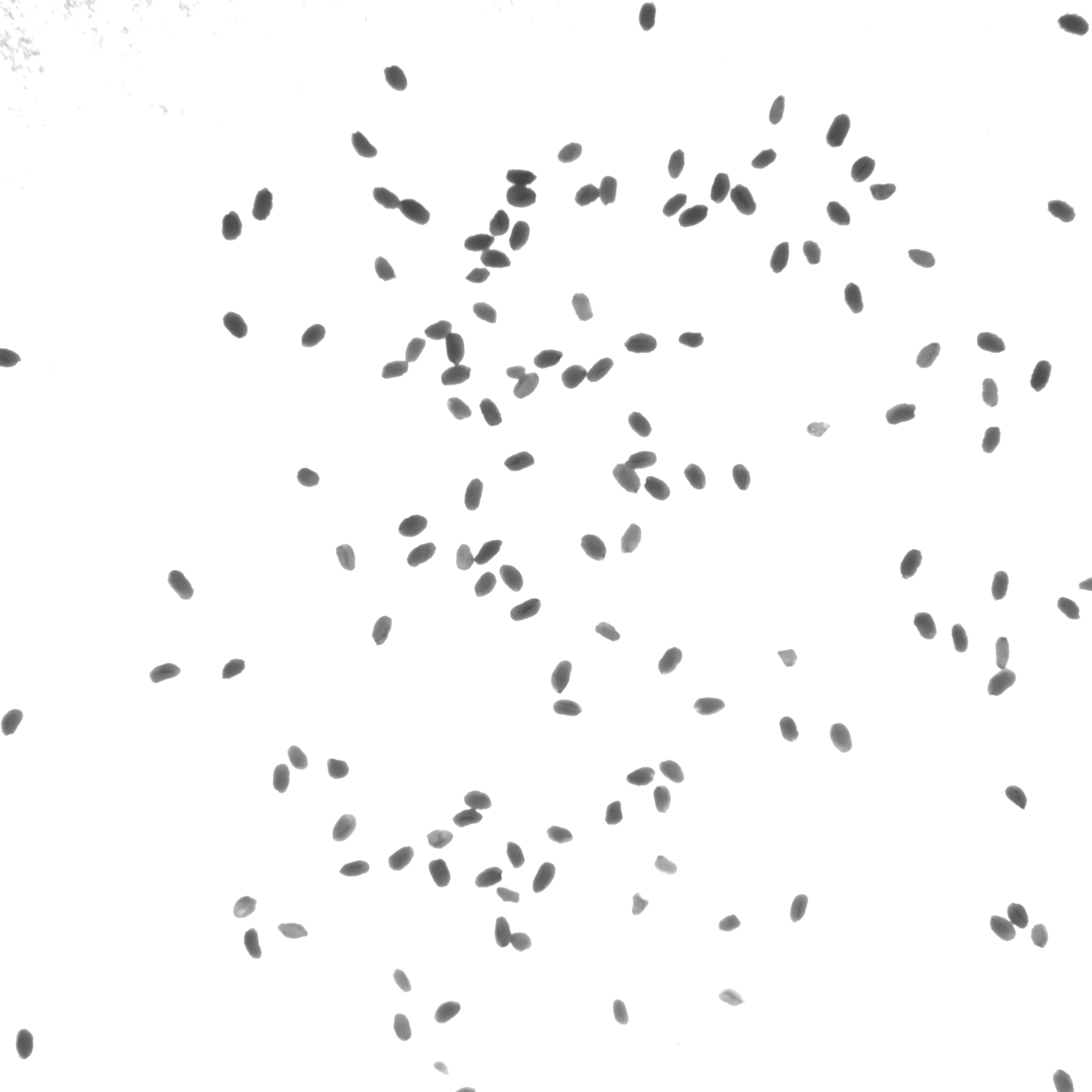

Supplement: Supplementary file 1 — Supplementary Material 1 [file 13007_2025_1406_MOESM1_ESM.zip › performance_comparison_images/VZ312-2_BF.tif]

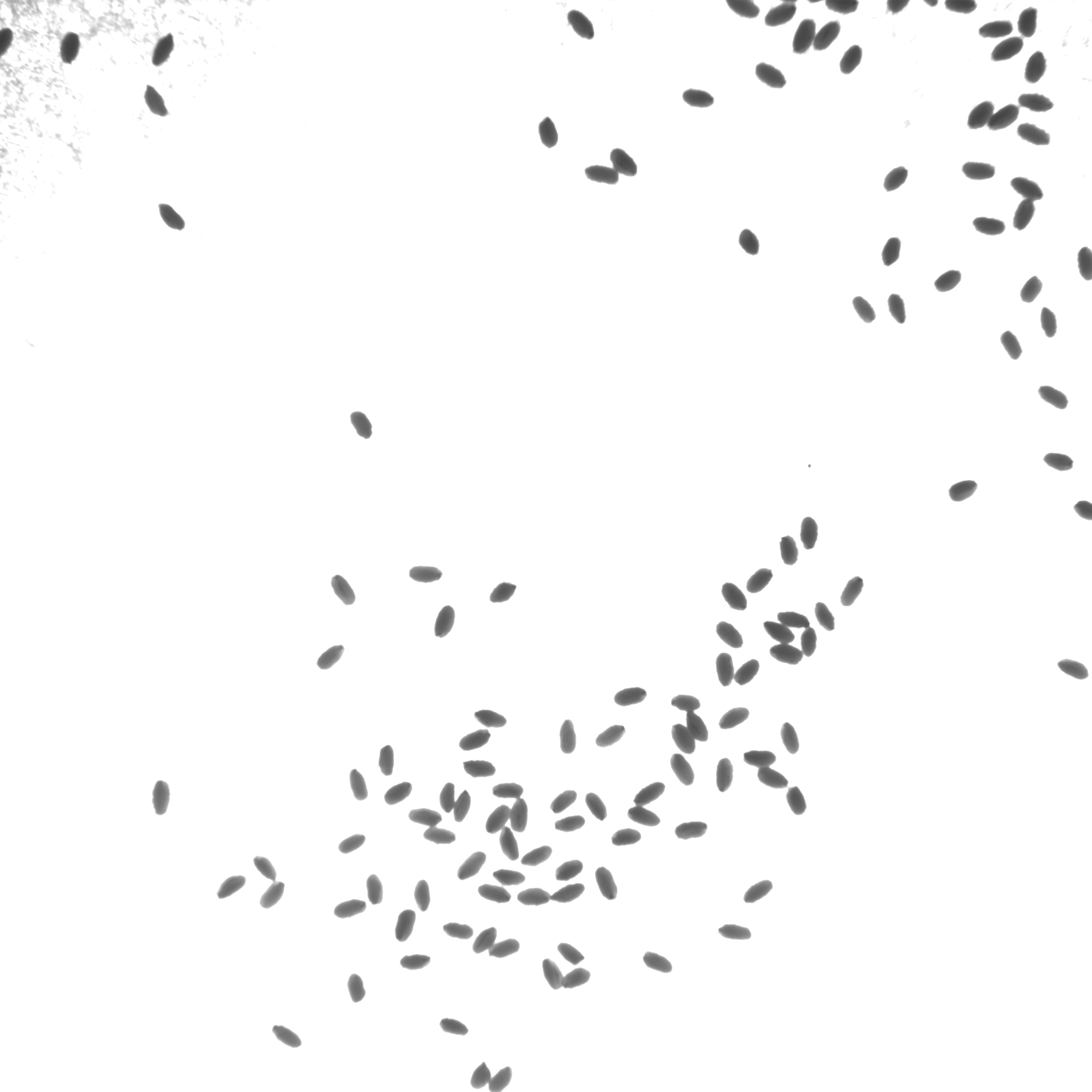

Supplement: Supplementary file 1 — Supplementary Material 1 [file 13007_2025_1406_MOESM1_ESM.zip › performance_comparison_images/VZ313-2_BF.tif]

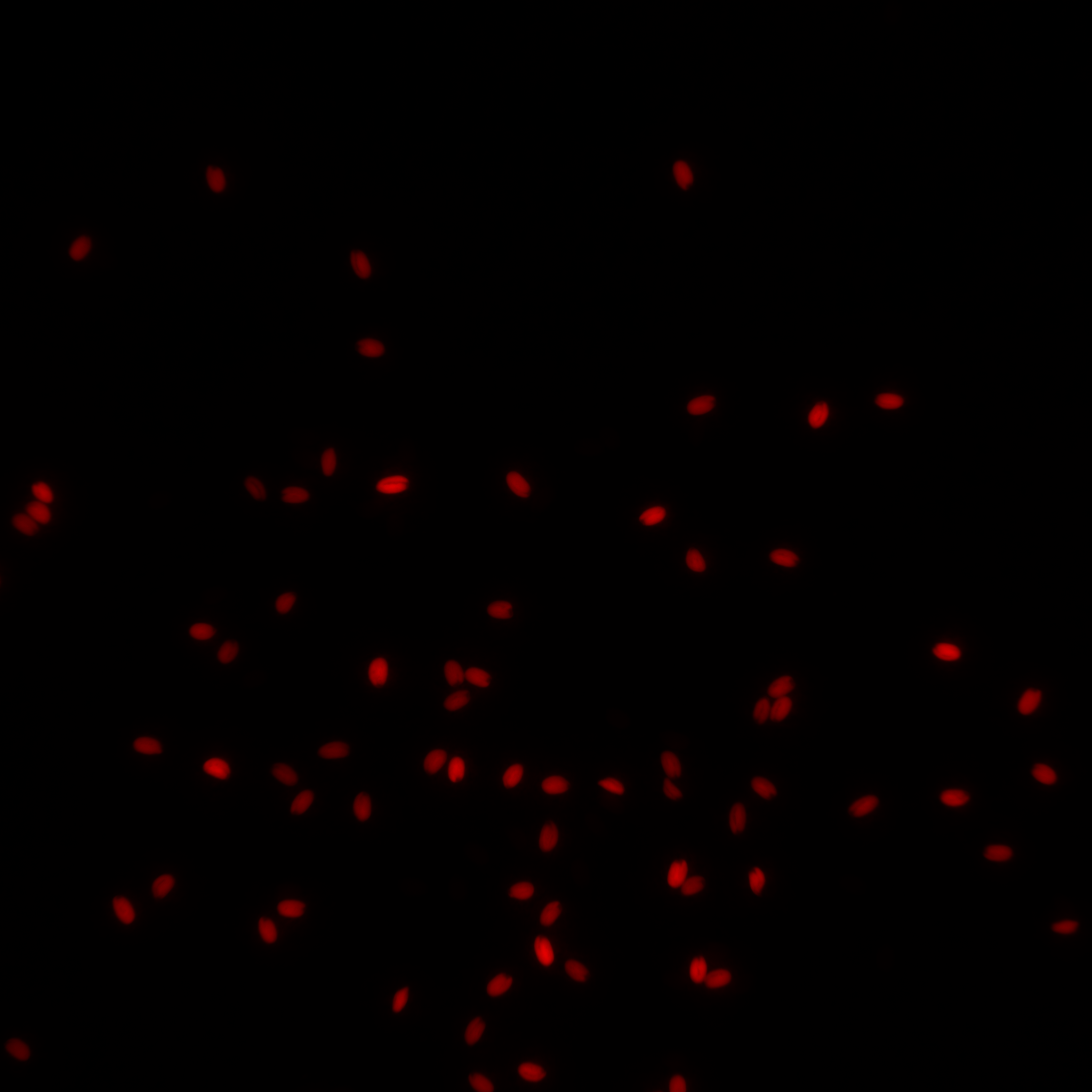

Supplement: Supplementary file 1 — Supplementary Material 1 [file 13007_2025_1406_MOESM1_ESM.zip › performance_comparison_images/VZ314-19_FL.tif]

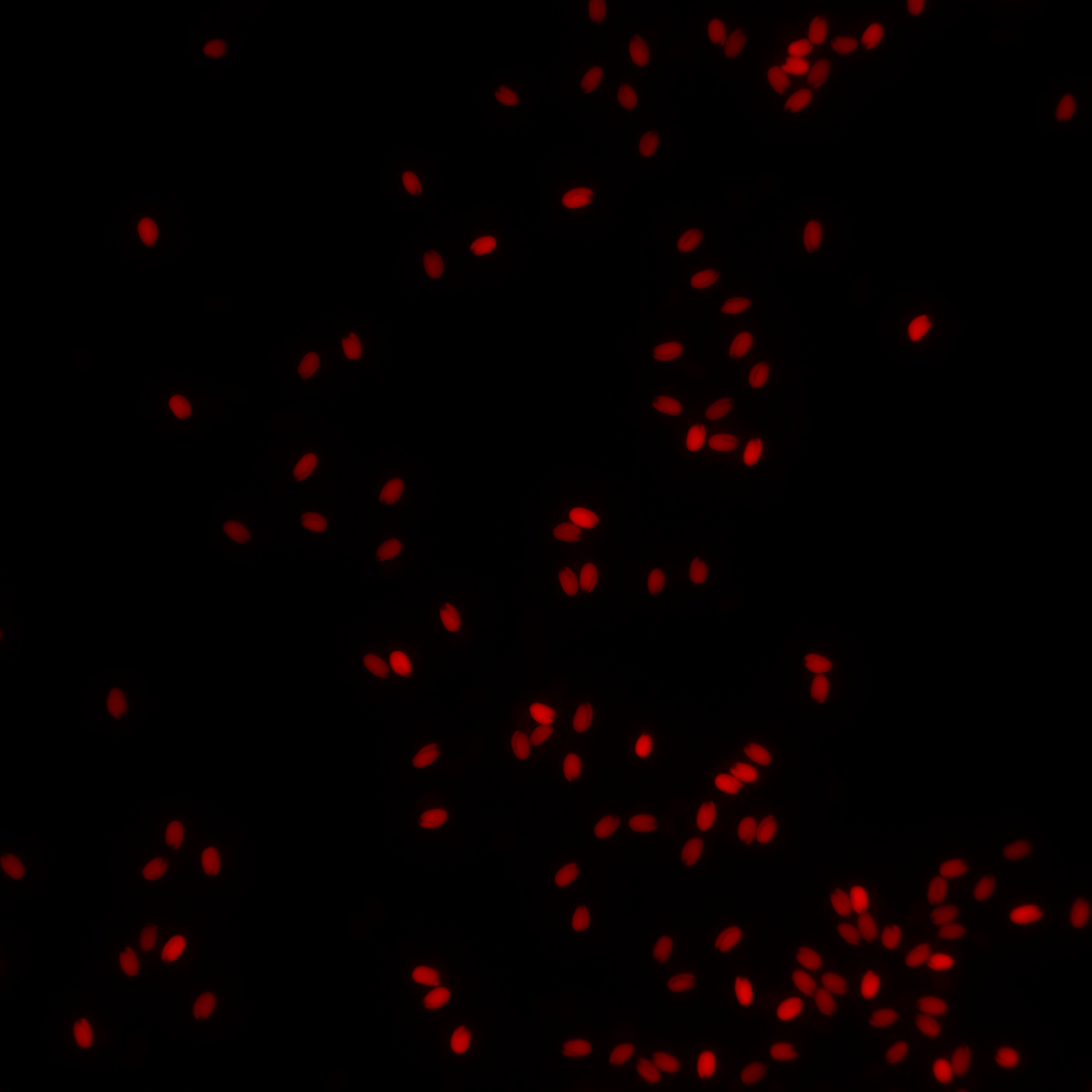

Supplement: Supplementary file 1 — Supplementary Material 1 [file 13007_2025_1406_MOESM1_ESM.zip › performance_comparison_images/VZ314-15_FL.tif]

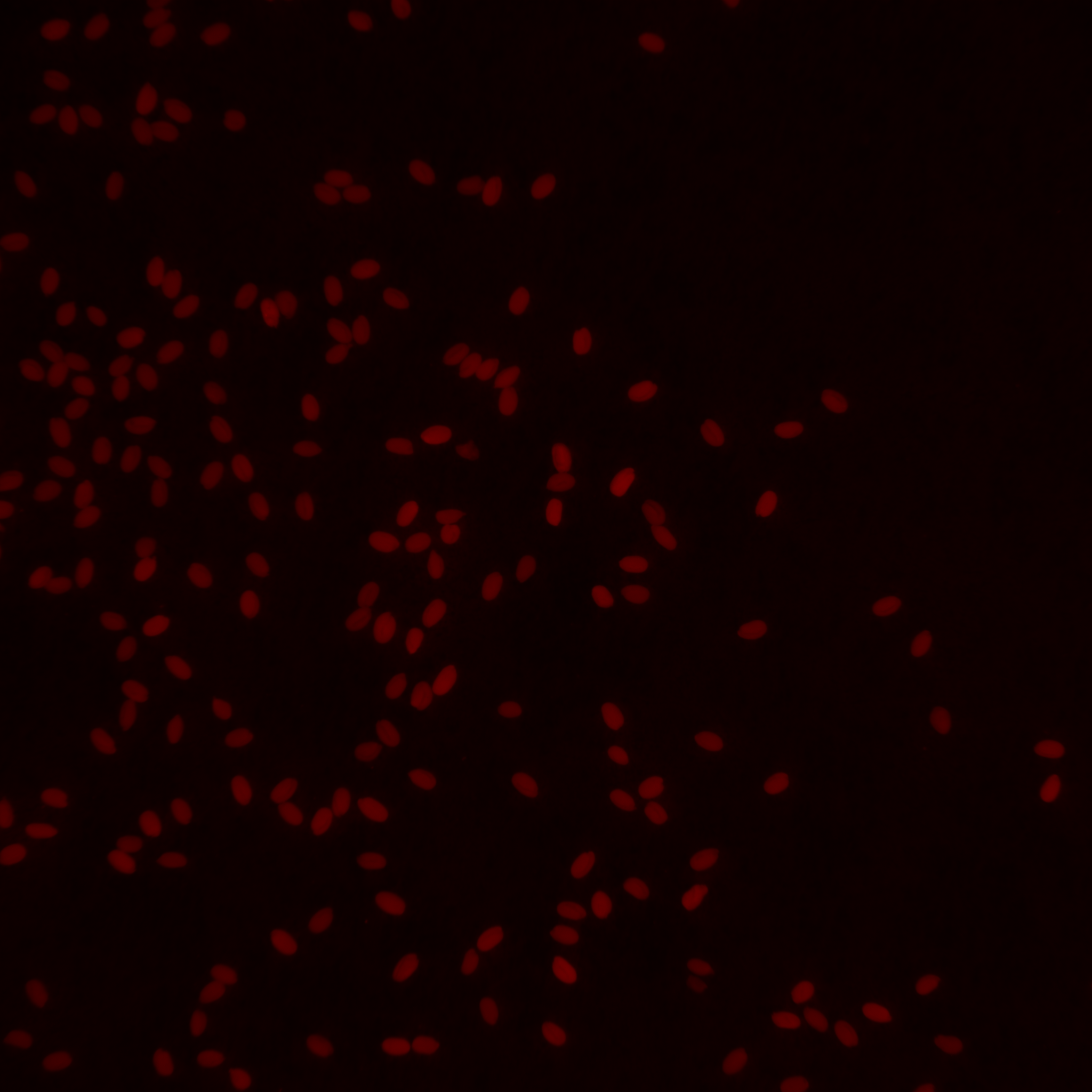

Supplement: Supplementary file 1 — Supplementary Material 1 [file 13007_2025_1406_MOESM1_ESM.zip › performance_comparison_images/VZ312-4_FL.tif]

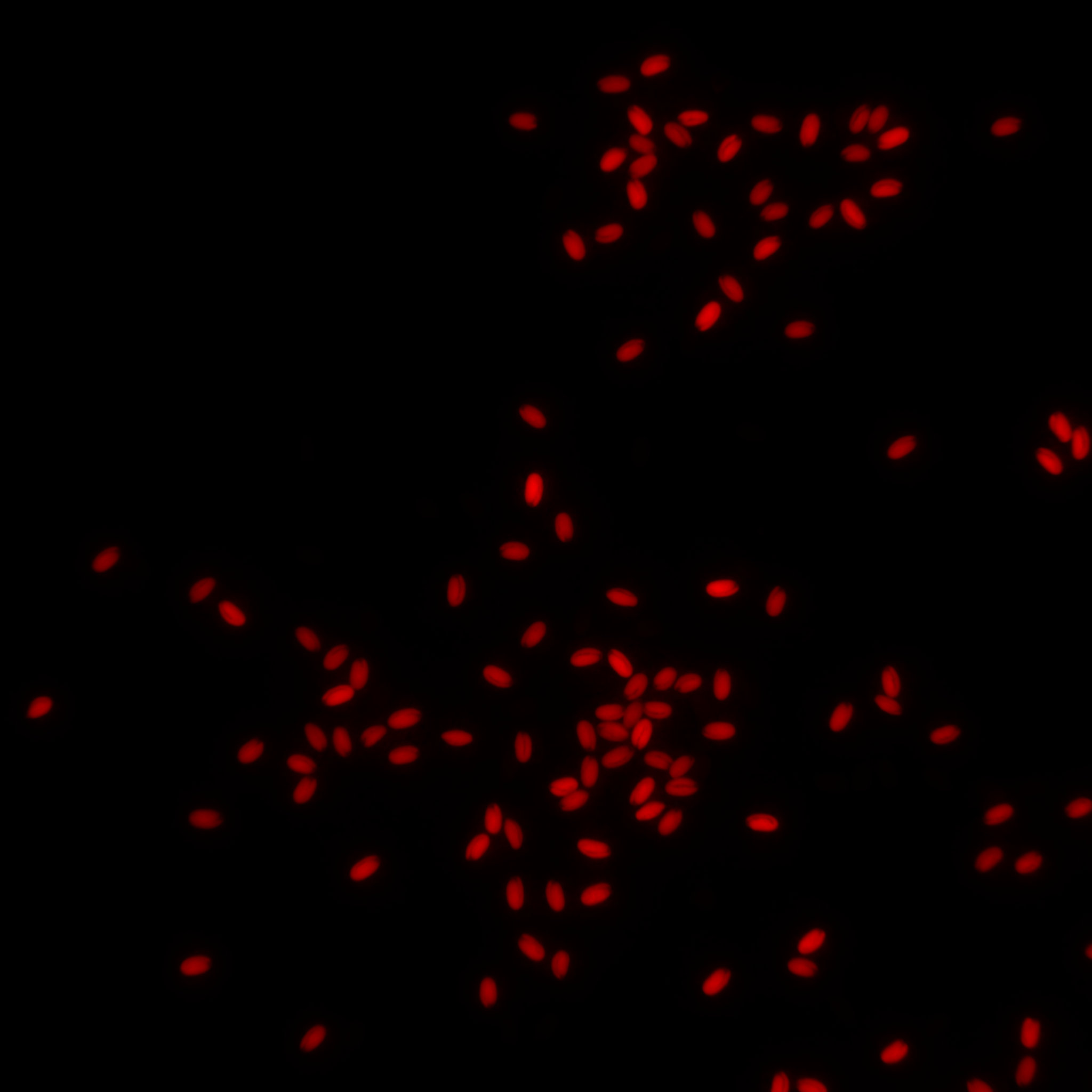

Supplement: Supplementary file 1 — Supplementary Material 1 [file 13007_2025_1406_MOESM1_ESM.zip › performance_comparison_images/VZ313-4_FL.tif]

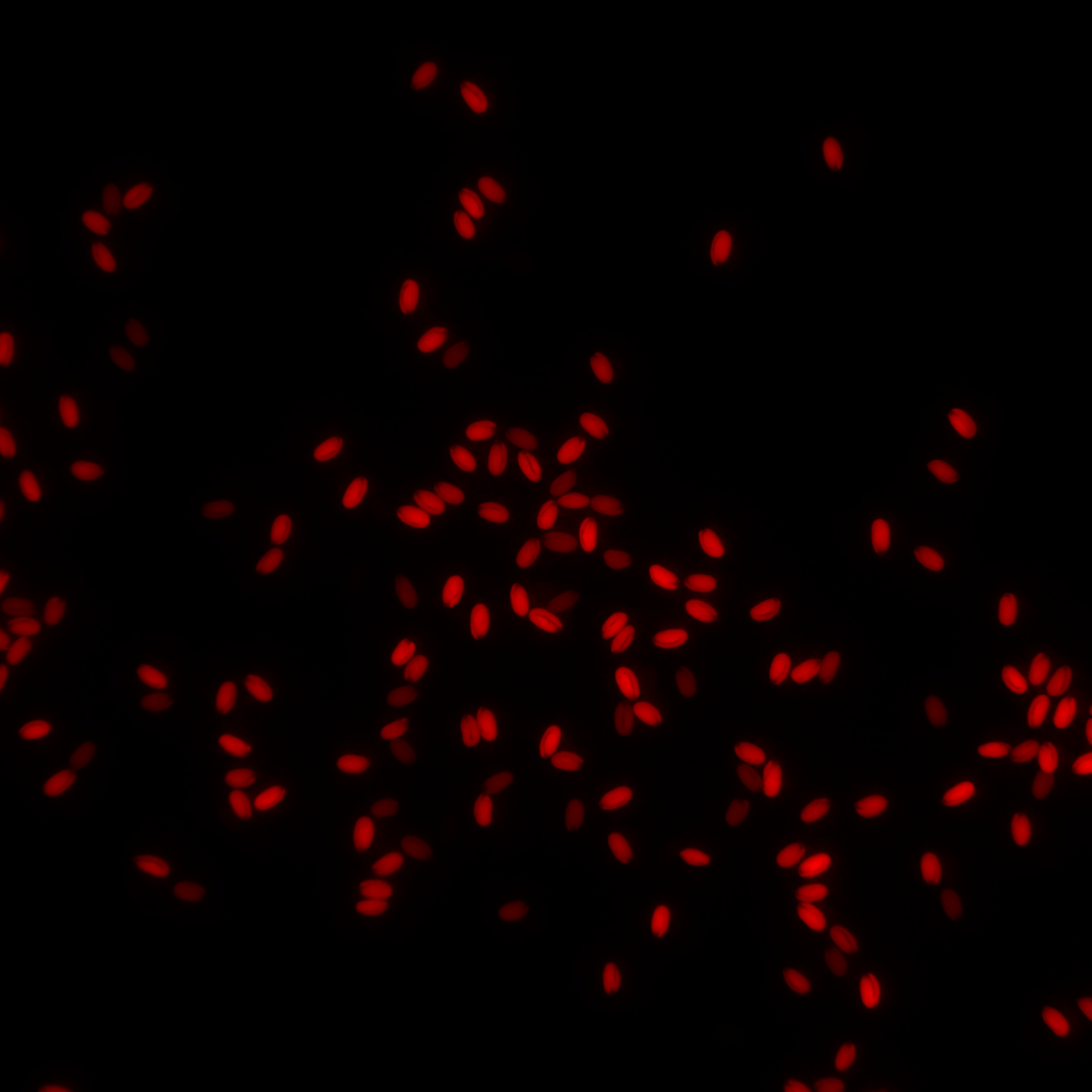

Supplement: Supplementary file 1 — Supplementary Material 1 [file 13007_2025_1406_MOESM1_ESM.zip › performance_comparison_images/VZ313-8_FL.tif]

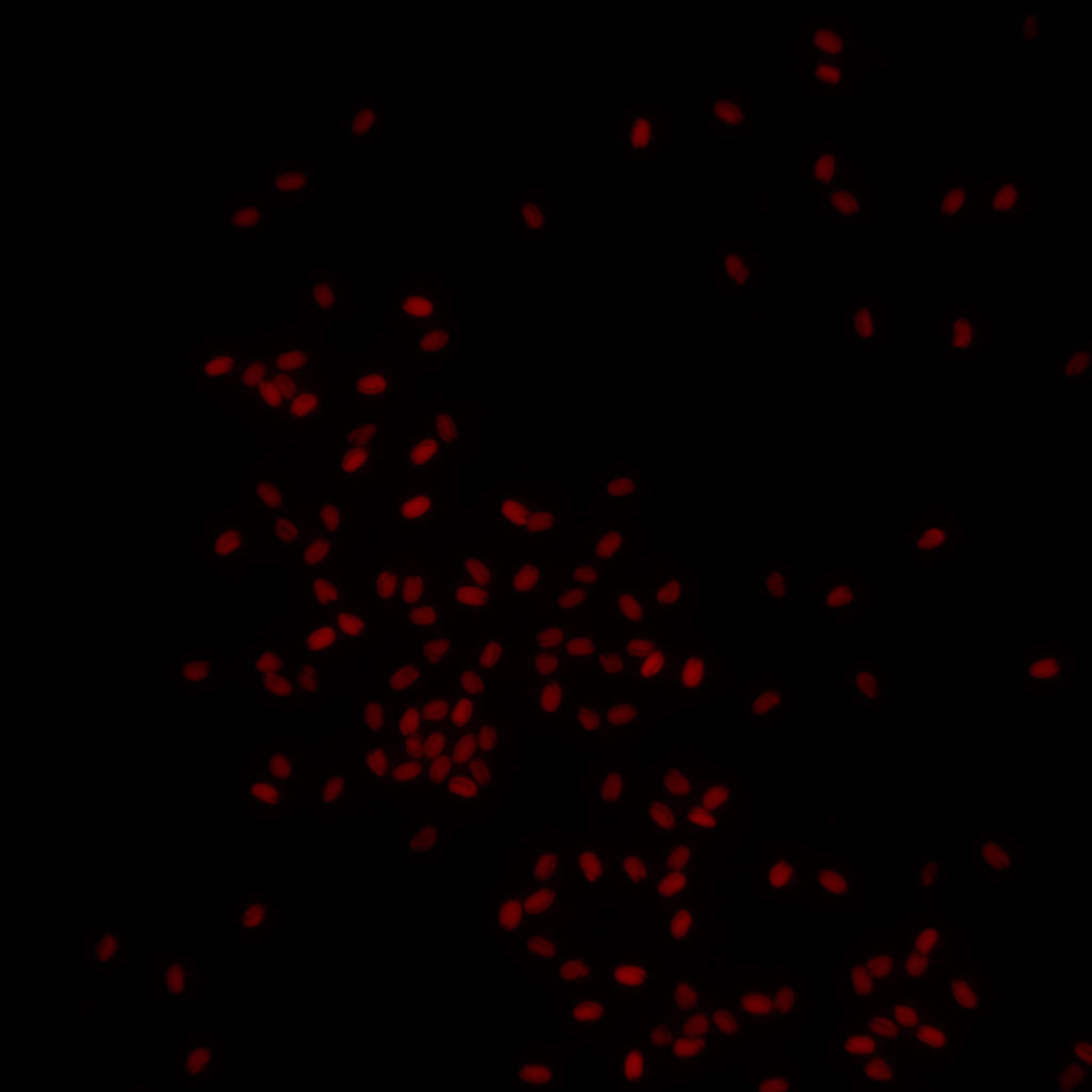

Supplement: Supplementary file 1 — Supplementary Material 1 [file 13007_2025_1406_MOESM1_ESM.zip › performance_comparison_images/VZ312-8_FL.tif]

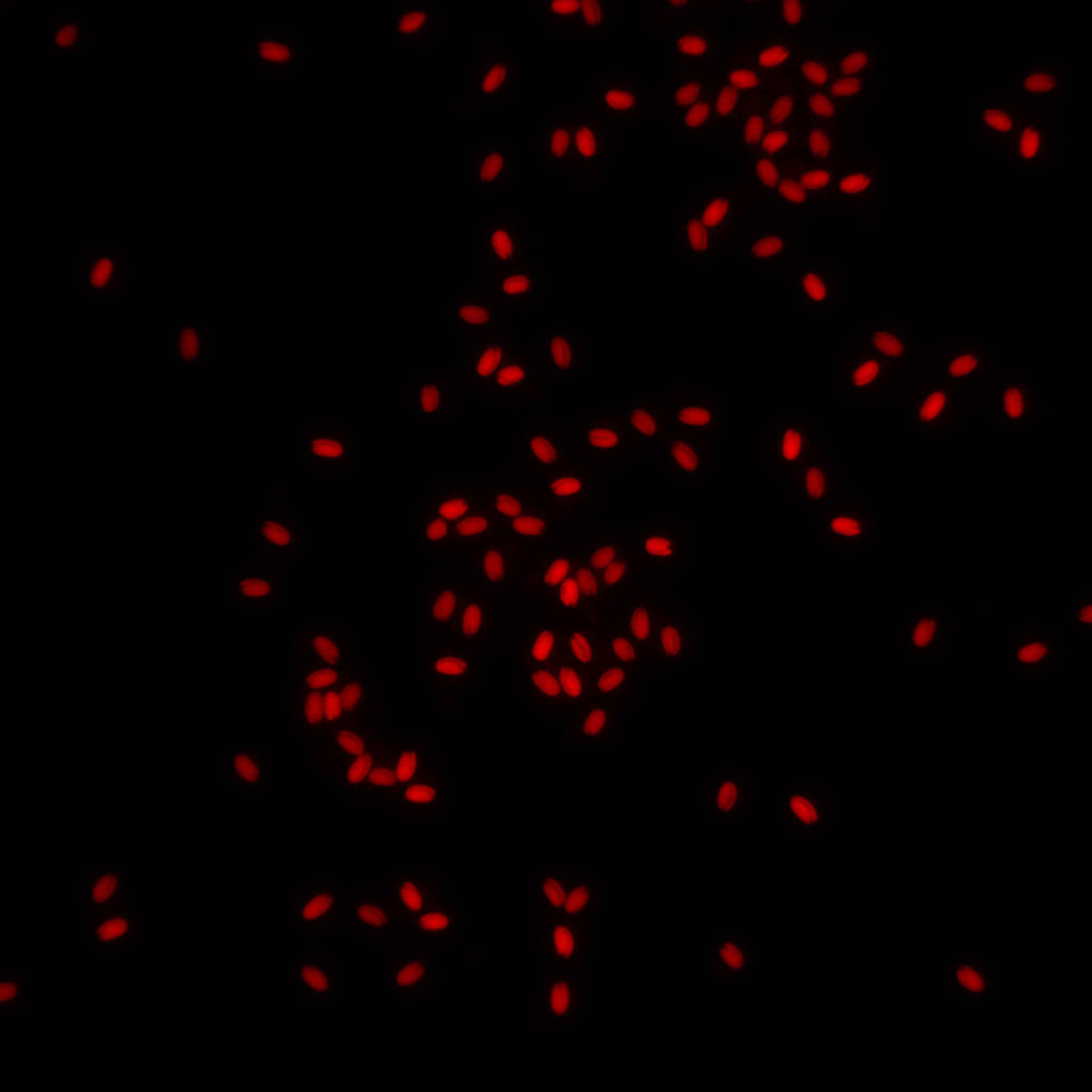

Supplement: Supplementary file 1 — Supplementary Material 1 [file 13007_2025_1406_MOESM1_ESM.zip › performance_comparison_images/VZ313-6_FL.tif]

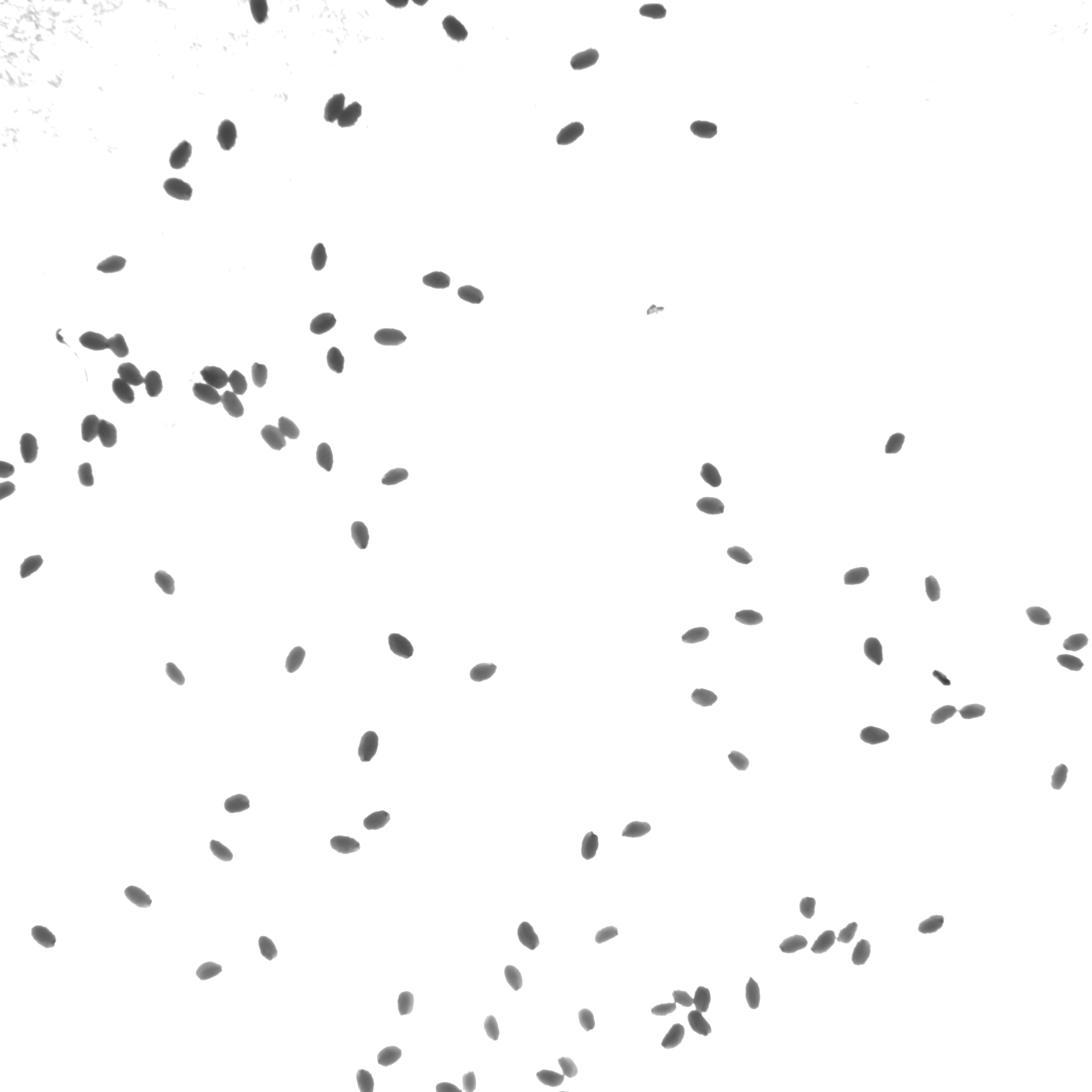

Supplement: Supplementary file 1 — Supplementary Material 1 [file 13007_2025_1406_MOESM1_ESM.zip › performance_comparison_images/VZ314-11_BF.tif]

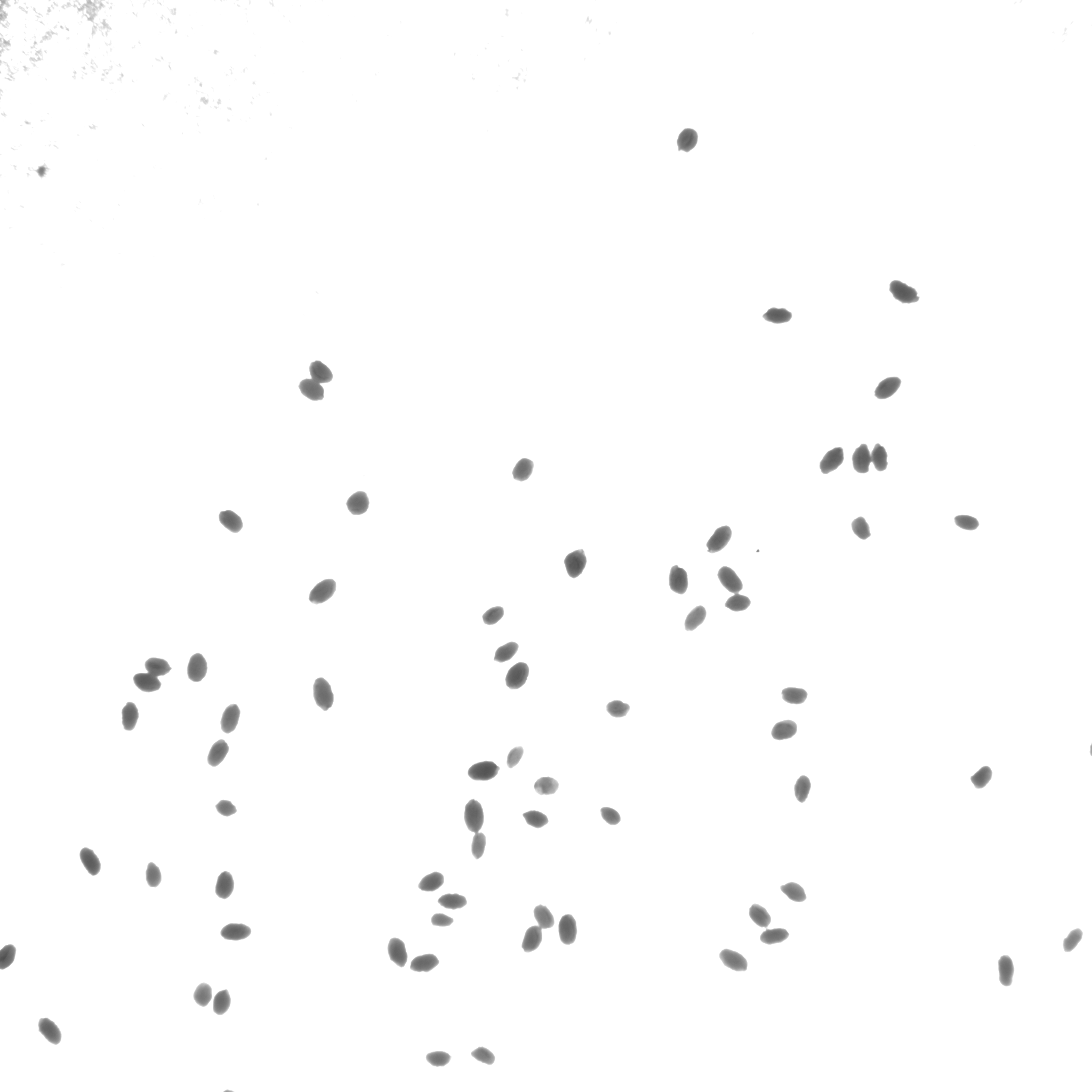

Supplement: Supplementary file 1 — Supplementary Material 1 [file 13007_2025_1406_MOESM1_ESM.zip › performance_comparison_images/VZ314-4_BF.tif]

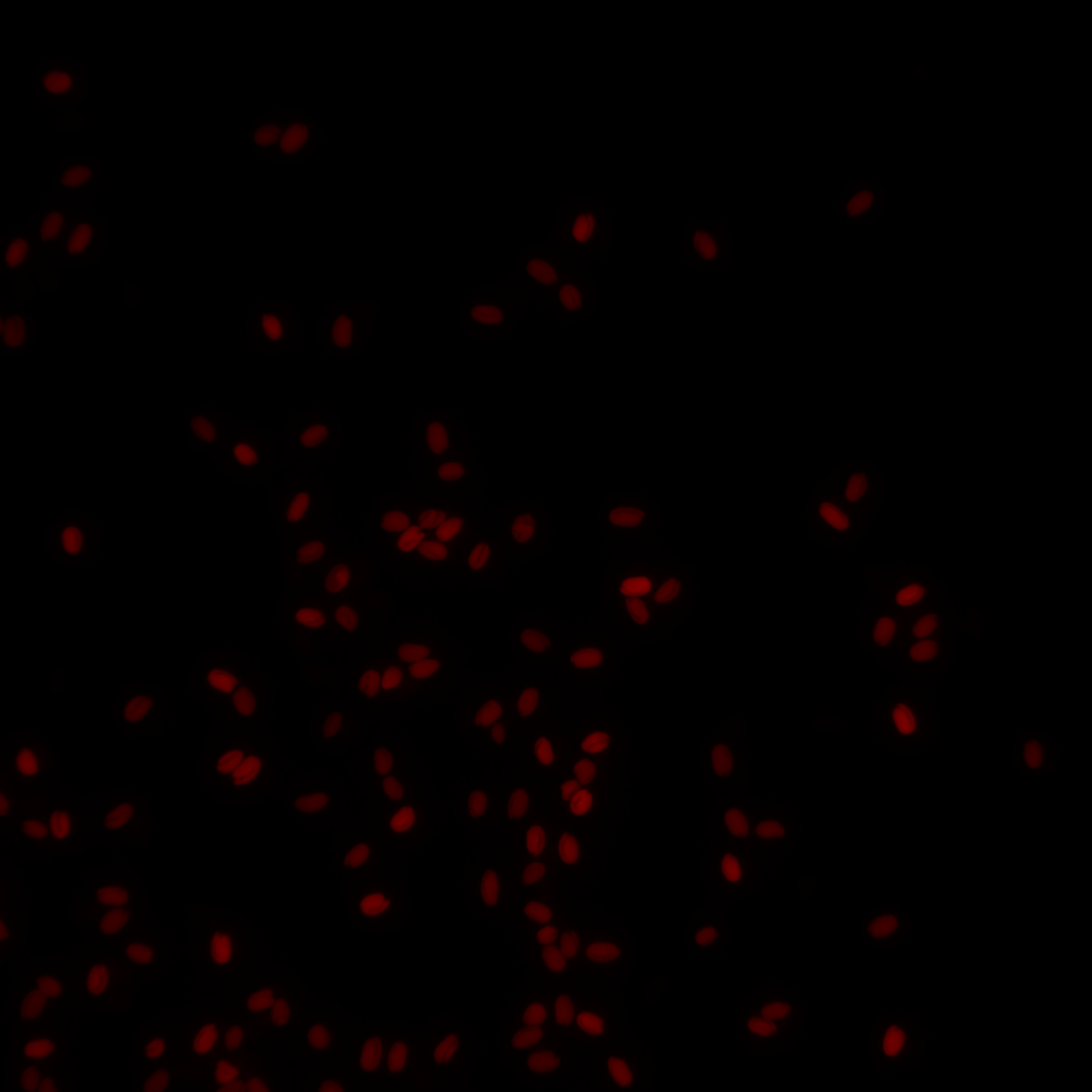

Supplement: Supplementary file 1 — Supplementary Material 1 [file 13007_2025_1406_MOESM1_ESM.zip › performance_comparison_images/VZ312-15_FL.tif]

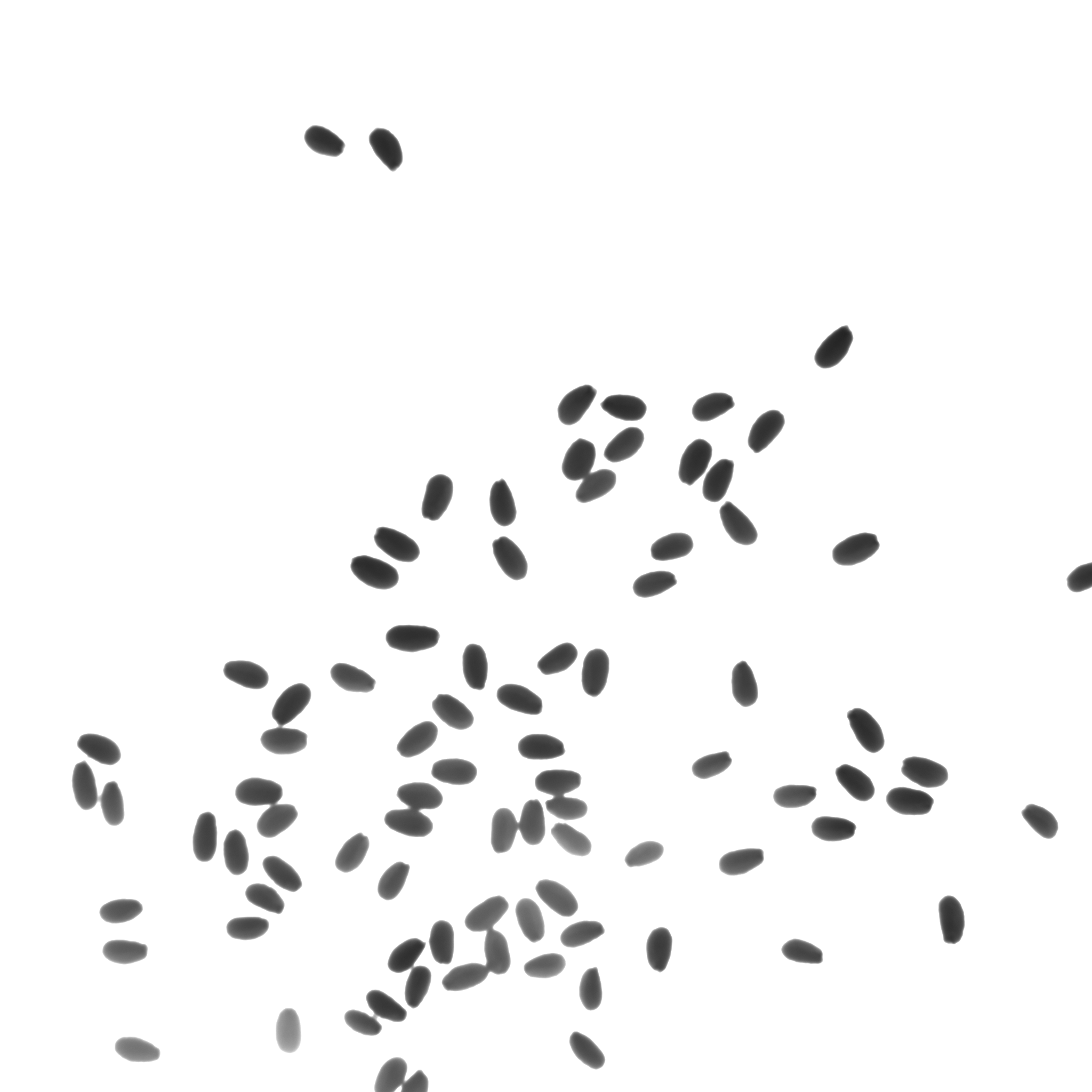

Supplement: Supplementary file 3 — Supplementary Material 3 [file 13007_2025_1406_MOESM3_ESM.zip › additional_markers_and_species_images/Crubella_BF.tif]

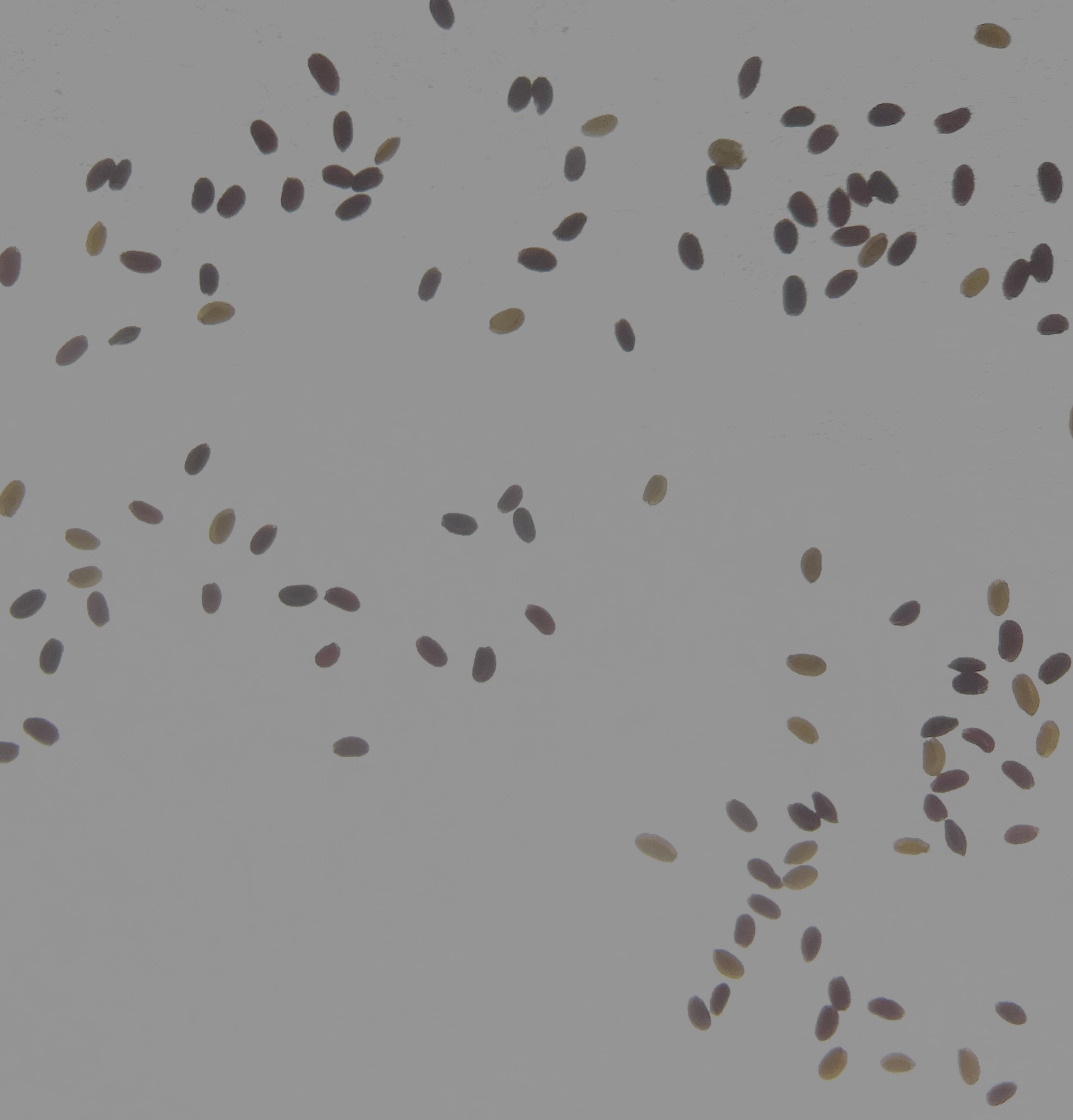

Supplement: Supplementary file 3 — Supplementary Material 3 [file 13007_2025_1406_MOESM3_ESM.zip › additional_markers_and_species_images/RUBY.jpg]

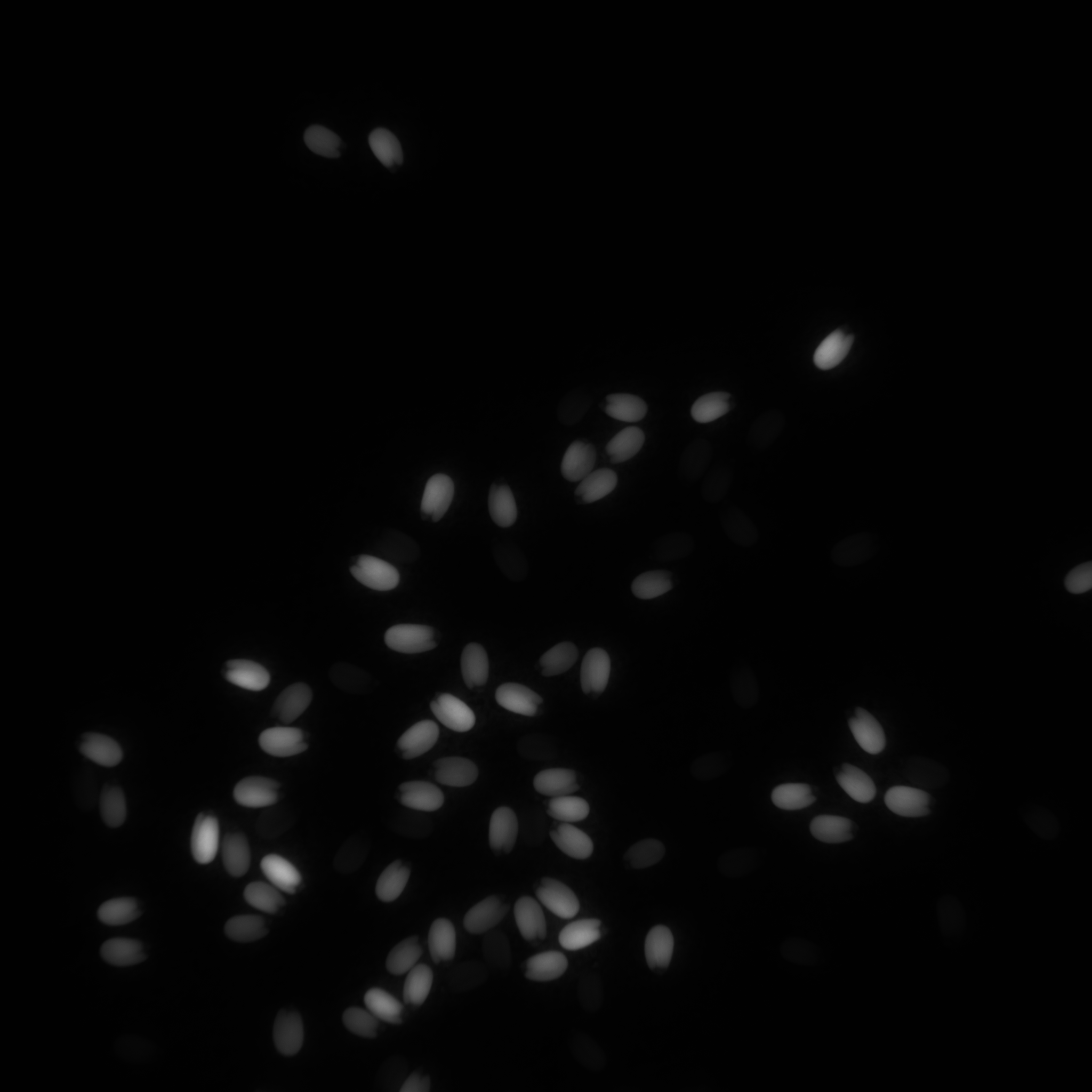

Supplement: Supplementary file 3 — Supplementary Material 3 [file 13007_2025_1406_MOESM3_ESM.zip › additional_markers_and_species_images/Crubella_FL.tif]

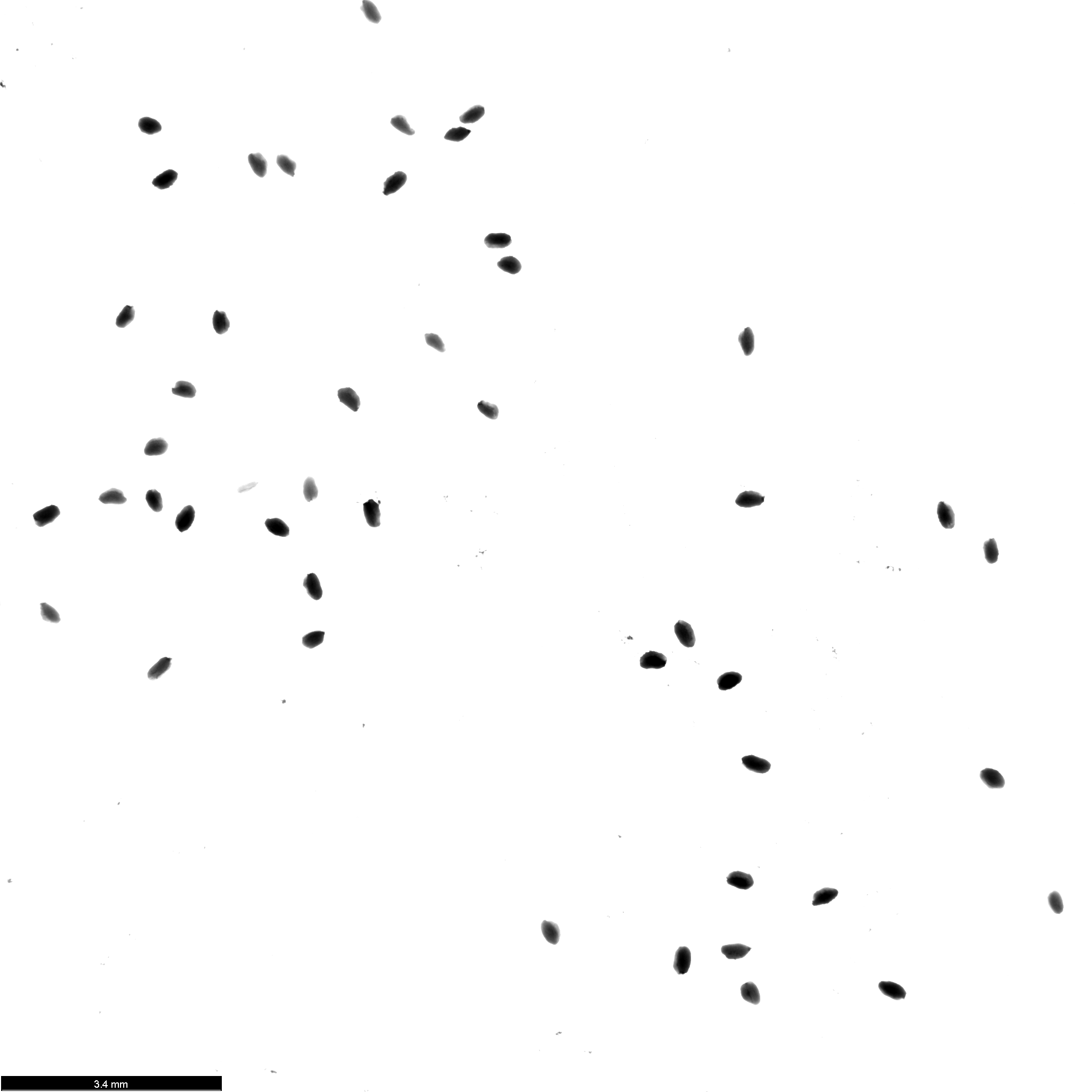

Supplement: Supplementary file 3 — Supplementary Material 3 [file 13007_2025_1406_MOESM3_ESM.zip › additional_markers_and_species_images/FastGreen_BF.tif]

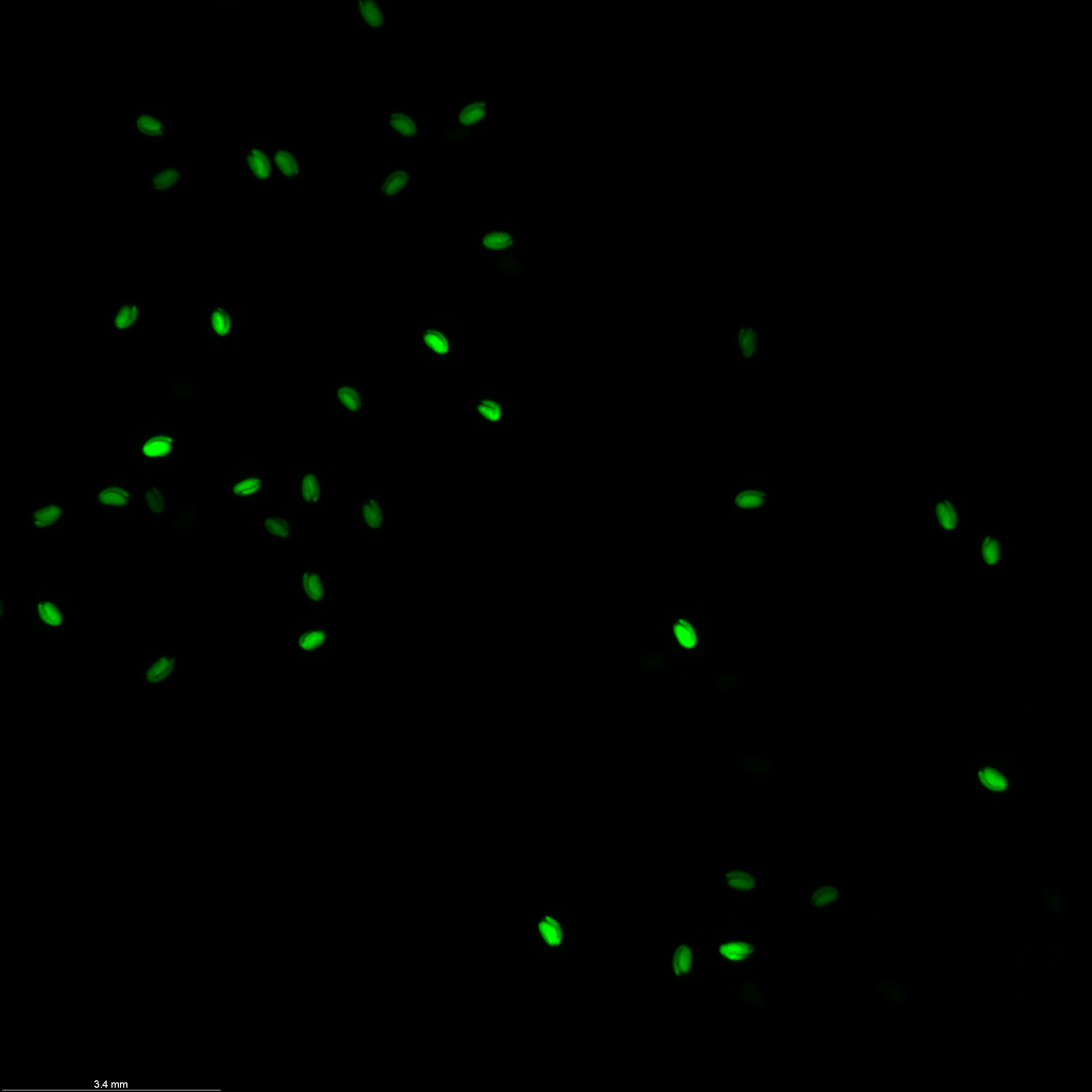

Supplement: Supplementary file 3 — Supplementary Material 3 [file 13007_2025_1406_MOESM3_ESM.zip › additional_markers_and_species_images/FastGreen_FL.tif]
