# Supplementary material for: SeedSeg: image-based transgenic seed counting for segregation analysis of T-DNA loci
Source: Plant Methods. 2025 Jun 24;21:87. doi: 10.1186/s13007-025-01406-4 (PMC12186423; doi:10.1186/s13007-025-01406-4)
Supplement: Supplementary file 2 — Supplementary Material 2 [file 13007_2025_1406_MOESM2_ESM.pdf]

Supplementary Materials for

SeedSeg: Image-based transgenic seed counting for gene segregation analysis of T-DNA loci

Santiago Hernández[1*], Vivian Zhong[2*], Jennifer A. N. Brophy[2+]

Correspondence to: jbrophy@stanford.edu

This PDF file includes:

Supplementary Figure 1

Supplementary Table 1

Supplementary Data File Collection 1

Supplementary Data File Collection 2

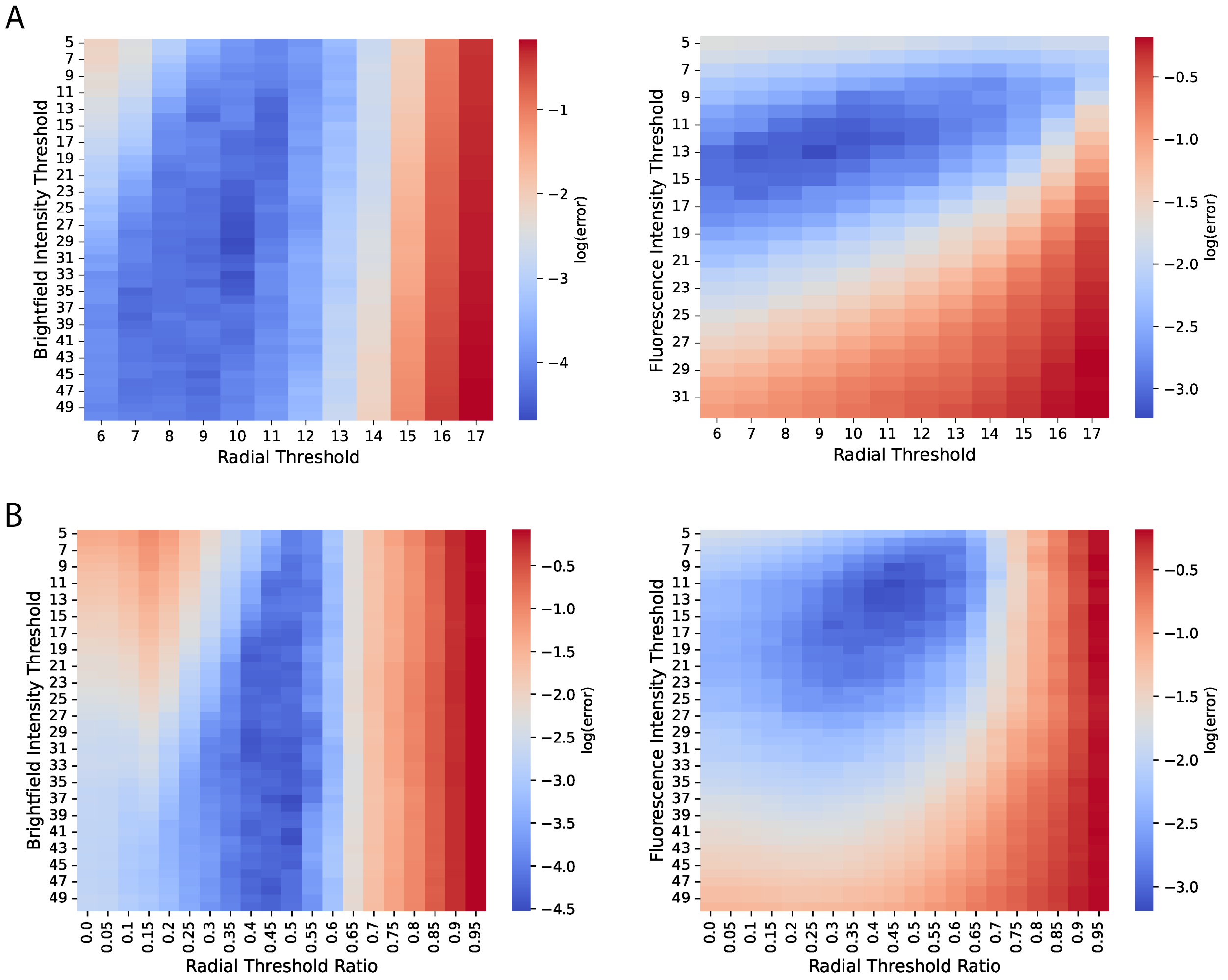

Supplementary Figure 1: SeedSeg accuracy across image intensity and radial threshold parameters. Error in seed count measurements for 28 pairs of fluorescent (right) and brightfield (left) images as the image intensity or radial thresholds are varied. Results are similar when directly setting A) the radial threshold versus setting B) the radial threshold ratio. Error was calculated as the mean percent error from the true seed count. Heatmap was generated using the seaborn Python package.

sample	Manual counts		SeedSeg				PlantCV			
	#seeds BF	#seeds FL	#seeds BF	%error BF	#seeds FL	%error FL	#seeds BF	%error BF	#seeds FL	%error FL
**1**	132	89	132	0	89	0	132	0	89	0
**2**	131	83	131	0	83	0	131	0	83	0
**3**	156	121	156	0	121	0	154	0.0128	115	0.0496
**4**	140	105	140	0	105	0	143	0.0214	104	0.0095
**5**	209	205	209	0	205	0	213	0.0191	182	0.1122
**6**	190	132	190	0	132	0	192	0.0105	134	0.0152
**7**	165	139	165	0	139	0	167	0.0121	131	0.0576
**8**	112	92	112	0	92	0	112	0	93	0.0109
**9**	134	102	134	0	102	0	128	0.0448	92	0.098
**10**	80	55	80	0	55	0	80	0	55	0
**11**	154	123	154	0	123	0	157	0.0195	117	0.0488
**12**	154	117	154	0	117	0	152	0.013	117	0
**13**	52	37	52	0	37	0	51	0.0192	36	0.027
**14**	165	146	165	0	146	0	165	0	127	0.1301
**15**	84	64	84	0	64	0	83	0.0119	61	0.0469
**16**	134	98	134	0	92	0.0612	134	0	84	0.1429
**17**	111	70	111	0	70	0	110	0.009	67	0.0429
**18**	97	74	97	0	74	0	96	0.0103	74	0
**19**	68	55	68	0	55	0	70	0.0294	52	0.0545
**20**	97	77	97	0	77	0	97	0	73	0.0519
**21**	156	115	156	0	115	0	158	0.0128	113	0.0174
**22**	178	125	178	0	125	0	179	0.0056	114	0.088
**23**	94	73	94	0	73	0	94	0	72	0.0137
**24**	104	73	104	0	73	0	103	0.0096	69	0.0548
**25**	140	99	140	0	99	0	143	0.0214	96	0.0303
**26**	68	49	68	0	49	0	68	0	46	0.0612
**27**	97	71	97	0	71	0	98	0.0103	69	0.0282
**28**	161	115	161	0	115	0	149	0.0745	106	0.0783

Table 1: SeedSeg and PlantCV Performance. Error in seed count measurements for 28 pairs of fluorescent (A) and brightfield (B) images. For this comparison, SeedSeg brightfield intensity thresholds, fluorescence intensity thresholds, and radial thresholds were varied from 5 to 49, 5 to 49, and 6 to 19, respectively with a step size of 1 to find optimum parameters for each

image. PlantCV parameters were varied from 100 to 250 with a step size of 10 (brightfield intensity threshold), 10 to 80 with a step size of 5 (fluorescence intensity), and 10 to 1000 with a step size of 100 (size threshold) to find optimum parameters for each image. The percent error was calculated for each image by comparing actual seed counts to model outputs with optimum parameters.

Supplementary Data File Collection 1: Images of FastRed and wild type Arabidopsis used to assess SeedSeg accuracy across parameters (Supplementary Fig 1).

Supplementary Data File Collection 2: Images of Seeds used to assess SeedSeg generalizability (Figure 3).
